# Physiological stage-dependent effects of *Mycobacterium tuberculosis* on human placental tissue: insights into early reactivation and immune modulation

**DOI:** 10.3389/fmicb.2025.1682405

**Published:** 2025-12-10

**Authors:** Monica Selena Fonseca-Perez, Oscar Villavicencio-Carrisoza, Orly Grobeisen-Duque, Luis Antonio Herrera-Moro-Huitron, Arturo Flores-Pliego, Aurora Espejel-Nuñez, Andrea Olmos-Ortiz, Belen Betsaida del-Castillo-Hernandez, Jose Ramon Rojo-Garcia, Sandra Rivera-Gutierrez, Jorge Francisco Cerna-Cortes, Veronica Zaga-Clavellina, Maria Isabel Villegas-Mota, Diana Angelica Aguilar-Ayala, Maria Yolotzin Valdespino-Vazquez, Addy Cecilia Helguera-Repetto

**Affiliations:** 1 Departamento de Inmunobioquímica, Instituto Nacional de Perinatología Isidro Espinosa de los Reyes, Mexico City, Mexico; 2 Escuela Nacional de Ciencias Biológicas, Instituto Politécnico Nacional, Mexico City, Mexico; 3 Departamento de Medicina Traslacional, Instituto Nacional de Perinatología Isidro Espinosa de los Reyes, Mexico City, Mexico; 4 Departamento de Anatomía Patológica, Instituto Nacional de Perinatología Isidro Espinosa de los Reyes, Mexico City, Mexico; 5 Dirección de Investigación, Instituto Nacional de Perinatología Isidro Espinosa de los Reyes, Mexico City, Mexico; 6 Secretaría de Salud del Estado de Quintana Roo, Chetumal, Mexico; 7 Nuffield Department of Women's and Reproductive Health NDWRH, John Radcliffe Hospital, University of Oxford, Oxford, United Kingdom

**Keywords:** congenital tuberculosis, reactivation from hypoxia, placental infection, placental disruption, Mtb virulence, cytokines expression, metalloproteinases action

## Abstract

**Background:**

Tuberculosis (TB) poses a significant risk during pregnancy and the postpartum period, with evidence linking these stages to an increased likelihood of progression from latent TB infection to active disease. Although TB has been associated with adverse pregnancy outcomes, including congenital transmission, the mechanisms by which *Mycobacterium tuberculosis* (Mtb) affects placental structure and function remain poorly understood.

**Objective:**

This study aimed to investigate the stage-dependent effects of Mtb infection on human placental tissue and assess the potential for vertical transmission using an *ex vivo* placental infection model.

**Methods:**

Human term placental explants were infected *ex vivo* with Mtb H37Rv in logarithmic (log) phase and in reactivated dormancy phases (rNRP1 and rNRP2). Bacterial viability was evaluated by CFU quantification at 4, 24, and 48 h post-infection. Histological changes were assessed with hematoxylin-eosin staining; bacilli were visualized using Kinyoun staining and immunofluorescence. Cytokine secretion was measured via multiplex ELISA assays, and Mtb gene expression was analyzed by RT-qPCR.

**Results:**

Mtb in rNRP1 and rNRP2 phases replicated efficiently within placental explants, with CFU increasing by more than one log at 48 h. rNRP2 exhibited delayed tissue entry (only 4% at 24 h), suggesting distinct virulence dynamics based on bacterial phase. Both reactivated phases induced villitis, stromal fibrosis, and reduced vascular integrity, with rNRP2 causing the most severe tissue damage. *rpf*B was significantly upregulated during reactivation (14-fold in rNRP1, 7-fold in rNRP2 at 24 h). Dormancy genes (*dos*R, *hsp*X, *icl*1) and stress-response markers (*sig*H, *whi*B3), were over-expressed in rNRP1, suggesting Mtb remain metabolically equipped to withstand host stresses during early reactivation. Cytokine analysis revealed lower pro-inflammatory responses in rNRP1-infected tissue, while rNRP2 and log-phase Mtb triggered stronger metalloproteinase activity.

**Conclusion:**

Mtb can infect, persist, and replicate within human placental tissue, compromising its structural and immune integrity. These effects vary with the bacterial physiological phase, with early-reactivated Mtb showing the greatest capacity for tissue dissemination and damage. These findings underscore a dual risk of placental injury and increased potential for vertical transmission during early reactivation, emphasizing the need for timely TB screening and intervention during pregnancy.

## 1 Introduction

Tuberculosis (TB) is an infectious disease caused by Mtb, an intracellular bacillus that primarily affects the lungs, although it can involve various extrapulmonary sites (World Health Organization, 2025).

Active tuberculosis (ATB) can result from either a primary infection or the reactivation of latent tuberculosis infection (LTBI), a state in which Mtb persists asymptomatically within the host. Approximately 5%−10% of individuals with LTBI develop active disease through reactivation, particularly in immunocompromised patients that suffer from conditions such as HIV/AIDS, diabetes, cancer, or in children under 5 years of age (Rahlwes et al., 2023).

Infection typically begins when airborne Mtb bacilli reach the pulmonary alveoli, where they are phagocytosed by alveolar macrophages, dendritic cells, and epithelial cells. This interaction initiates an immune response that leads to the formation of granulomas, organized cellular structures that contain the infection. However, in the context of immunosuppression, granulomas may break down or liquefy, enabling bacterial replication, systemic dissemination, and transmission to new hosts (Rahlwes et al., 2023).

*M. tuberculosis* has developed immune- evasive strategies that enable its persistence within the host. Components of its cell wall, such as mycolic acids, TMM/TDM, PDIM, and PIM/LAM, interact with receptors such as TLR2, Mincle, and DC-SIGN, suppressing IL-8, inducing TNF-α/IL-6, blocking autophagy, and preventing phagosomal acidification. Furthermore, it secretes virulent proteins (PE/PPE, EsxA, SapM, PknG) that modulate TLR2 activity, alter phagosome maturation, and regulate cytokine production, thus contributing to the evasion of the immune response. In parallel, Mtb exhibits remarkable metabolic plasticity that favors its survival in hostile conditions. It utilizes lipids and cholesterol as carbon sources via pathways such as the glyoxylate cycle (Icl) and β-oxidation (fadA5, kshA/B), and stores triacylglycerols (Tgs1) during latency. Under stress, non-replicative subpopulations emerge, regulated by networks such as DosR and RelA, and protected by proteins such as HspX and Acr2. This combination of immune evasion and metabolic adaptation enables Mtb to maintain chronic infections and facilitate their transmission (Rahlwes et al., 2023; Joshi et al., 2021).

Tuberculosis poses a significant risk during pregnancy and is associated with adverse maternal and neonatal outcomes, including preterm birth, low birth weight, and perinatal mortality. As TB primarily affects young adults, women of reproductive age are particularly vulnerable. Moreover, pregnancy and the early postpartum period have been linked to an increased risk of progression from LTBI to active disease (Weinberg et al., 2021; Saha et al., 2025). Pregnancy induces dynamic immunological changes, including a progressive reduction in TNF-α secretion by natural killer (NK) cells, particularly during the second and third trimesters. This immune-modulation may hinder the containment of Mtb and mirrors the elevated TB risk seen in patients treated with TNF-α inhibitors. Although findings on the risk of active TB during pregnancy remain heterogenous, several studies indicate a higher incidence in the postpartum period, likely due to delayed diagnosis or immune reconstitution following delivery (Jonsson et al., 2020).

One of the consequences of TB during pregnancy is fetal/neonatal transmission. Congenital tuberculosis is a rare but potentially fatal condition in which the neonate acquires the infection either in utero or during delivery. In contrast to postnatal tuberculosis, congenital form involves vertical transmission of the bacillus from mother to fetus or neonate. Three principal routes of congenital transmission have been proposed: the first is the hematogenous transplacental route, whereby Mtb crosses the placenta or umbilical cord and initiates hepatic lesions with potential systemic dissemination in the neonate. The second is by aspiration or ingestion of infected amniotic fluid, typically resulting in pulmonary and/or gastrointestinal symptoms. The third and last postulated route is by perinatal transmission through contact with infected maternal genital secretions during labor (Wolf et al., 2019; Reyes et al., 2020).

Nevertheless, congenital tuberculosis poses a significant public health challenge due to its high neonatal mortality rate if not promptly recognized and treated. Furthermore, it underscores the critical importance of early detection and appropriate management of tuberculosis in pregnant women as a preventive measure (Malhame et al., 2016; Yeh et al., 2019; Li et al., 2019; Rodriguez-Molino et al., 2020; Solaz Escrig et al., 2022). Therefore, in this article we aim to better understand early reactivation and to explore its clinical implications during placental infection and a possible vertical transmission through a placental infection model. Furthermore, we aim to analyze the physiological stage-dependent effects of Mtb on human placental tissue to have a better insight into the way early reactivation and immune modulation affect the maternal-fetal health during the pathogenesis and development of the disease in pregnant patients.

## 2 Methodology

### 2.1 Study approval and sample collection

This study was approved by the Institutional Review Board and Ethics Committee of the Instituto Nacional de Perinatología Isidro Espinosa de los Reyes (INPer), Mexico (protocol code 2017-2-75, approved on February 7, 2018).

We conducted a basic science, descriptive study using human placental explants as an *ex vivo* model for congenital Mtb infection. Placentas were obtained via cesarean section from uncomplicated, full-term pregnancies (37–40 weeks of gestation) at INPer. Inclusion criteria included placentas obtained from patients aged 18–36 years who underwent elective cesarean delivery due to previous cesarean section, suspected fetal macrosomia, abnormal fetal presentation (breech, transverse, or oblique lie), or maternal pelvic abnormalities. All participants had pregnancies of 38–39 weeks of gestation and provided written informed consent. Apgar scores of 8 or higher at both 1 and 5 min were considered for placenta inclusion.

Exclusion criteria included any history of endocrine, metabolic, infectious, or systemic diseases such as hypertension, diabetes mellitus, or thyroid disorders, and gestational age of < 38 weeks and >39 weeks.

The entire placenta was sampled in the obstetric operating room, and central cotyledons were selected according to the requirements of each experiment.

### 2.2 Bacterial strain and growth conditions

Mtb H37Rv was cultured in Dubos medium (Difco, BD, NJ, USA) supplemented with 0.01% cholesterol (Sigma-Aldrich, MA, USA) and 10% bovine serum albumin (BSA). To induce dormancy, exponential-phase cultures were subjected to hypoxic conditions following the protocol described by Wayne and Hayes (1996), allowing the generation of non-replicating persistence (NRP) stages.

NRP1 was achieved after 5 days of incubation at 37 °C in sealed flasks, while NRP2 was reached after 20 days under the same conditions, as previously reported by Soto-Ramirez et al. (2017).

### 2.3 *In vitro* reactivation of *Mycobacterium tuberculosis*

Dormant Mtb bacilli from the *in vitro* NRP1 and NRP2 phases were reactivated by aerobic ventilation. Cultures were incubated at 37 °C with constant agitation at 160 rpm (Biobase, Wolfenbüttel, Germany) under aerobic conditions for 1 h, as described by Soto-Ramirez et al. (2017).

### 2.4 Placental explant culture and infection

Placental cotyledons were thoroughly washed with sterile 0.9% NaCl (PiSA, CDMX, Mexico) to remove blood clots, blood vessels, decidua, and the chorionic basal plate. The cleaned cotyledons were then cut into 0.5 cm^3^ fragments (corresponding to 100 mg wet-weight), placed in 24-well culture plates, and maintained for 24 h in RPMI medium (Gibco, NY, USA) supplemented with 10% fetal bovine serum (FBS; Biowest, Nuaillé, France) and 1 × antibiotic-antimycotic solution (Gibco), in a humidified incubator (Panasonic, Osaka, Japan) at 37 °C with 5% CO_2_ and 95% air. This initial 24-h incubation period was necessary to allow the placental tissue to stabilize following mechanical manipulation. After stabilization, placental cotyledons were washed with sterile saline solution to remove residual FBS.

Infections were performed using Mtb (H37Rv) at a concentration of 1 × 106 CFU/mL, using bacilli in three different metabolic states: exponential or logarithmic (log) phase, and reactivated non-replicating persistence phases (rNRP1 and rNRP2). Explants were exposed to the bacteria in RPMI medium supplemented with 3% FBS. Uninfected explants were processed in parallel as negative controls. For each assay, three explants from the same placenta were used, and all experiments were independently repeated using three different placentas at separate time points.

At 4, 24, and 48 h post-infection, samples were collected for various analyses. Culture supernatants were frozen for subsequent quantification of cytokines, matrix metalloproteinases (MMPs), and extracellular bacterial viability. A subset of explants was frozen for intracellular viability assays and RNA isolation. Additional explants were fixed in 37% formalin (Hycel, Estado de México, Mexico), embedded in paraffin using a HistoCore Arcadia automated tissue processor (Leica Biosystems, Nussloch, Germany) for 16 h, and processed for histological analysis.

**Figure 1:**
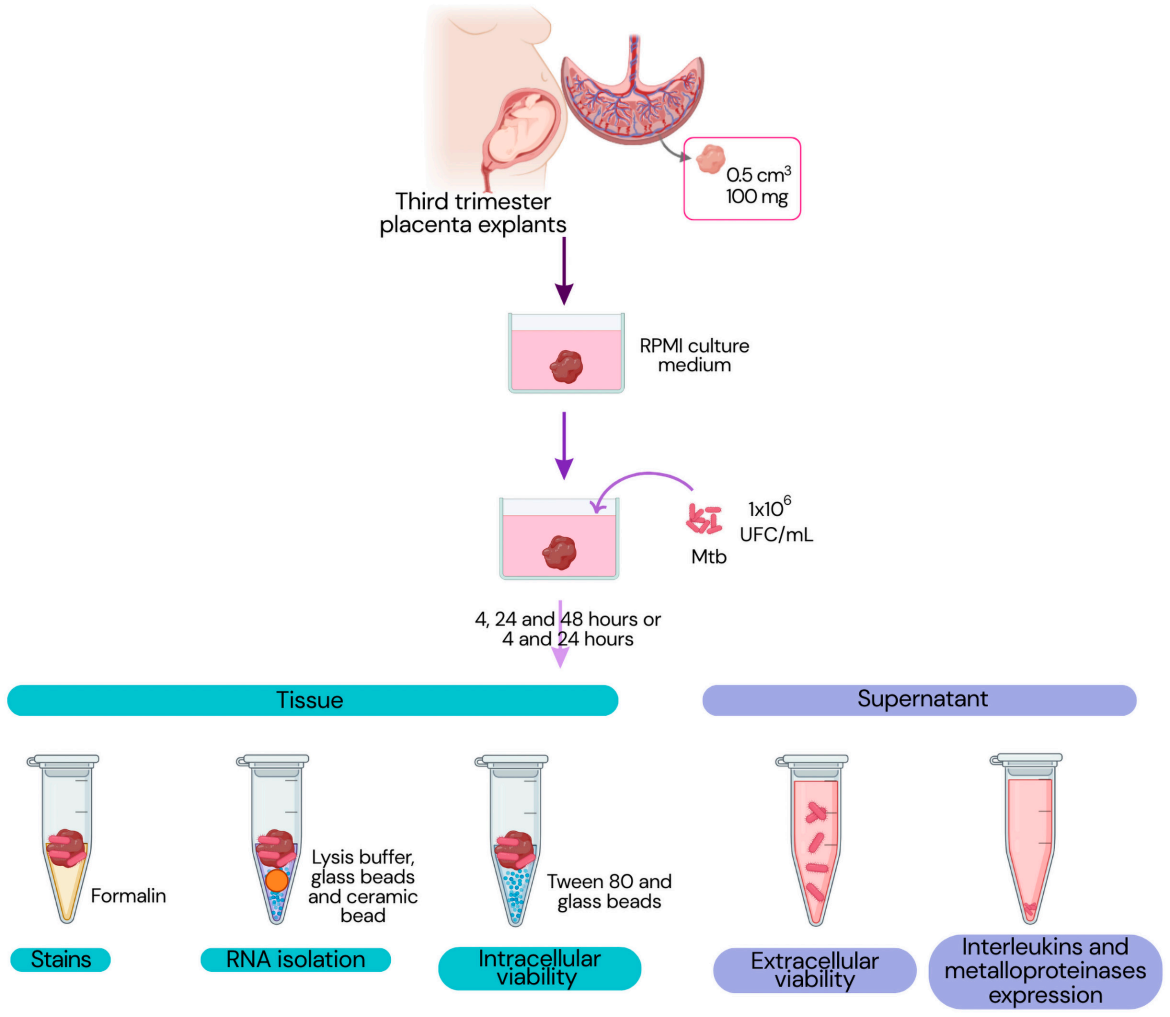
Experimental design. The workflow illustrates which materials, placental tissue or supernatant, were used for each set of experiments.

Figure 1 presents the experimental workflow followed in the next steps.

### 2.5 Viability test

To assess the viability of extracellular (free) Mtb, previously collected culture supernatants were used. Serial 10-fold dilutions (nine in total) were prepared, and 10 μL of each dilution was plated in triplicate on Middlebrook 7H10 agar (BD) using the micro-drop technique described by Miles et al. (1938).

To quantify intracellular Mtb, placental explants were homogenized using 151–212 μm glass beads in a vortex mixer for 30 s. From the homogenate, 100 μL was mixed with 900 μL of 0.05% Tween 80 as diluent. Serial 1:10 dilutions were then prepared (nine in total), and 10 μL of each dilution was plated in triplicate on Middlebrook 7H10 agar, as previously described.

Plates were incubated at 37 °C and monitored daily until visible colonies appeared. Colony counting was performed using a light microscope (Zeiss, Jena, Germany) with 4 × or 10 × objectives. The dilution yielding between 2 and 10 colonies was selected for quantification, and the number of colony-forming units per milliliter (CFU/mL) was calculated using the following formula:


$$\begin{array}{l} CFU/mL=Number\;of\;colonies\;\left(\frac{1}{dilution}\right)\left(\frac{1}{aliquot}\right) \end{array}$$

### 2.6 RNA isolation and cDNA synthesis

Infected placental tissue was transferred to a sterile screw-cap tube containing 300 μL of 151–212 μm glass beads and a 1.4 mm ceramic bead. Tissue lysis and homogenization were performed using the FastPrep-24 homogenizer (MP Biomedicals, New Zealand), applying three 15 s cycles at maximum speed (6.5 m/s), with 5-min incubations on ice between each cycle.

After homogenization, samples were centrifuged at 12,000 × g for 5 min at 4 °C. Total RNA was then extracted using the RNeasy Mini Kit (Qiagen, Thermo Fisher, MA, USA) according to the manufacturer's instructions.

cDNA was prepared using 100 ng of total RNA, using the RevertAid First Strand cDNA synthesis kit (Thermo Fisher) following manufacturer instructions.

### 2.7 Real-time PCR

Eight genes associated with dormancy and stress responses were quantified using real-time quantitative PCR (RT-qPCR) with gene-specific primers (listed in Table 1). Primers were designed using the Primer-BLAST Designer v3.0 software. Reactions were performed with the Maxima SYBR Green/ROX qPCR Master Mix (Thermo Fisher Scientific) in a QuantStudio 5 Real-Time PCR System (Thermo Fisher).

**Table 1:** RT-qPCR primer sequences and target gene information.

**Gene**	**Sequence 5^′^ → 3^′^**	**Annealing temperature (°C)**	**Function**	**Product Tm (°C)**	**References**
*dos*R	F: CGCCTCGATGGTCTGGTG R: CACGATAGCGCGTAGGGTTG	55	It encodes a central transcriptional regulator of the two-component system DosR/DosS-DosT, which allows the bacteria to adapt to hypoxic stress conditions and other environmental stimuli.	83.8	Badillo-Lopez et al., 2010
*hsp*X	F: AGCAGAAGGACTTCGACGGTC R: GTGCGAACGAAGGAACCGTA	58	It encodes a heat shock protein that protects the bacteria from protein damage while reducing bacterial metabolism, thereby promoting latency and prolonged survival. Additionally, its expression modulates the immune response, facilitating immune evasion.	79.2	Badillo-Lopez et al., 2010
*rpf*B	F: CCGCAATCGGATCAAGAA R: CGACCTCCCGGCTCAT	62	RpfB (resuscitation-promoting factor) functions as an endo-1,4-glucanase, degrading cell wall components to release peptidoglycan fragments that trigger cell division. Consequently, it plays a key role in the reactivation of mycobacteria from dormancy.	85.7	Soto-Ramirez et al., 2017
*rrs*	F: ATGACGGCCTTCGGGTTGTAA R: CGGCTGCTGGCACGTAGTTG	58	16S ribosomal RNA is a fundamental component of the 30S subunit of the bacterial ribosome. Its expression serves as a reference for normalization in gene expression analysis.	82.2	Badillo-Lopez et al., 2010
*icl*1	F: CTACAACTGCTCGCCATCGTTC R: CATGGCTGCCAGCTCCTTC	56	It encodes a key enzyme in the glyoxylate cycle, an alternative metabolic pathway that enables bacteria to endure nutritional stress by utilizing lipids as a carbon source instead of carbohydrates.	81.8	Soto-Ramirez et al., 2017
*whi*B3	F: CGATCCCATGCGTTAGAGGT R: CATGGTGCCCTTGAGGAGTA	58	WhiB3 is crucial for metabolic adaptation and virulence, regulating the expression of genes involved in lipid metabolism and the synthesis of virulence factors, including cell envelope lipids.	83.2	This study
*sig*H	F: GCCGCTGTTTCTTGCGATAG R: CCAGGAGACGATGGTGAAGG	58	SigH controls the expression of stress-response genes, including the chaperones *dna*K and *clp*B, as well as antioxidant enzymes such as AhpC and KatG. These proteins are essential for protecting the bacteria against reactive oxygen species (ROS) and reactive nitrogen species (RNS).	81.6	This study
Rv2744c	F: GGCGTTGTTGTATTCGGTGG R: GCGTCAATTGGAGATGCGAC	58	Regulates the number of lipid droplets and their size and is involved in the bacilli persistence during stress.	86.8	This study
*esx*A	F: ATGACAGAGCAGCAGTGGAA R: CAAGGAGGGAATGAATGGAC	60	ESAT-6, secreted by the type VII ESX-1 secretion system, plays a critical role in bacterial virulence. Its primary function is to facilitate immune evasion by disrupting the phagosomal membrane, allowing the bacteria to escape degradation within macrophages. Additionally, ESAT-6 inhibits phagosome-lysosome fusion, further enhancing bacterial survival.	81.2	Soto-Ramirez et al., 2017

PCR conditions consisted of an initial hold at 95 °C for 15 min, followed by 30 amplification cycles of 15 s at 95 °C (denaturation), 15 s at the gene-specific annealing temperature (Table 1), and a 15 min final extension at 72 °C. All primers were used at a final concentration of 200 nM.

Primer specificity was confirmed by melting curve analysis. Absolute mRNA levels were normalized to 16S rRNA expression, which served as the internal reference. Differential gene expression was assessed by comparing expression levels in reactivated NRP stages (rNRP1 and rNRP2) with those obtained from log-phase infections.

For relative expression analysis, values were plotted using a baseline value of 1 on the x-axis to indicate equivalent expression between conditions; deviations above or below this reference reflected upregulation or downregulation, respectively.

### 2.8 Kinyoun staining

Paraffin-embedded placental explants were sectioned at a thickness of 1.5 μm using a microtome (Leica) and deparaffinized by incubation at 70 °C in an oven (Leica) for 20 min, followed by gradual rehydration through a descending alcohol series.

Kinyoun staining was performed by covering the tissue sections with Carbol Fuchsin solution for 30 min. Excess stain was removed by rinsing with water followed by decolorization with acid alcohol solution for 30 s. After a second rinse, sections were counterstained with Methylene Blue for 1 min to visualize cellular components. Slides were mounted using Entellan™ New mounting medium (Merck) and examined under a Zeiss Axiolab 5 optical microscope (Zeiss, Jena, Germany) at 100 × magnification.

### 2.9 Immunofluorescence staining

Paraffin-embedded placental tissue sections were subjected to antigen retrieval was performed by immersing the slides in a retrieval buffer (1 × PBS, 10 mM Tris, 1 mM EDTA, and 0.05% Tween-20, pH 9.0) and subjecting them to 1 kg/cm^2^ of pressure at 130 °C for 5 min. After cooling to room temperature, the slides were washed briefly with PBS. Subsequently, 75 μL of a permeabilization solution (1 × PBS with 0.5% Triton X-100) was added. The slides were incubated in a humid chamber for 30 min. After washing with 1 × PBS for 5 min, 75 μL of blocking solution (1 × PBS, 0.01% Triton X-100, and 10% FBS) was applied, followed by 1-h incubation in the humid chamber at room temperature.

After a second PBS wash, slides were incubated with two primary antibodies: MA1-06315 (Thermo Fisher), targeting cytokeratin 7 (CK-7), a marker of trophoblasts, at a 1:100 dilution; and ab20962 (Abcam, Cambridge, UK), a conjugated antibody (FITC 495) specific for Mtb, at a 1:50 dilution.

Following incubation, the slides were washed in the dark with 1 × PBS under gentle agitation (120 rpm) for 30 min, and subsequently incubated with a conjugated secondary antibody (A10521, Thermo Fisher; Cyanine 3) at a 1:100 dilution for 1 h at room temperature in the dark. The secondary antibody was applied exclusively for the detection of the CK-7 primary antibody. Samples were then air-dried at room temperature, and 3 μL of Vectashield Antifade Mounting Medium with DAPI (Vector Laboratories, CA, USA) was added. Imaging was performed using an Olympus IX73 fluorescence microscope (Olympus, Tokyo, Japan) at 40× magnification.

### 2.10 Quantification of cytokines and metalloproteinases

Cytokine quantification (GM-CSF, IFN-γ, IL-1β, IL-2, IL-4, IL-5, IL-6, IL-8, IL-10, and TNF-α) was performed using the commercial Cytokine 10-Plex Human Panel (Thermo Fisher). Metalloproteinases (MMP-2, MMP-3, and MMP-9) were quantified using the Human MMP Panel 2, 3-Plex (Thermo Fisher), following the manufacturer's instructions. Both panels were analyzed using a Luminex system from BIO-RAD (CA, USA).

Cytokine and metalloproteinase concentrations were interpolated using a standard curve, with all measurements performed in triplicate.

### 2.11 Statistical analysis

Sigma Stat v.3.5 (CA, USA) was used to assess statistical significance using one-way ANOVA. Comparative analysis of quantitative data from infection phases relative to the uninfected control was conducted using ANOVA followed by Dunnett's *post hoc* test (*P* < 0.050). Additionally, comparisons between infection phases or infection times were performed using ANOVA with the Holm-Sidak *post hoc* method (*P* < 0.050).

## 3 Results

### 3.1 Reactivated *Mycobacterium tuberculosis* shows enhanced growth and adaptation in placental tissue

**Figure 2:**
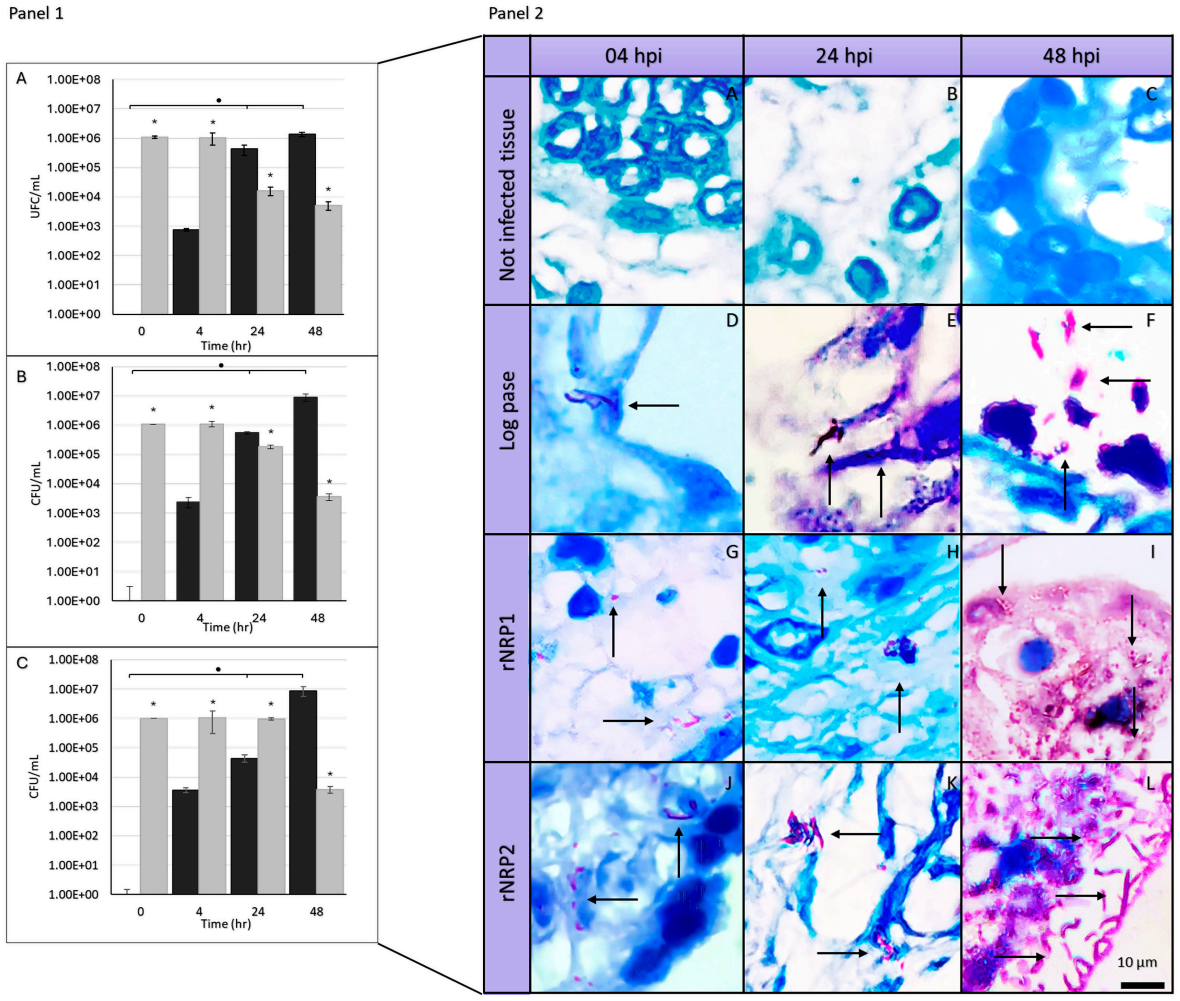
Viability and observation of *Mycobacterium tuberculosis* in placental explants. Panel 1. Colony-forming units (CFU) of Mtb during infection kinetics. **(A)** Infection at logarithmic growth phase, **(B)** infection at rNRP1 phase, and **(C)** infection at rNRP2 phase. Placental explants (0.5 cm3, ~100 mg) were infected with 1 × 106 Mtb CFU. CFU were quantified both in the supernatant (gray bars) and within the tissue (black bars). Data were analyzed using one-way ANOVA followed by Dunnett's test: *p < 0.05 compared to control, and •*p* < 0.05 for comparisons between tissue and supernatant CFUs. Panel 2. Light micrographs of placental explants stained with Kinyoun stain (100× magnification). Cell nuclei appear dark blue, extracellular matrix is light blue, and bacteria are pink (indicated by arrows). Rows indicate the time of infection, while columns show Mtb metabolic phase. **(A–C**) Uninfected controls; **(D–F)** Explants infected with Mtb in logarithmic growth phase; **(G–I)** Explants infected with Mtb in early reactivation (rNRP1); **(J–L)** Explants infected with Mtb in late reactivation (rNRP2). Bacilli density per microscopic field progressively increases over time, with the highest CFU observed in both reactivated Mtb infections.

Figure 1 shows multiplication of bacilli in our model, also, Kinyoun staining is included. When analyzing the ability of Mtb to grow and multiply within placental tissue (Figure 2, Panel 1), we observed that infection with log-phase Mtb did not result in an increase in total colony forming units (CFU) over the 48 h course (Figure 2 Panel 1A). In contrast, infections with reactivated NRP1 and NRP2 Mtb showed bacterial multiplication within the tissue model at 48 h, with CFU increasing by more than one log (from 1.07 × 106 to 2.11 × 107 CFU/mL) (Figures 2 Panel 1B, C). This suggests that Mtb in reactivated phases may adapt more efficiently to the model compared to log-phase bacteria.

When assessing Mtb entry and multiplication within the tissue, we detected bacilli as early as 4 h post-infection, even though CFU/mL at this time point represented less than 1% of the total viable bacteria (Figures 2 Panel 1A–C). By 24 h post-infection, almost all log-phase and rNRP1-phase Mtb were found within the tissue, representing 99% and 75% of viable bacteria, respectively (Figures 2 Panel 1A, B). Interestingly, reactivated NRP2-phase Mtb showed a markedly different pattern, with only 4% of total counted bacteria localized within the tissue at this time (Figure 2 Panel 1C), that may support the notion that Mtb virulence is influenced by its reactivation phase. Despite these differences at the earlier point, by 48 h post-infection, all three phases reached 99% of viable cells within the placental tissue.

To assess the presence and relative abundance of bacteria during placental infections, different sections of the explants were obtained. Kinyoun staining was performed to specifically visualize bacilli, while immunofluorescence staining was used to potentially localize bacteria within the trophoblasts.

Figure 2 Panel 2 shows light micrographs of placental explants stained with Kinyoun stain, in which the first row (Figures 2 Panel 2A–C) displays uninfected placental tissue controls, where trophoblast cell nuclei appear deep blue. Consistent with the CFU quantification results described earlier, acid-fast bacilli (marked by a black arrow) were detected as early as 4 h post-infection, though at low levels, across all three Mtb phases (Figures 2 Panel 2D, G, J). Furthermore, the presence and distribution of Mtb in its rNRP1 and rNRP2 phases progressively increased throughout the study (Figures 2 Panel 2H, I, K, L), aligning with the CFU quantification results (Supplementary Figures S1–S12, S70 present individual images acquired directly from the microscope).

Immunofluorescence microscopy images, shown in Figure 3 (and in Supplementary Figures S13–S57, S71–S73 show time-dependent infection images with individual fluorescence filters, displayed before cropping and editing), are consistent with optical images obtained by Kinyoun staining. Infection controls (Figures 3A–C) confirmed the accuracy of the experiments, ensuring that they were performed correctly and without cross-contamination (see Supplementary Figures S71–73). At 4 h post-infection (Figures 3D–F), only a few bacteria (marked with a white arrow) were detected in all three phases. Their number and distribution increased to 24 h (Figures 3G–I), reaching their maximum concentration at 48 h (Figures 3J–L).

**Figure 3:**
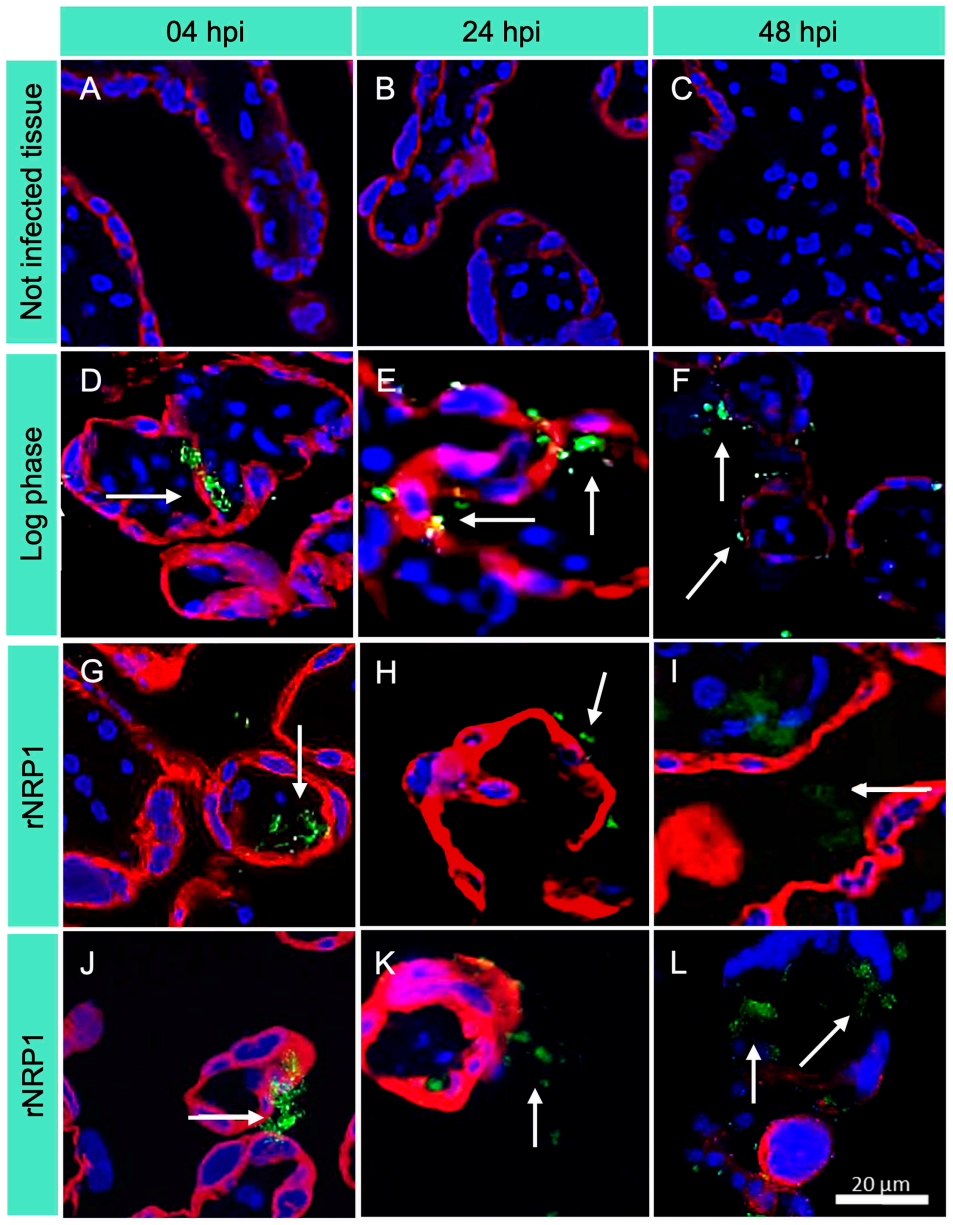
Immunofluorescence images of placental explants infected with *Mycobacterium tuberculosis*. CK7 = trophoblasts (red); Mtb = FITC (green); DAPI = nuclei (blue). Rows indicate the time of infection, while columns show Mtb metabolic phase. 40× magnification was used. **(A–C)** Uninfected controls; **(D–F)** Explants infected with Mtb in log phase; **(G–I)** Explants infected with Mtb at rNRP1 stage; **(J–L)** Explants infected with Mtb at rNRP2 stage. The green fluorescence in infected explants increases over time, indicating a higher mycobacterial load, particularly in the reactivated phases.

### 3.2 Log-phase and rNRP2 *Mycobacterium tuberculosis* phases induce progressive placental tissue damage

**Figure 4:**
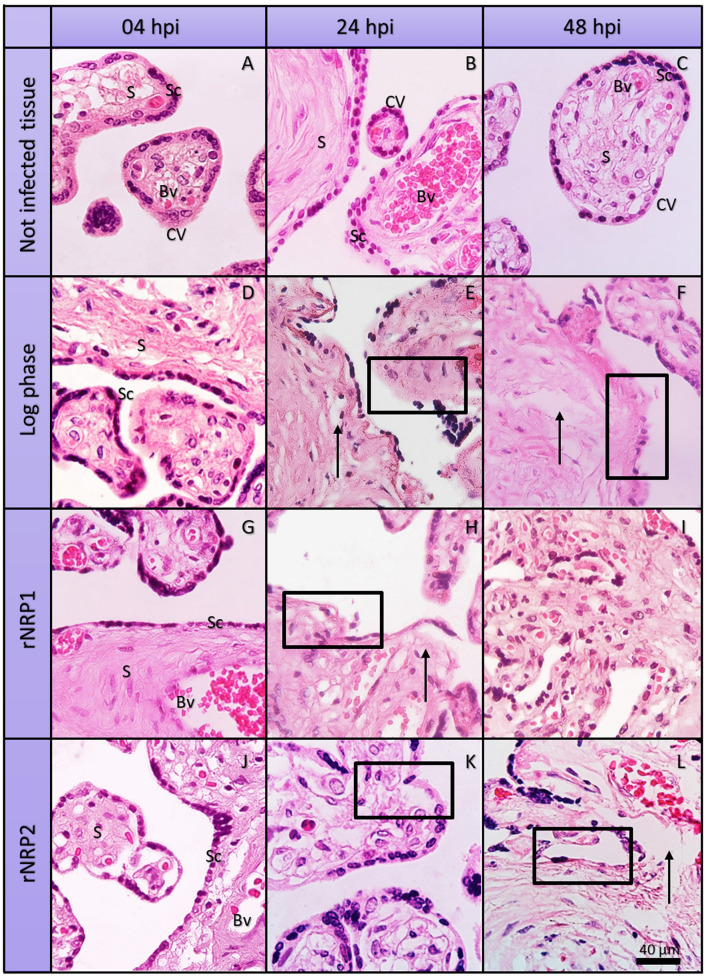
Histopathological alterations induced by *Mycobacterium tuberculosis* in human placental explants. Representative micrographs of hematoxylin and eosin (HE)-stained histological sections from non-infected placental explants **(A–C)** and explants infected with Mtb in logarithmic phase **(D–F)**, early reactivation phase **(G–I)**, and late reactivation phase **(J–L)**, Rows indicate the time of infection, while columns show Mtb metabolic phase. All observations were conducted at 40× magnification. CV, Chorionic villi; Sc, syncytiotrophoblast layer; S, stroma; Bv, blood vessels. No evidence of inflammation or tissue damage is observed in uninfected controls while at 24 hpi focal loss of Sc continuity (black box) and initial fragmentation of collagen fibers (black arrow) are evident in log and rNRP2 experiments.

**Figure 5:**
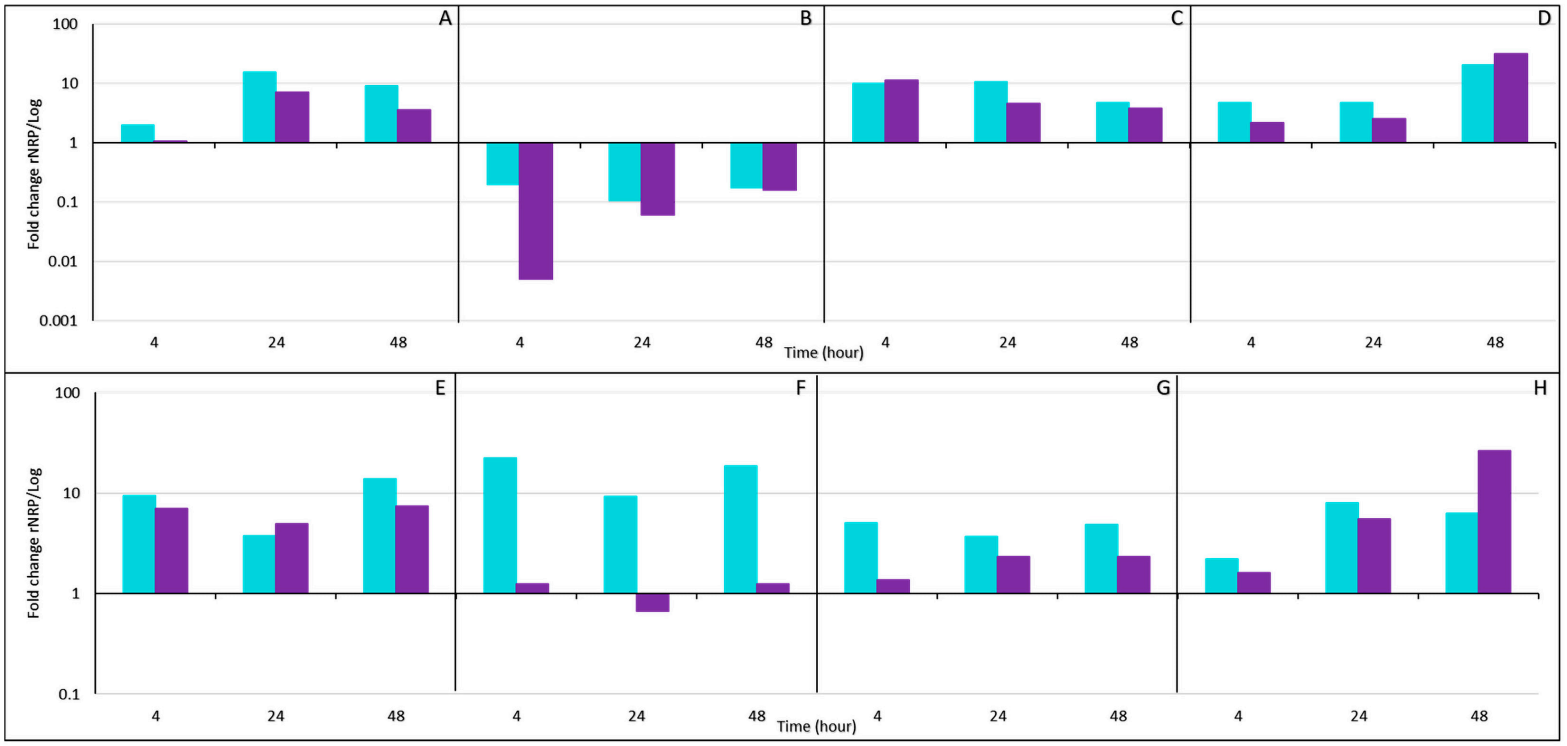
Differential expression profiles of reactivated rNRP1 and rNRP2 phases relative to the logarithmic phase, during placental infection model. Quantitative analysis of the relative expression of Mtb genes in rNRP1 and rNRP2 phases, normalized to 16S rRNA expression and compared to the obtained during the logarithmic phase infection (rNRP expression relative to log-phase expression). **(A)** *rpf*B, **(B)** *esx*A, **(C)** *hsp*X, **(D)** *dos*R, **(E)** *icl*1, **(F)** *sig*H, **(G)** *whi*B3, and **(H)** Rv2744c. Reactivated Mtb exhibited higher expression levels of traditional dormancy-associated markers than log-phase bacteria.

To assess the morphological changes potentially induced by the presence of mycobacteria and by the possible predominance of a pro-inflammatory cytokine profile, we performed hematoxylin-eosin (HE) staining (Figure 4; Supplementary Figures S58–S69, S74 present individual images acquired directly from the microscope). Under healthy physiological conditions, third-trimester placental tissue displayed well-preserved chorionic villi (Figure 4A: CV). The syncytiotrophoblast (Sc) formed a continuous outer layer surrounding the villi, while the underlying stroma (S) consisted of fibroblasts, collagen fibers, and scattered immune cells. Blood vessels (Bv) appeared widely distributed within the villous core, with clearly visible erythrocytes. Notably, at 24 h and even at later time points (Figures 4B, 5C), no significant signs of stromal degradation, inflammatory cell infiltration, or syncytiotrophoblast disruption were observed. These findings suggest that normal placental integrity and physiology were maintained throughout the study period (Supplementary Figure S5 shows the images before cropping and editing).

In contrast, infection with log-phase Mtb resulted in progressive tissue degradation over time (Figures 4D–F). At 4 h post-infection (Figure 4D), the structural integrity of the villi remained largely unaltered. However, by 24 h (Figure 4E), a noticeable disruption of the syncytiotrophoblast layer was observed (highlighted by a black frame), particularly at the villous periphery. Within the stromal compartment, collagen fibers showed early signs of degradation (black arrow), accompanied by increased immune cell infiltration. These structural alterations became even more pronounced at 48 h post-infection (Figure 4F), characterized by extensive syncytiotrophoblast disruption and pronounced stromal disorganization, suggesting progressive tissue damage driven by the infection.

Placental explants infected with the reactivated phase of early latency (Figures 4G–I) initially exhibited no significant structural changes at the first evaluation time point (Figure 4G). However, as the infection progressed, a disruption in syncytiotrophoblast continuity and an increase in infiltrating immune cells within the chorionic villi became evident (Figure 4H). By 48 h post-infection (Figure 4I), the chorionic villi displayed signs of villitis and intervillitis, accompanied by a marked thinning of the villous structure. Additionally, while blood vessels remained present, their overall size was noticeably reduced.

Similarly, infection with the rNRP2 phase (Figures 4J–L) initially does not induce physiological alterations compared to the uninfected control tissue at 4 h post-infection (Figure 4J). However, by 24 h (Figure 4K), disruptions in syncytiotrophoblast continuity, tissue degradation, morphological changes in blood vessels, and villitis are observed. These alterations become more pronounced at 48 h (Figure 4L), with increased tissue degradation and the emergence of stromal fibrosis (marked with a black arrow).

### 3.3 Early *rpfB* induction reveals rapid reactivation dynamics of rNRP1 *Mycobacterium tuberculosis*

To better understand Mtb potential virulence and ability to multiply within placental tissue, that may have caused the morphological changes observed, the differential expression of Mtb across the three phases was analyzed (Figure 5). For this aim, the quantification of *rpf*B expression was crucial, due to the *in vitro-*reactivated dormancy phases model involved in our infection. This gene encodes a resuscitation-promoting factor that degrades peptidoglycan during bacillary reactivation. As expected, both reactivated phases showed *rpf*B overexpression compared to log-phase Mtb. Notably, the highest expression level was observed at 24 h post-infection, with rNRP1 and rNRP2 exhibiting 14-fold and 7-fold increases, respectively (Figure 5A). However, rNRP2 appeared to require more time for full reactivation. At 4 h post-infection, it showed only a 1-fold increase in *rpf*B expression compared to log-phase Mtb, whereas rNRP1 had already reached a 2-fold increase at this time point (Figure 5A).

Since our infection model involved *in vitro*-reactivated dormancy phases, quantifying *rpf*B expression was crucial. This gene encodes a resuscitation-promoting factor that degrades peptidoglycan during bacillary reactivation. As expected, both reactivated phases showed *rpf*B overexpression compared to log-phase Mtb. Notably, the highest expression level was observed at 24 h post-infection, with rNRP1 and rNRP2 exhibiting 14-fold and 7-fold increases, respectively (Figure 5A).

### 3.4 Reactivated *Mycobacterium tuberculosis* phases downregulate the antigen gene *esxA*

Subsequently, quantification of the expression of *esx*A was analyzed, a gene encoding an early secretion antigen. Compared to the log-phase Mtb, both reactivated phases exhibited downregulated *esx*A expression. Specifically, rNRP1 showed a 5-fold lower expression, while rNRP2 exhibited a dramatic 194-fold reduction (Figure 5B). Interestingly, rNRP2 gradually increased *esx*A expression over time, reaching a 6.23-fold lower expression than the log phase at 48 h post-infection. In contrast, rNRP1 maintained a consistently low and homogeneous *esx*A expression throughout the experiment (Figure 5B).

### 3.5 Dormancy marker genes remain highly expressed in reactivated *M. tuberculosis* during placental infection

The quantification of the three genes traditionally associated with latency, commonly referred to as dormancy markers (*hsp*X, *dos*R, and *icl*1), was also made. We observed an overexpression of *hsp*X in both reactivated phases compared to log-phase Mtb, with similar expression levels at 4 h post-infection, where it also reached its expression peak (10-fold and 11-fold higher than log phase for rNRP1 and rNRP2, respectively) (Figure 5C). Furthermore, the highest expression of *dos*R was detected at 48 h post-infection (Figure 5D), while *icl*1 reached its maximum expression at 4 and 48 h post-infection (Figure 5E).

### 3.6 rNRP1 exhibits strong stress-response and persistence gene activation

Additionally, *sig*H, a mycobacterial sigma factor involved in transcriptional regulation under stress conditions, was analyzed. The expression of this gene varied significantly between the two reactivated phases. In rNRP1, *sig*H was consistently overexpressed, ranging from 9.2-fold to 22.3-fold higher than in log-phase Mtb (Figure 5F). In contrast, rNRP2-infected placental tissue showed only a slight overexpression at 4 and 48 h post-infection (1-fold increase compared to the log phase). Notably, at 24 h post-infection, *sig*H expression in rNRP2 decreased by 1.5-fold compared to the log phase (Figure 5F).

Finally, we analyzed the expression of two genes associated with mycobacterial persistence and virulence, *whi*B3 and Rv7244c. *whi*B3 was more highly expressed in rNRP1 compared to both rNRP2 and log-phase Mtb, with its peak expression reaching only 5-fold higher than the log phase. In contrast, rNRP2 exhibited a more modest increase, reaching a 2.3-fold higher expression compared to the log phase (Figure 5G). Moreover, Rv7244c increased its expression in both reactivated phases along time compared to log phase (Figure 5H).

### 3.7 Mtb promote phase-specific MMP activation, reflecting differential host tissue remodeling and inflammatory potential

In addition to the impact of Mtb virulence factors on the tissue, certain host-derived molecules may also contribute to cellular disruption and the morphological changes observed. Therefore, we investigated the expression of various matrix metalloproteinases as part of the host response.

**Figure 6:**
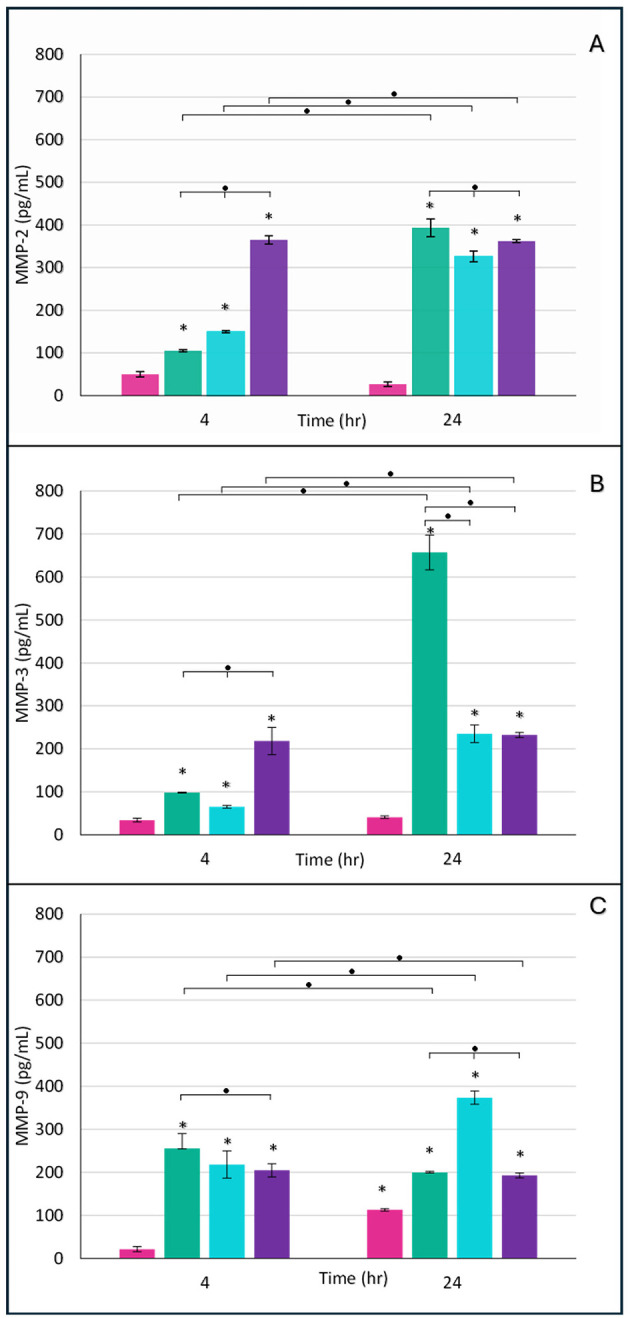
Expression of secreted metalloproteinases in placental explants infected with *M. tuberculosis* at different physiological phases. Significant increases in MMP secretion were observed in the infected groups compared to the control, with variations depending on the time post-infection and the physiological phase of the bacillus. **(A)** MMP-2 expression, **(B)** MMP-3 expression, and **(C)** MMP-9 expression. Pink bars, uninfected control; green bars, logarithmic phase; blue bars, rNRP1 phase; and purple bars, rNRP2 phase. MMP2 showed particularly high early expression in the rNRP2 group; MMP-3 showed its highest secretion at 24 h in the logarithmic phase of infection, and MMP-9 increased steadily in all infected groups, with rNRP1 being the most prominent at 24 h. The data were subjected to statistical analysis using one-way ANOVA with Dunnett's correction. *P* < 0.05 for comparisons against controls (*) and between times or phases at the same time (•).

First MMP-2 quantitation was analyzed, which was minimal in uninfected control tissue compared to infected explants (Figure 6A). At 4 h post-infection, the rNRP2-infected group exhibited a sevenfold increase in MMP-2 expression compared to control cultures, the highest observed level. In contrast, log-phase infected tissue showed a twofold increase, while rNRP1-infected explants exhibited a threefold rise, both relative to uninfected tissue. By 24 h post-infection, MMP-2 secretion reached similarly high levels across all infected tissues, regardless of the Mtb phase (Figure 6A).

Next, MMP-3 expression was detected (Figure 6B). At the earliest point, all three infection phases exhibited slightly elevated MMP-3 levels compared to uninfected controls, with statistically significant differences. Among them, rNRP2 induced the highest initial expression. By 24 h post-infection, log-phase Mtb triggered the highest MMP-3 expression, reaching 656.97 pg/mL a three-fold increase compared to both reactivated dormancy phases (Figure 6B) and 15.9 times higher than uninfected controls.

Finally, we examined MMP-9 expression (Figure 6C). At 4 h post-infection, explants infected with any of the three bacterial phases exhibited MMP-9 levels ranging from 204.6 to 255.4 pg/mL, representing an approximately 10-fold increase compared to uninfected controls. By 24 h, MMP-9 levels in uninfected explants had increased but remained significantly lower than in infected groups. Notably, rNRP1-infected explants displayed 1.8- and 1.9-fold higher MMP-9 expression than log-phase and rNRP2-infected groups, respectively (Figure 6C).

### 3.8 Log-phase and rNRP2 Mtb provoke strong proinflammatory cytokine production, while rNRP1 elicits IL-6 dominant response

We also quantified the cytokine immune response triggered by the infection. While some cytokines exhibited basal expression in uninfected controls, their levels were significantly lower than those observed in infected samples.

**Figure 7:**
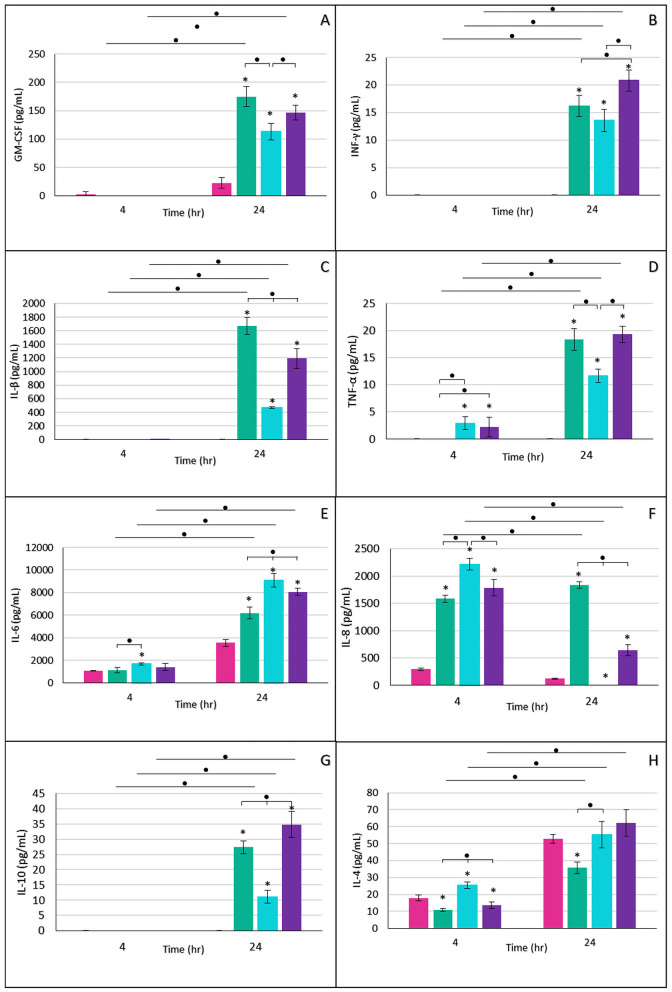
Expression of proinflammatory and immunomodulatory cytokines in placental explants infected with *M. tuberculosis* at different stages. Levels of GM-CSF **(A)**, IFN-γ **(B)**, IL-1β **(C)**, TNF-α **(D)**, IL-6 **(E)**, IL-8 **(F)**, IL-10 **(G)**, and IL-4 **(H)** were quantified as part of the early immune response after infection. Pink bars, uninfected control; green bars, logarithmic phase; blue bars, rNRP1 phase; and purple bars, rNRP2 phase. Statistical analysis was performed using one-way ANOVA with Dunnett's correction for comparisons against the control (^*^) and between time points or physiological phases at the same time point (•).

Proinflammatory cytokines, including granulocyte-macrophage colony-stimulating factor (GM-CSF) (Figure 7A), interferon-gamma (IFN-γ) (Figure 7B), interleukin-1 beta (IL-1β) (Figure 7C), and tumor necrosis factor-alpha (TNF-α) (Figure 7D), exhibited similar expression patterns. At 4 h post-infection, their expression remained undetectable, except for TNF-α (Figure 7D), which reached approximately 2 pg/mL in reactivated-phases infections. By 24 h post-infection, cytokine levels peaked; however, rNRP1 induced lower cytokine production compared to the other two Mtb phases (Figures 7A–D).

Unlike other proinflammatory cytokines, IL-6 was detected in both uninfected and infected samples at 4 h post-infection, with a statistically significant increase observed only in rNRP1-infected explants (1.5-fold). By 24 h post-infection, IL-6 levels rose significantly across all infection phases compared to the control, with rNRP1-infected explants exhibiting the highest expression of this cytokine (Figure 7E).

At 4 h post-infection, the highest IL-8 expression (Figure 7F) was observed in reactivated phases, but levels declined by 24 h. In rNRP1-infected explants, IL-8 was abated entirely at this time point, while rNRP2-infected samples showed a 2.8-fold reduction. In contrast, log-phase Mtb infection led to a significant increase of nearly 300 pg/mL between 4 and 24 h. These findings highlight the crucial role of IL-8 in bacterial containment and cellular recruitment.

### 3.9 Log-phase and rNRP2 Mtb induce the strongest expression of the immunomodulatory cytokine IL-10 in placental tissue

At 24 h post-infection, the immune-modulatory cytokine IL-10 (Figure 7G) was exclusively detected in placental explants. Its highest expression was induced by the reactivated from NRP2 phase, showing a 1.2-fold increase compared to log-phase infection and a 3.1-fold increase compared to rNRP1-infected explants.

The anti-inflammatory cytokine IL-4 naturally increases during healthy pregnancy. As expected, IL-4 expression was detected in uninfected controls at both time points (Figure 7H). Infected tissues exhibited similar or lower IL-4 levels compared to uninfected controls at both 4 and 24 h post-infection.

## 4 Discussion

Between a quarter and a third of the world's population is estimated to be latently infected with Mtb. According to the World Health Organization, approximately 5%−10% of those will develop active tuberculosis during their lifetime due to reactivation of the pathogen. Since reactivated TB is contagious, eliminating latent infection is considered a cornerstone of global TB control. Consequently, a deeper understanding of the latent infection and its reactivation is a recognized research priority (Bayer and Castro, 2017). However, while LTBI has received considerable attention, the mechanisms and dynamics of reactivation remain relatively unexplored and should be prioritized to the same extent.

Reactivation risk is highest in individuals recently infected, particularly close contacts of active TB cases who may have up to 15 times greater risk of developing active disease (Ai et al., 2016). Thus, the majority of TB burden likely stems from recent transmission rather than long-standing latent infection (Behr et al., 2018).

The Centers for Disease Control and Prevention (CDC) in 2025 emphasized specific considerations for diagnosing and treating latent and active TB during pregnancy. In general, treatment for latent TB can be postponed until 2–3 months postpartum. However, women at high risk of progressing to active TB require close monitoring throughout pregnancy to prevent maternal and neonatal complications ( Tuberculosis (TB) | CDC, 2025).

In this context, we analyzed placental infection using two experimental models of TB reactivation: one representing early reactivation (following reoxygenation after the NRP1 hypoxia stage), and another representing late reactivation (reoxygenation after the NRP2 stage). Both models were compared to an acute infection model using log-phase Mtb. Our aim was to better understand why reactivation may occur soon after infection and to explore its clinical implications during placental infection and a possible vertical transmission.

### 4.1 *Mycobacterium tuberculosis* invades and replicates within the human placenta

Vertical transmission is a well-recognized route of infection for several pathogens, including bacteria capable of breaching the placental barrier (Sheridan et al., 2017; Garcia-Mendez et al., 2019; Huang et al., 2022; Megli and Coyne, 2022; Lorea et al., 2025; Gerstenberg et al., 2014). Our findings demonstrate that Mtb can invade placental tissue at all evaluated time points. Bacilli were detected as early as 4 h post-infection, with bacterial counts increasing by approximately 48% after 48 h. Notably, 99% of the bacteria were localized intracellularly, indicating efficient internalization and potential replication within permissive placental cells. These results underscore the high susceptibility of placental tissue to Mtb infection and highlight the need for further investigation of host–pathogen interactions at the maternal–fetal interface, particularly during early gestation using isolated cytotrophoblast models.

The precise mechanisms mediating Mtb recognition and uptake by placental cells remain to be fully elucidated. It is known that bacterial entry into host cells depends on several pattern-recognition receptors, that recognize mycobacterial components such as lipoarabinomannan (LAM) (Kireev et al., 2025). Trophoblasts express many of these receptors, including CD206 (mannose receptor) and DC-SIGN (CD209), suggesting that they may play a key role in facilitating bacillary adhesion and invasion at the maternal–fetal interface (Motomura et al., 2023).

### 4.2 Histopathological evidence linking *Mycobacterium tuberculosis* infection to placental dysfunction

Histological analysis (H&E staining) revealed that Mtb induces progressive morphological alterations in human placental tissue *ex vivo*, depending on its physiological phase. Infection with bacilli in the logarithmic or late-latency reactivation phase resulted in syncytiotrophoblast disruption, immune cell infiltration, stromal disorganization, fibrosis, and lesions consistent with villitis and intervillitis. In contrast, infection with early-reactivated Mtb produced only minimal alterations at 24 h, which became evident by 48 h.

These observations are consistent with clinical reports of placental tuberculosis, which describe acute inflammatory infiltrates rich in CD68^+^ macrophages and neutrophils but lacking granuloma formation features that contribute to structural damage of the chorionic villi (Hu et al., 2023). Such an innate immune-dominated response likely promotes tissue destruction and compromises placental function.

Clinically, these histopathological alterations may impair barrier integrity, gas exchange, and nutrient transport, leading to complications such as intrauterine growth restriction, spontaneous preterm birth, and fetal death. Moreover, fibrosis and vascular disorganization associated with advanced infection can cause chronic fetal hypoxia (Salafia et al., 2024, 2010; Amelio et al., 2022). Although not directly observed in this study, chronic placental infections such as tuberculosis may also contribute to preeclampsia through sustained inflammation, trophoblast dysfunction, and impaired angiogenesis (Hu et al., 2023; Redline, 2008; Simpson et al., 2023). Altogether, our results suggest that adverse pregnancy outcomes associated with tuberculosis may be driven, at least in part, by infection-induced placental damage.

The persistence and dissemination of Mtb depend on the coordinated expression of multiple factors that facilitate its survival under host-imposed stress conditions. Hypoxia is established as a stress signal and as a cue for bacterial “dormancy” development within tuberculous granulomas in experimental models of TB as well as in human lesions. Very little is known about the molecular mechanism of its reactivation from persistence, especially during early stages of the reactivation.

### 4.3 Early-reactivated *Mycobacterium tuberculosis* shows enhanced regrowth capacity relative to late-reactivated forms

Previous observations suggest that Mtb lag phase (adaptation to reactivation) is associated with a global gene expression reprogramming that defines the initiation of a reactivation process. Resuscitation factors (Rpf) play a critical role in stimulating growth through cell wall remodeling (Tufariello et al., 2006). As expected, both Mtb reactivated phases over-expressed *rpf*B gene in contrast with the log phase. Interestingly, we found an initial 2-fold times more expression of this gene only in rNRP1, suggesting that it is easier for Mtb to resume its growth from early dormancy. Iona et al. described the increase of *rpf* A and *rpf*B transcription since 1 h after aeration of the media, showing their importance for the Mtb recovery to active growing (Iona et al., 2016). Also, Du et al. (2016) found that reactivation from an *in vitro* late dormancy phase took 2 days of lag phase without bacilli growing demonstrating that Mtb in late dormancy requires more time to re-adapt and resume its growing. Clinically, although we know that reactivation can occur decades after infection (Lillebaek et al., 2002), it has been reported that 80% of reactivation cases occurs in the first 5 years after Mtb dormant state (Dale et al., 2021); other work demonstrated a median time of reactivation in contacts of actively Mtb infected patients of 1.26 years, with a probability of developing active disease of 45% at 1 year after latency, 62% after 2 years and 83% of risk at 5 years (Borgdorff et al., 2011).

### 4.4 Reactivated *Mycobacterium tuberculosis* appears to be better equipped to withstand stress conditions and may therefore persist more efficiently within the host

After infection occurs, Mtb has to adapt to the stress conditions in order to survive and persist. One strategy is the expression of *esx*A gene, which encodes a virulence protein ESAT6 (6 kDa early secreted antigen) (Passos et al., 2024). It has been demonstrated that EsxA is upregulated during Mtb infection, and gene deletion or the impaired secretion of EsxA leads to a reduction in Mtb intracellular survival (Bao et al., 2021a). There is evidence showing that Mtb blocks a series of phagosome maturation events that occur after phagocytosis and ESAT6 secretion is involved in it (Jiang et al., 2024). Additionally, regulates immune system and promotes autophagy (Liu et al., 2017). Our experiments showed lower expression of this gene in both Mtb reactivated phases respect log phase presenting its maximum level at 24 h. This result might be explained by the fact that this early secreted antigen helps bacteria to reach a persistence state and once its function is reached, this antigen is down regulated (Iona et al., 2016). This result suggest that our reactivated bacilli don't need the over-expression of this gene (as log phase Mtb does) probably as they are now prepared to survive or might be pre-adapted to the host stress conditions.

To better understand dormancy and persistence in Mtb, we analyzed key genes known to mediate adaptation to hostile host environments. DosR is strongly induced under hypoxia and is essential for dormancy, persistence, and drug tolerance (Kumari et al., 2017; Mehra et al., 2015). Although the reactivation process generally represses dormancy genes such as *dosR* (Verma et al., 2021), some hypervirulent strains, like W/Beijing, constitutively express *DosR*, pre-adapting them to stress through the accumulation of triacylglycerols (TAGs) that promote survival (Kumari et al., 2017). HspX, a heat shock protein and molecular chaperone, is induced by DosR under low-oxygen conditions and is crucial for Mtb survival in macrophages (Gopinath et al., 2015; Hu et al., 2006). HspX also contributes to biofilm formation during stress (Rivas-Santiago et al., 2023; Wang et al., 2019). While Gopinath et al. (2015) reported decreased HspX expression during reactivation, we observed elevated *dos*R and *hsp*X levels in both early and late reactivation phases, suggesting reactivated bacilli remain pre-adapted for survival, similar to hypervirulent strains. Other dormancy-associated factor is Icl1, encoding isocitrate lyase, which is central to the glyoxylate shunt and enables Mtb to use fatty acids as carbon sources during infection (Ko et al., 2021; Munoz-Elias and McKinney, 2005). *icl*1 expression increases under hypoxia and during fatty acid metabolism, and *icl*-deficient mutants show survival defects during chronic infection (Dutta and Karakousis, 2014; Bhusal et al., 2017). Interestingly this gene was also over-expressed in reactivated phases. The over-expression of the dormancy-associated genes *dos*R, *hsp*X, and *icl*1 in reactivated Mtb, suggest that bacilli remain metabolically equipped to withstand host stresses, potentially increasing virulence in reactivated Mtb. These finding are also consistent with our previous discussed results showing that Mtb expresses the reactivation factor *rpf*B and proliferates more efficiently in tissue than log-phase bacilli, which are adapting to a stressful environment for the first time.

### 4.5 Early-reactivated *Mycobacterium tuberculosis* exhibited greater preparedness to survive stress conditions and persist within the host

Among the sigma factors involved in oxidative and heat stress responses, and thus in Mtb virulence, SigH play a key role (Wani and Jain, 2024). SigH is critical for long-term infection persistence (Sharp et al., 2016), mainly through activation of thioredoxin (*trx*) genes, essential for redox homeostasis inside macrophages (Sugandhi et al., 2023). In our work, *sig*H expression was >10-fold higher during early reactivation, but similar to log-phase levels during late reactivation, suggesting SigH-regulated proteins are essential throughout Mtb's life cycle and supporting the idea that early reactivating bacilli are more virulent or metabolically prepared. Additionally, it has been demonstrated that *sig*H-regulated genes are upregulated during reactivation and inactivating them impairs recovery from dormancy (Du et al., 2016). Finally, SigH modulates immune responses by promoting secretion of molecules affecting chemotaxis and apoptosis, aiding Mtb survival and spread (Joshi et al., 2021).

Another gene critical for maintaining redox homeostasis and supporting Mtb survival during infection is *whi*B3. Its expression regulates the synthesis of complex lipids, including sulfolipids (SL), polyacyltrehalose (PAT), diacyltrehalose (DAT), and phthiocerol dimycocerosate (PDIM), lipids known to trigger inflammation in the host. Additionally, WhiB3 regulates the synthesis of reserve lipids such as triacylglycerols (TAGs), forming lipid inclusions that support bacillary survival during dormancy (Barrientos et al., 2022; Baker et al., 2019; Goar et al., 2022). WhiB3 also modulates the mycothiol redox system (Mehta et al., 2016), providing another survival strategy within the host (Chen et al., 2024). In our study, *whi*B3 showed marked overexpression during early reactivation, from 4 to 48 h post-infection, while late reactivation showed only a modest twofold increase compared to log phase.

Similarly, Rv2744c, a conserved 35-kDa alanine-rich membrane protein and *psp*A ortholog (Trutneva et al., 2020; Datta et al., 2015), plays a key role in the envelope stress-responsive Phage Shock Protein (Psp) response system, mitigating envelope damage under stress (Datta et al., 2015). Rv2744c is highly expressed during late dormancy (Trutneva et al., 2020; Nikitushkin et al., 2022), and to a lesser extent during reactivation (Schubert et al., 2015). Notably, Rv2744c regulates the number and size of lipid droplets, contributing to Mtb persistence during anaerobically induced dormancy (Armstrong et al., 2016), likely in coordination with WhiB3, as both genes influence lipid metabolism essential for survival during stress. Therefore, the overexpression of both *whi*B3 and Rv2744c observed in our model supports the idea that Mtb reactivating from early dormancy is metabolically primed to limit hostile host environments, enhancing its ability to persist.

We propose that Mtb's ability to persist, and potentially disseminate within the placenta (as it happens on the lung) depends on its gene expression profile, which in turn is influenced by the physiologic phase of the infecting bacilli. It is reasonable to assume that differential expression of Mtb genes related to persistence and virulence would lead to corresponding variations in host MMP and cytokine secretion.

### 4.6 Both log-phase and rNRP2 *Mycobacterium tuberculosis* strains elicit a stronger MMP response in placental tissue

Mtb induces the expression of matrix metalloproteinases (MMP-2, MMP-3, and MMP-9) in the placenta, with levels varying by bacterial metabolic state and time post-infection. This suggests that the placenta responds actively to Mtb through proteolytic pathways involved in tissue remodeling and inflammation (Elkington et al., 2011; Walker et al., 2012). While MMP-2 and MMP-9 are physiologically involved in trophoblast invasion and angiogenesis, and MMP-3 in cervical remodeling (Timokhina et al., 2020), their overexpression has been associated with pregnancy complications such as preeclampsia, infection, and preterm labor (Barron et al., 2024; Mirani et al., 2025).

In our *ex vivo* model, MMP-2 showed a marked increase as early as 4 h post-infection, particularly in explants exposed to late reactivation-phase bacilli (rNRP2), reaching up to sevenfold higher levels than control. This aligns with findings from extrapulmonary TB models, where MMP-2 promotes tissue invasion by degrading type IV collagen (Squeglia et al., 2018). Such early induction may compromise the trophoblast basement membrane, increasing the risk of gestational complications associated with late-reactivated Mtb. MMP-3 exhibited a moderate rise at 4 h and a more robust increase at 24 h, especially with log-phase bacilli, suggesting a cumulative effect that might exacerbate tissue damage during persistent infection (Green et al., 2011). MMP-9 levels also rose early, with a notable peak after 24 h in response to early reactivated Mtb, reflecting an infection-driven inflammatory response.

The MMP findings align with our H&E staining results, which showed that log-phase and rNRP2 bacilli caused the most pronounced tissue damage.

### 4.7 The inflammatory cytokine response seems to restrict log-phase and rNRP2 *M. tuberculosis*, but rNRP1 bacilli might evade or resist this control

Placental TB appears to promote a Th1-skewed proinflammatory environment, characterized by TNF-α and IFN-γ that enhances MMP expression, as seen in Mtb-infected macrophages (Elkington et al., 2011). Notably, in this work the strongest Th1 cytokine responses occurred with log-phase and late-reactivated Mtb, but not with early-reactivated Mtb, as discussed later. This suggests that metabolic changes in the bacillus modulate its immunogenicity and tissue response, which is consistent with studies demonstrating that dormant or reactivated bacilli can selectively evade or alter the host immune response (Lachmandas et al., 2018).

IL-6 and IL-8, in particular, play dual roles: they are crucial for pathogen defense but are also associated with preterm labor and neurodevelopmental disorders. A recent birth cohort study linked high placental and cord blood levels of IL-6, IL-8, and TNF-α with motor and social delays at 18 months (Zheng et al., 2024). Moreover, Hofbauer cells actively secrete IL-6 and IL-8 in response to bacterial endotoxins, indicating their immunological responsiveness (Reyes and Golos, 2018).

In our model, early-reactivated Mtb induced IL-6 and IL-8 secretion alongside a mild proinflammatory profile (moderate IFN-γ, TNF-α, IL-1β, GM-CSF), possibly reflecting an immune containment strategy with limited tissue damage (as stated before). This pattern aligns with *in vivo* data showing that EsxA, a key Mtb virulence factor, induces strong cytokine production (including IFN-γ, IL-6, IL-8, and TNF-α) in PBMCs from infected donors (Blevins et al., 2022). Consistently, we found the highest *esx*A expression and cytokine induction in log-phase Mtb infection.

The granulocyte-monocyte colony-stimulating factor (GM-CSF) is a well-known regulator of fetal growth (Westergaard et al., 2024). Its overexpression has been associated with inflammatory pregnancy disorders such as preeclampsia (Kurmanova et al., 2025; Jancsura et al., 2025), as well as with maternal infections caused by HIV and CMV (Schuch et al., 2024). In the context of tuberculosis, GM-CSF has been reported during pulmonary infection and even proposed as a salivary biomarker (Selvan et al., 2025). These findings reinforce the notion that Mtb infection may exert a significant inflammatory impact during pregnancy, particularly during the log phase or late reactivation, potentially contributing to adverse outcomes.

IFN-γ plays a central role in host immunity against Mtb by activating macrophages to kill intracellular bacilli and by promoting the recruitment of immune cells to infection sites (Bao et al., 2021b). Dutta and colleagues demonstrated that both IFN-γ and TNF-α are essential for resistance to Mtb, and that sustained macrophage activation is critical to prevent reactivation of latent infection. In our study, both IFN-γ and TNF-α were strongly secreted during infections with Mtb in the late reactivation and log phases. This, combined with the elevated levels of other proinflammatory cytokines, suggests an immune response aimed at controlling bacterial growth and limiting spread, potentially reflecting an effective containment strategy at the tissue level for both log and late reactivated Mtb, but not for rNRP1 bacilli.

Both IL-4 and IL-10 are anti-inflammatory cytokines that are highly expressed *in vivo* during latent tuberculosis compared to active disease (Mensah et al., 2025), likely contributing to the control of tissue damage caused by inflammation. IL-4, in particular, increases during pregnancy, promoting an anti-inflammatory environment essential for fetal tolerance. Low IL-4 levels have been associated with adverse outcomes such as preterm birth, spontaneous abortion, fetal growth restriction, preeclampsia, and recurrent miscarriage (Chatterjee et al., 2014). In our study, the reduced IL-4 expression observed during log-phase Mtb infection suggests immunological dysregulation. Combined with the strong proinflammatory profile induced by this phase, these findings point to a higher risk of adverse pregnancy outcomes, likely driven by placental inflammation rather than vertical transmission.

These findings together provide a foundation for future *in vivo* and clinical studies by highlighting how distinct Mtb metabolic states elicit differential host responses during pregnancy and in extrapulmonary infection models. They suggest that MMPs and cytokines could serve as biomarkers of early reactivation or tissue-destructive disease and point to potential therapeutic strategies aimed at modulating host inflammatory responses to prevent placental damage and vertical transmission. Overall, this work links *in vitro* placental infection models with pathophysiological mechanisms relevant to human tuberculosis, supporting translational research toward improved diagnostics and preventive interventions.

### 4.8 Strengths and limitations

Although cases of congenital tuberculosis have been increasingly reported over time, the host–pathogen interaction in this context remains largely unexplored, and the mechanisms underlying vertical transmission are still not fully understood. This study opens a novel line of research that warrants further, in-depth investigation to elucidate the biological processes involved. This study is the first to evaluate *Mycobacterium tuberculosis* infection in a human placental explant model, simultaneously analyzing both the bacillary behavior and the host tissue response. Notably, it also represents the first evidence linking early reactivation of Mtb to enhanced stress adaptation, potentially increasing its virulence during *ex vivo* placental infection. However, certain limitations must be acknowledged. First, all placental explants used were obtained from term pregnancies, suggesting this developmental stage is permissive to Mtb infection. As a result, the susceptibility and response of earlier gestational tissues, such as those from the first or second trimester remain unknown, limiting our understanding of Mtb-placenta interactions across pregnancy. Additionally, because whole tissue explants were used, we cannot distinguish whether the observed responses originated from trophoblasts or other placental cell populations, such as resident macrophages. Moreover, this study employed human placental explants, which, although valuable for controlled mechanistic analyses, cannot fully replicate the physiological complexity of the *in vivo* environment including maternal circulation, systemic immune modulation, and hormonal dynamics. Therefore, these findings should be interpreted with caution, as they have not yet been validated in pregnant patients or animal models, which were beyond the scope of this work. Finally, all placentas were obtained from healthy donors to ensure the use of physiologically normal tissue, allowing us to analyze the infection process and host-pathogen interactions under controlled *in vitro* conditions.

## 5 Conclusions

Our findings highlight that *Mycobacterium tuberculosis* can infect, persist and multiply in the placenta compromising its functional integrity and pose a significant threat to perinatal outcomes. Early detection and timely intervention are essential to prevent serious complications in pregnant women with active or reactivated latent TB.

Mtb has the capacity to modulate the host immune response by downregulating immunogenic antigens, activating stress-response genes and this response appears to depend not only on the host environment but also on the physiologic phase of the bacilli.

Importantly, our results suggest that early-reactivated Mtb may have the highest potential to disseminate within placental tissue, posing a dual risk: contributing to structural damage and increasing the possibility of vertical transmission. Understanding the interaction between bacillary metabolic states and placental immune responses is therefore crucial to improving maternal-fetal health in TB-endemic settings. Further research on this topic is essential to strengthen and support systematic contact tracing of infected patients, particularly in the context of pregnancy, where targeted follow-up may have significant implications for maternal and fetal health.

## Data Availability

The raw data supporting the conclusions of this article will be made available by the authors, without undue reservation.

## Supplementary material

Includes the original images acquired by optical microscopy (Kinyoun and HE staining) and fluorescence microscopy, prior to cropping and adjustment of contrast and brightness. Kinyoun-stained images (Supplementary Figures S1–S12) were obtained at 100 × magnification to allow detailed visualization of bacteria; Mtb appears in magenta (carbol-fuchsin), while surrounding tissue is counterstained in blue (methylene blue). Fluorescence microscopy images (Supplementary Figures S13–S60) were captured at 100 × magnification, using the following channels: DAPI (461 nm, blue, nuclei), CK7 (560 nm, red, trophoblasts), and FITC (519 nm, green, mycobacteria). The channels were subsequently merged. Not infected tissue did not show signal in FITC channel. HE-stained images were acquired at 40× magnification to provide a broader view of tissue architecture. Cropped regions of each image, highlighting areas where Mtb or tissue damage was observed, were used to construct Figures 2–4 in the main manuscript. The final four images were generated from the original captures from microscope to facilitate comparison across infection phases and time points.

Supplementary Figure S1 Light micrographs of non-infected placental explants stained with Kinyoun at baseline (4 h).

Supplementary Figure S2 Light micrographs of non-infected placental explants stained with Kinyoun at 24 hpi.

Supplementary Figure S3 Light micrographs of non-infected placental explants stained with Kinyoun at 48 hpi.

Supplementary Figure S4 Light micrographs of log phase-infected placental explants stained with Kinyoun at baseline (4 h).

Supplementary Figure S5 Light micrographs of log phase-infected placental explants stained with Kinyoun at 24 hpi.

Supplementary Figure S6 Light micrographs of log phase-infected placental explants stained with Kinyoun at 48 hpi.

Supplementary Figure S7 Light micrographs of rNRP1-infected placental explants stained with Kinyoun at baseline (4 h).

Supplementary Figure S8 Light micrographs of rNRP1-infected placental explants stained with Kinyoun at 24 hpi.

Supplementary Figure S9 Light micrographs of rNRP1-infected placental explants stained with Kinyoun at 48 hpi.

Supplementary Figure S10 Light micrographs of rNRP2-infected placental explants stained with Kinyoun at baseline (4 h).

Supplementary Figure S11 Light micrographs of rNRP2-infected placental explants stained with Kinyoun at 24 hpi.

Supplementary Figure S12 Light micrographs of rNRP2-infected placental explants stained with Kinyoun at 48 hpi.

Supplementary Figure S13 Immunofluorescence images obtained at 461 nm (DAPI) of non-infected placental explants at baseline (4 h).

Supplementary Figure S14 Immunofluorescence images obtained at 461 nm (DAPI) of non-infected placental explants at 24 hpi.

Supplementary Figure S15 Immunofluorescence images obtained at 461 nm (DAPI) of non-infected placental explants at 48 hpi.

Supplementary Figure S16 Immunofluorescence images obtained at 560 nm (CK7) of non-infected placental explants at baseline (4 h).

Supplementary Figure S17 Immunofluorescence images obtained at 560 nm (CK7) of non-infected placental explants at 24 hpi.

Supplementary Figure S18 Immunofluorescence images obtained at 560 nm (CK7) of non-infected placental explants at 48 hpi.

Supplementary Figure S19 Merged Immunofluorescence images of non-infected placental explants at baseline (4 h).

Supplementary Figure S20 Merged Immunofluorescence images of non-infected placental explants at 24 hpi.

Supplementary Figure S21 Merged Immunofluorescence images of non-infected placental explants at 48 hpi.

Supplementary Figure S22 Immunofluorescence images obtained at 461 nm (DAPI) of log phase-infected placental explants at baseline (4 h).

Supplementary Figure S23 Immunofluorescence images obtained at 461 nm (DAPI) of log phase-infected placental explants at 24 hpi.

Supplementary Figure S24 Immunofluorescence images obtained at 461 nm (DAPI) of log phase-infected placental explants at 48 hpi.

Supplementary Figure S25 Immunofluorescence images obtained at 560 nm (CK7) of log phase-infected placental explants at baseline (4 h).

Supplementary Figure S26 Immunofluorescence images obtained at 560 nm (CK7) of log phase-infected placental explants at 24 hpi.

Supplementary Figure S27 Immunofluorescence images obtained at 560 nm (CK7) of log phase-infected placental explants at 48 hpi.

Supplementary Figure S28 Immunofluorescence images obtained at 519 nm (FITC) of log phase-infected placental explants at baseline (4 h).

Supplementary Figure S29 Immunofluorescence images obtained at 519 nm (FITC) of log phase-infected placental explants at 24 hpi.

Supplementary Figure S30 Immunofluorescence images obtained at 519 nm (FITC) of log phase-infected placental explants at 48 hpi.

Supplementary Figure S31 Merged Immunofluorescence images of log phase-infected placental explants at baseline (4 h).

Supplementary Figure S32 Merged Immunofluorescence images of log phase-infected placental explants at 24 hpi.

Supplementary Figure S33 Merged Immunofluorescence images of log phase-infected placental explants at 48 hpi.

Supplementary Figure S34 Immunofluorescence images obtained at 461 nm (DAPI) of rNRP1-infected placental explants at baseline (4 h).

Supplementary Figure S35 Immunofluorescence images obtained at 461 nm (DAPI) of rNRP1-infected placental explants at 24 hpi.

Supplementary Figure S36 Immunofluorescence images obtained at 461 nm (DAPI) of rNRP1-infected placental explants at 48 hpi.

Supplementary Figure S37 Immunofluorescence images obtained at 560 nm (CK7) of rNRP1-infected placental explants at baseline (4 h).

Supplementary Figure S38 Immunofluorescence images obtained at 560 nm (CK7) of rNRP1-infected placental explants at 24 hpi.

Supplementary Figure S39 Immunofluorescence images obtained at 560 nm (CK7) of rNRP1-infected placental explants at 48 hpi.

Supplementary Figure S40 Immunofluorescence images obtained at 519 nm (FITC) of rNRP1-infected placental explants at baseline (4 h).

Supplementary Figure S41 Immunofluorescence images obtained at 519 nm (FITC) of rNRP1-infected placental explants at 24 hpi.

Supplementary Figure S42 Immunofluorescence images obtained at 519 nm (FITC) of rNRP1-infected placental explants at 48 hpi.

Supplementary Figure S43 Merged Immunofluorescence images of rNRP1-infected placental explants at baseline (4 h).

Supplementary Figure S44 Merged Immunofluorescence images of rNRP1-infected placental explants at 24 hpi.

Supplementary Figure S45 Merged Immunofluorescence images of rNRP1-infected placental explants at 48 hpi.

Supplementary Figure S46 Immunofluorescence images obtained at 461 nm (DAPI) of rNRP2-infected placental explants at baseline (4 h).

Supplementary Figure S47 Immunofluorescence images obtained at 461 nm (DAPI) of rNRP2-infected placental explants at 24 hpi.

Supplementary Figure S48 Immunofluorescence images obtained at 461 nm (DAPI) of rNRP2-infected placental explants at 48 hpi.

Supplementary Figure S49 Immunofluorescence images obtained at 560 nm (CK7) of rNRP2-infected placental explants at baseline (4 h).

Supplementary Figure S50 Immunofluorescence images obtained at 560 nm (CK7) of rNRP2-infected placental explants at 24 hpi.

Supplementary Figure S51 Immunofluorescence images obtained at 560 nm (CK7) of rNRP2-infected placental explants at 48 hpi.

Supplementary Figure S52 Immunofluorescence images obtained at 519 nm (FITC) of rNRP2-infected placental explants at baseline (4 h).

Supplementary Figure S53 Immunofluorescence images obtained at 519 nm (FITC) of rNRP2-infected placental explants at 24 hpi.

Supplementary Figure S54 Immunofluorescence images obtained at 519 nm (FITC) of rNRP2-infected placental explants at 48 hpi.

Supplementary Figure S55 Merged Immunofluorescence images of rNRP2-infected placental explants at baseline (4 h).

Supplementary Figure S56 Merged Immunofluorescence images of rNRP2-infected placental explants at 24 hpi.

Supplementary Figure S57 Merged Immunofluorescence images of rNRP2-infected placental explants at 48 hpi.

Supplementary Figure S58 Light micrographs of non-infected placental explants stained with Hematoxylin/eosin at baseline (4 h).

Supplementary Figure S59 Light micrographs of non-infected placental explants stained with Hematoxylin/eosin at 24 hpi.

Supplementary Figure S60 Light micrographs of non-infected placental explants stained with Hematoxylin/eosin at 48 hpi.

Supplementary Figure S61 Light micrographs of log phase-infected placental explants stained with Hematoxylin/eosin at baseline (4 h).

Supplementary Figure S62 Light micrographs of log phase-infected placental explants stained with Hematoxylin/eosin at 24 hpi.

Supplementary Figure S63 Light micrographs of log phase-infected placental explants stained with Hematoxylin/eosin at 48 hpi.

Supplementary Figure S64 Light micrographs of rNRP1-infected placental explants stained with Hematoxylin/eosin at baseline (4 h).

Supplementary Figure S65 Light micrographs of rNRP1-infected placental explants stained with Hematoxylin/eosin at 24 hpi.

Supplementary Figure S66 Light micrographs of rNRP1-infected placental explants stained with Hematoxylin/eosin at 48 hpi.

Supplementary Figure S67 Light micrographs of rNRP2-infected placental explants stained with Hematoxylin/eosin at baseline (4 h).

Supplementary Figure S68 Light micrographs of rNRP2-infected placental explants stained with Hematoxylin/eosin at 24 hpi.

Supplementary Figure S69 Light micrographs of rNRP2-infected placental explants stained with Hematoxylin/eosin at 48 hpi.

Supplementary Figure S70 Composed image of Kinyoun staining using the original micrographs.

Supplementary Figure S71 Composed image of Fluorescence microscopy at baseline using the original micrographs.

Supplementary Figure S72 Composed image of Fluorescence microscopy at 24 hpi using the original micrographs.

Supplementary Figure S73 Composed image of Fluorescence microscopy at 48 hpi using the original micrographs.

Supplementary Figure S74 Composed image of HE staining using the original micrographs.
