# Supplementary figures and images for: Physiological stage-dependent effects of Mycobacterium tuberculosis on human placental tissue: insights into early reactivation and immune modulation
Source: Front Microbiol. 2025 Dec 10;16:1682405. doi: 10.3389/fmicb.2025.1682405 (PMC12727939; doi:10.3389/fmicb.2025.1682405)

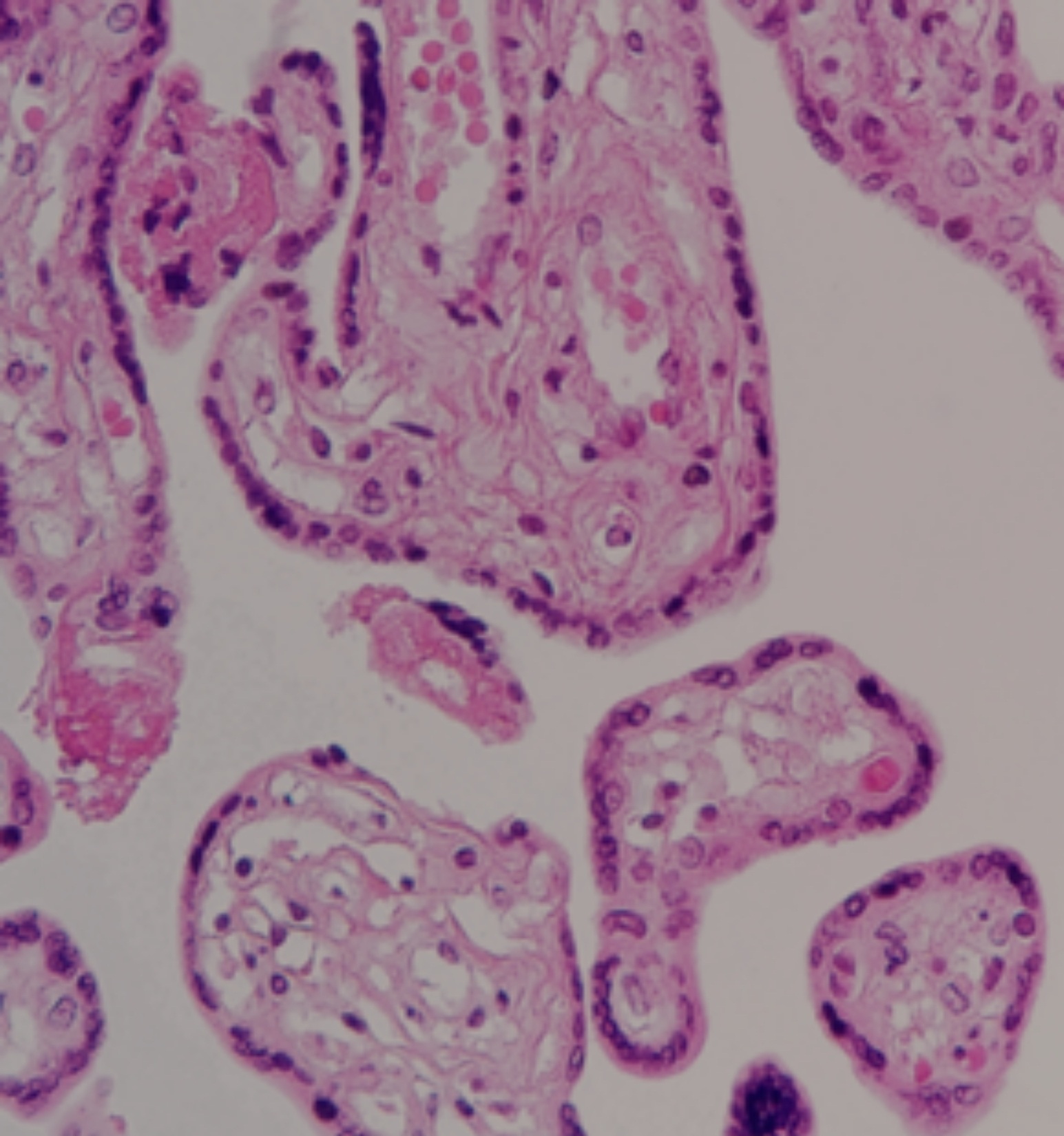

Supplement: Supplementary Figure S1 — Light micrographs of non-infected placental explants stained with Kinyoun at baseline (4 h). [file Data_Sheet_1.zip › Supplementary figures/HE S58-S69, S74/Figure S58.jpg]

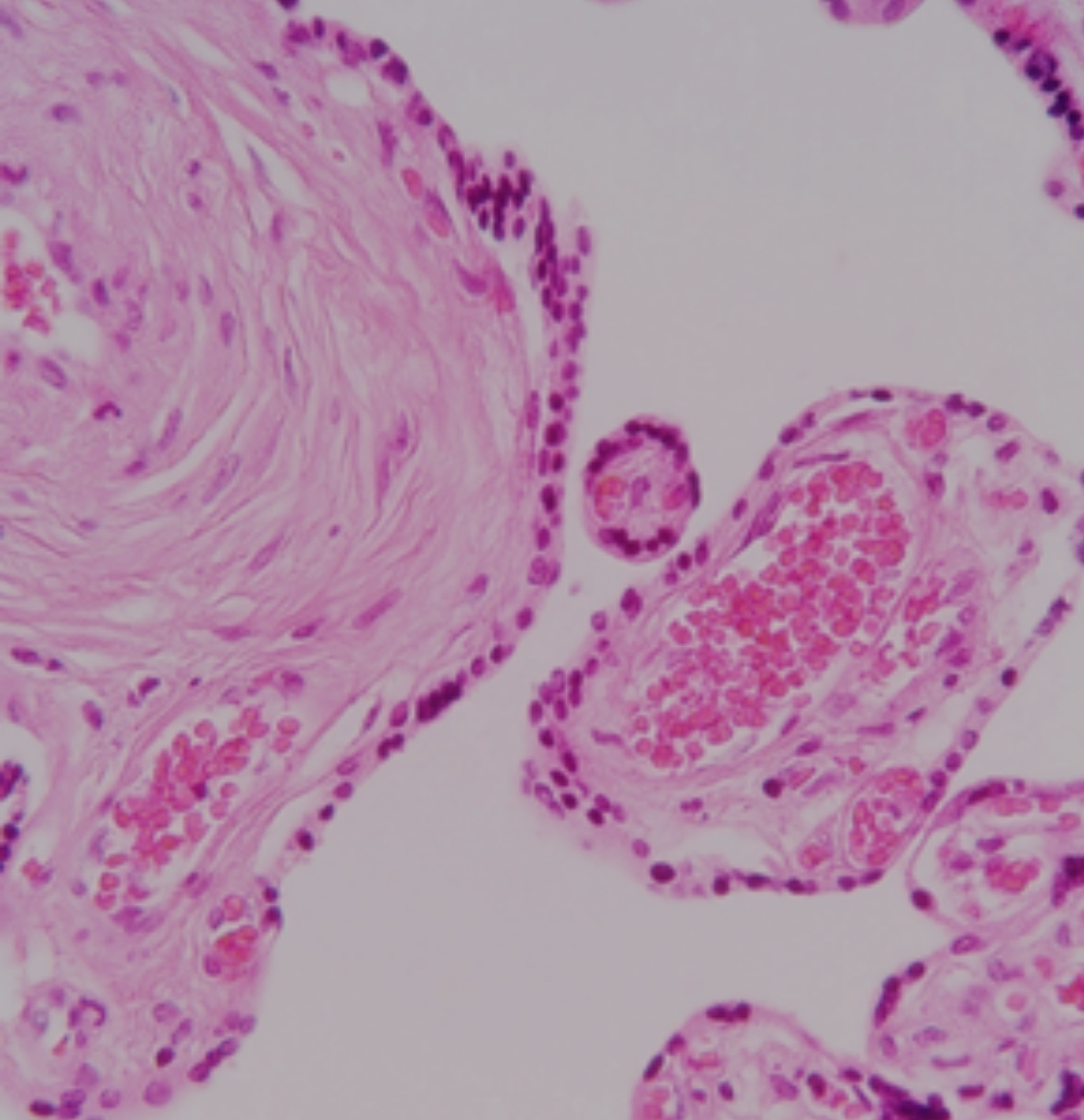

Supplement: Supplementary Figure S1 — Light micrographs of non-infected placental explants stained with Kinyoun at baseline (4 h). [file Data_Sheet_1.zip › Supplementary figures/HE S58-S69, S74/Figure S59.jpg]

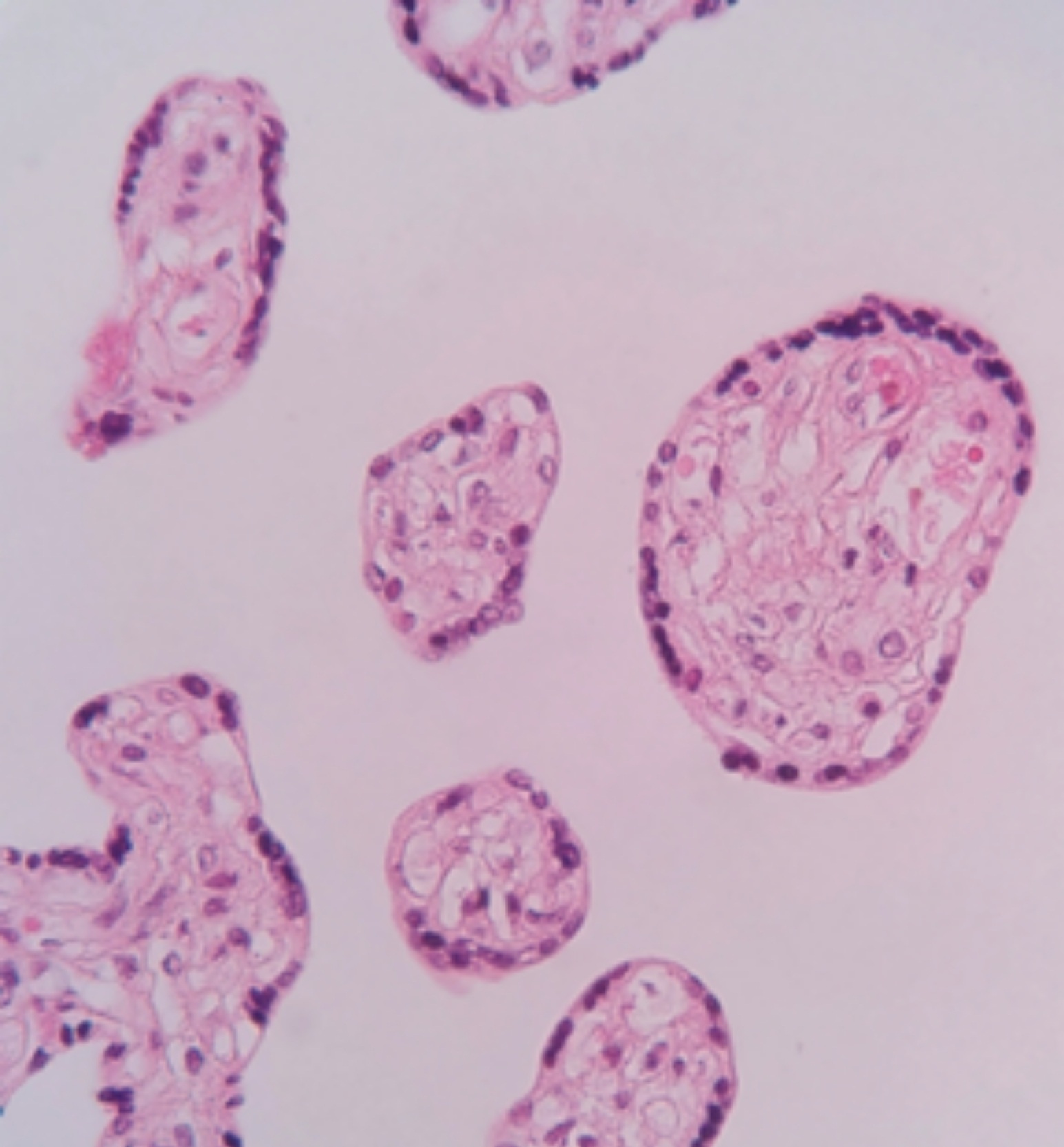

Supplement: Supplementary Figure S1 — Light micrographs of non-infected placental explants stained with Kinyoun at baseline (4 h). [file Data_Sheet_1.zip › Supplementary figures/HE S58-S69, S74/Figure S60.jpg]

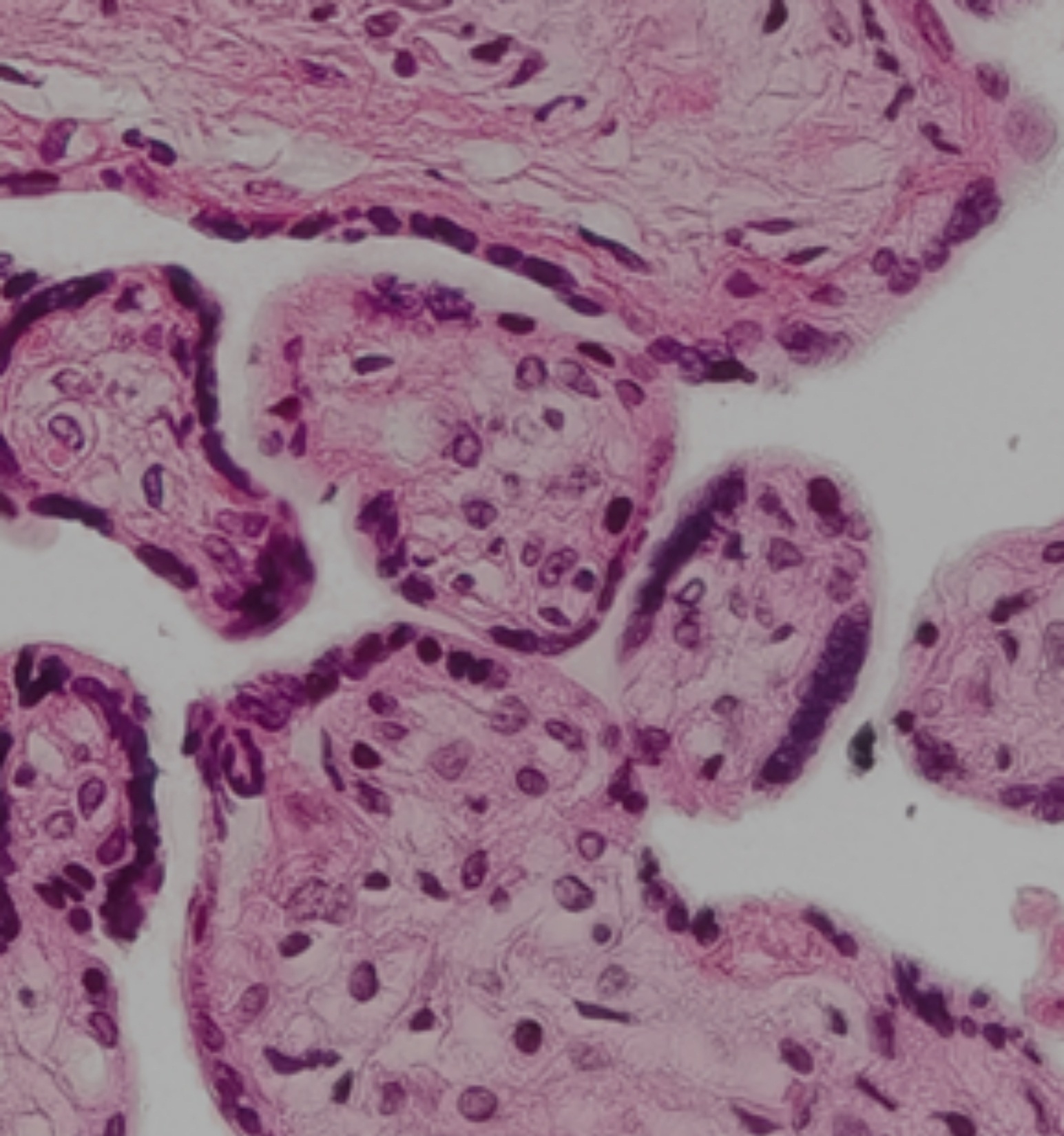

Supplement: Supplementary Figure S1 — Light micrographs of non-infected placental explants stained with Kinyoun at baseline (4 h). [file Data_Sheet_1.zip › Supplementary figures/HE S58-S69, S74/Figure S61.jpg]

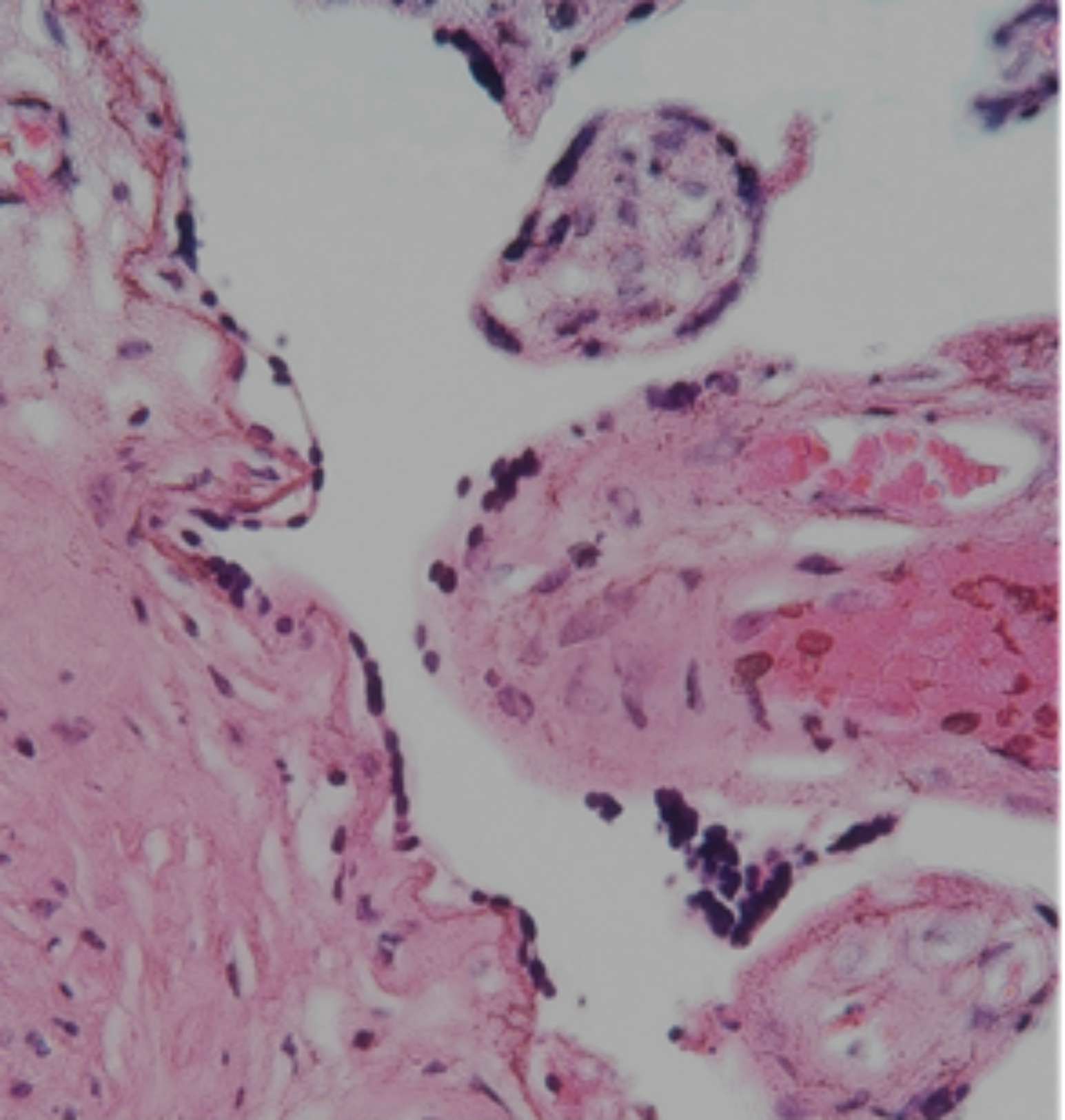

Supplement: Supplementary Figure S1 — Light micrographs of non-infected placental explants stained with Kinyoun at baseline (4 h). [file Data_Sheet_1.zip › Supplementary figures/HE S58-S69, S74/Figure S62.jpg]

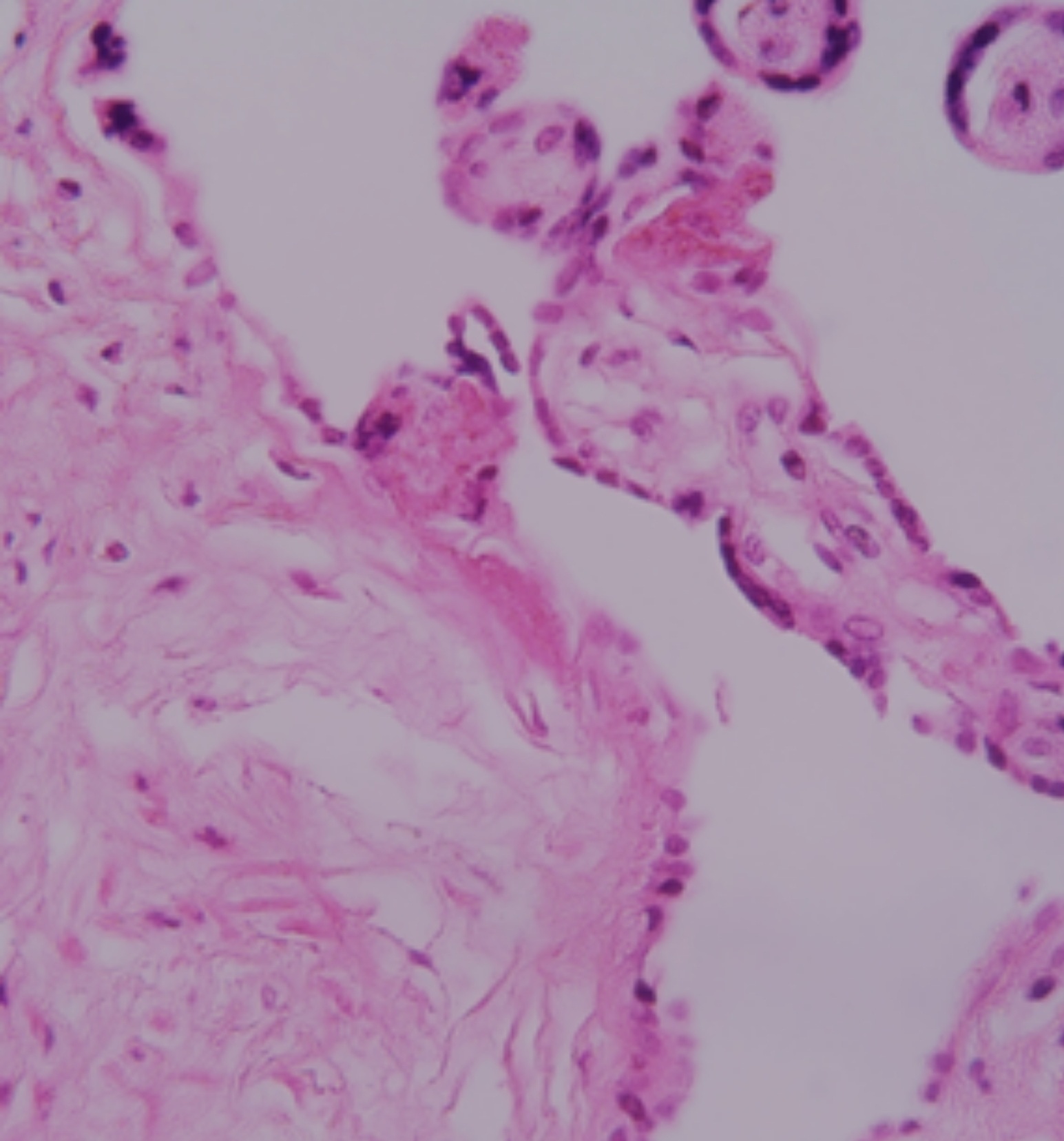

Supplement: Supplementary Figure S1 — Light micrographs of non-infected placental explants stained with Kinyoun at baseline (4 h). [file Data_Sheet_1.zip › Supplementary figures/HE S58-S69, S74/Figure S63.jpg]

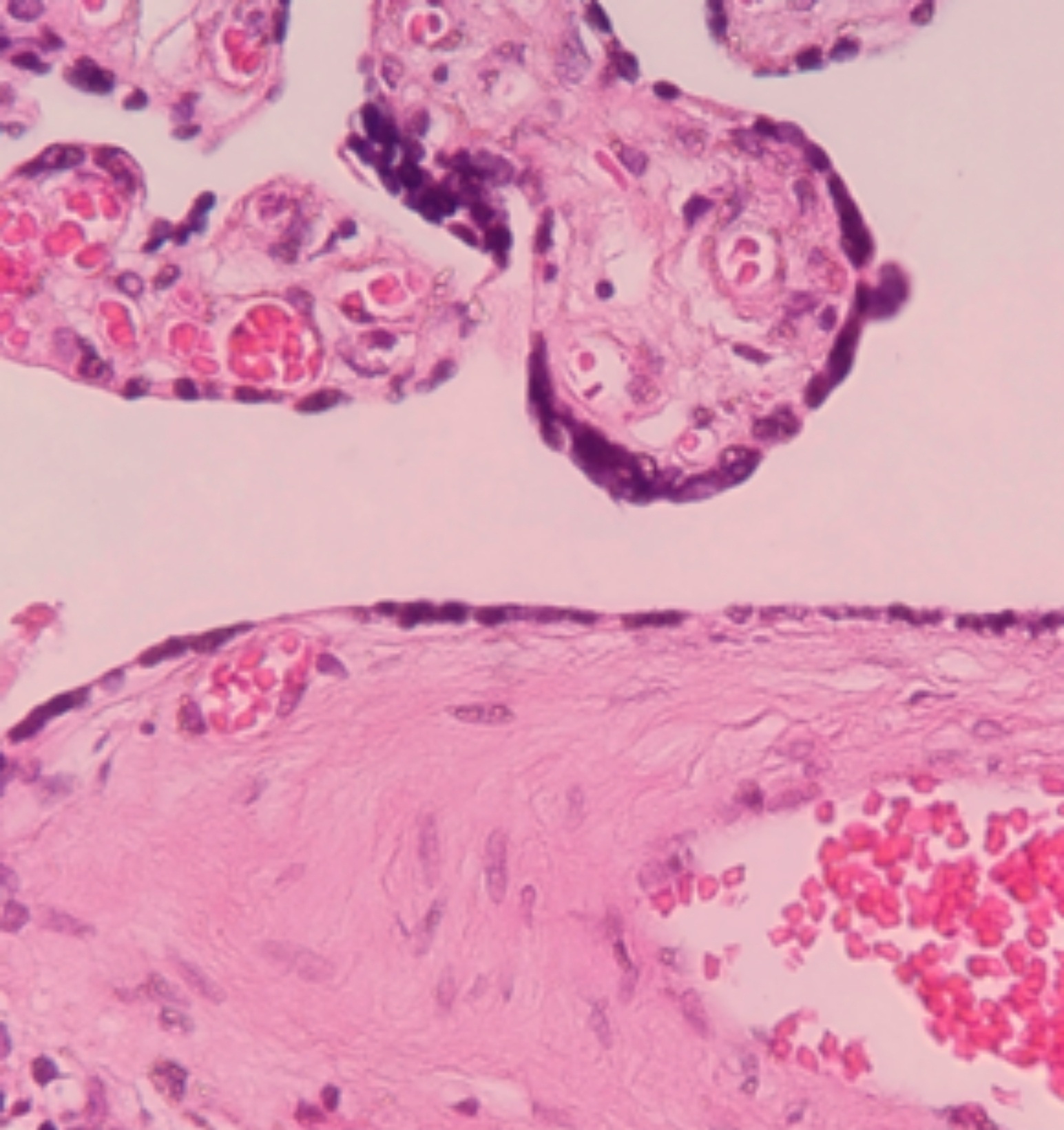

Supplement: Supplementary Figure S1 — Light micrographs of non-infected placental explants stained with Kinyoun at baseline (4 h). [file Data_Sheet_1.zip › Supplementary figures/HE S58-S69, S74/Figure S64.jpg]

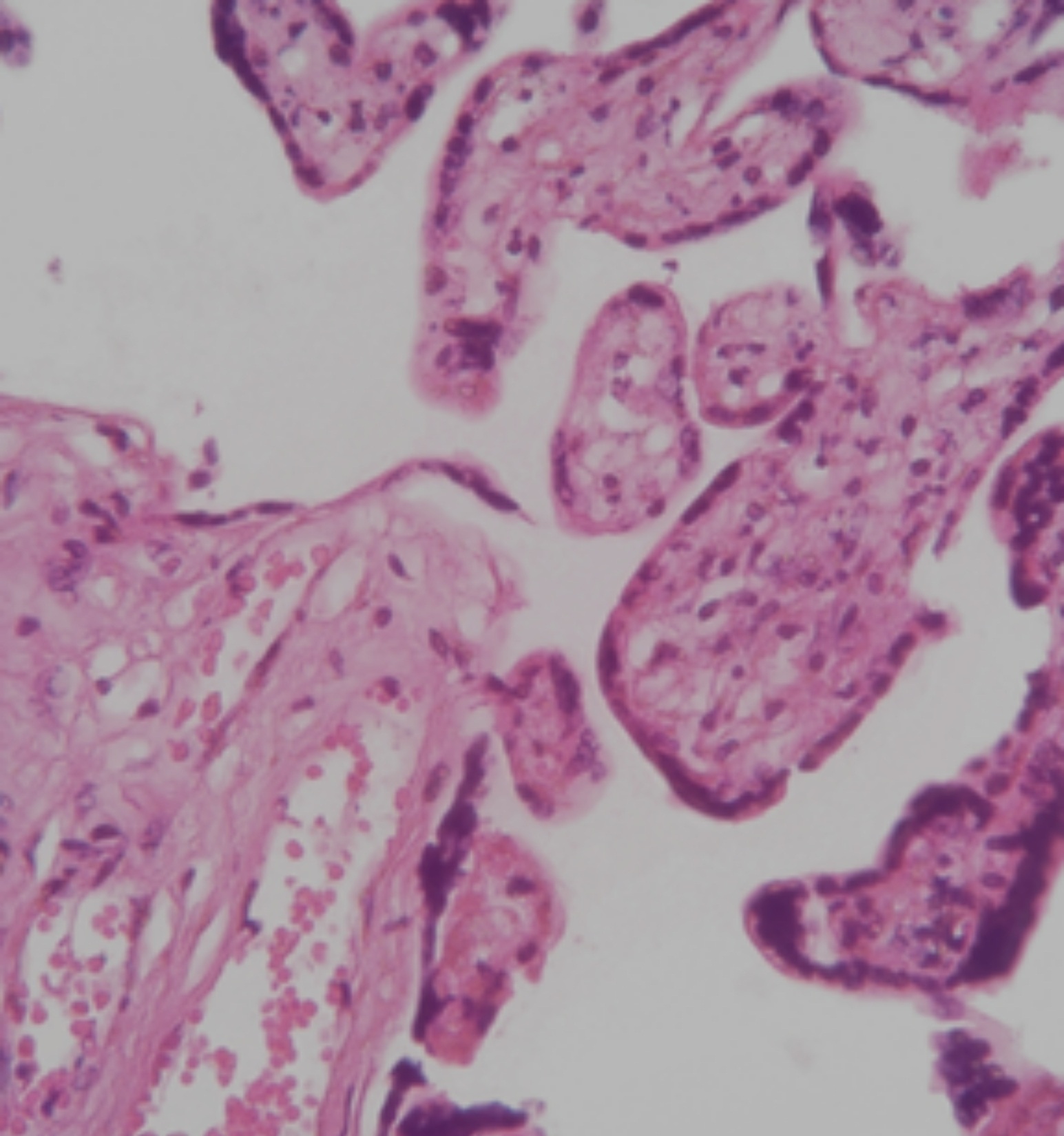

Supplement: Supplementary Figure S1 — Light micrographs of non-infected placental explants stained with Kinyoun at baseline (4 h). [file Data_Sheet_1.zip › Supplementary figures/HE S58-S69, S74/Figure S65.jpg]

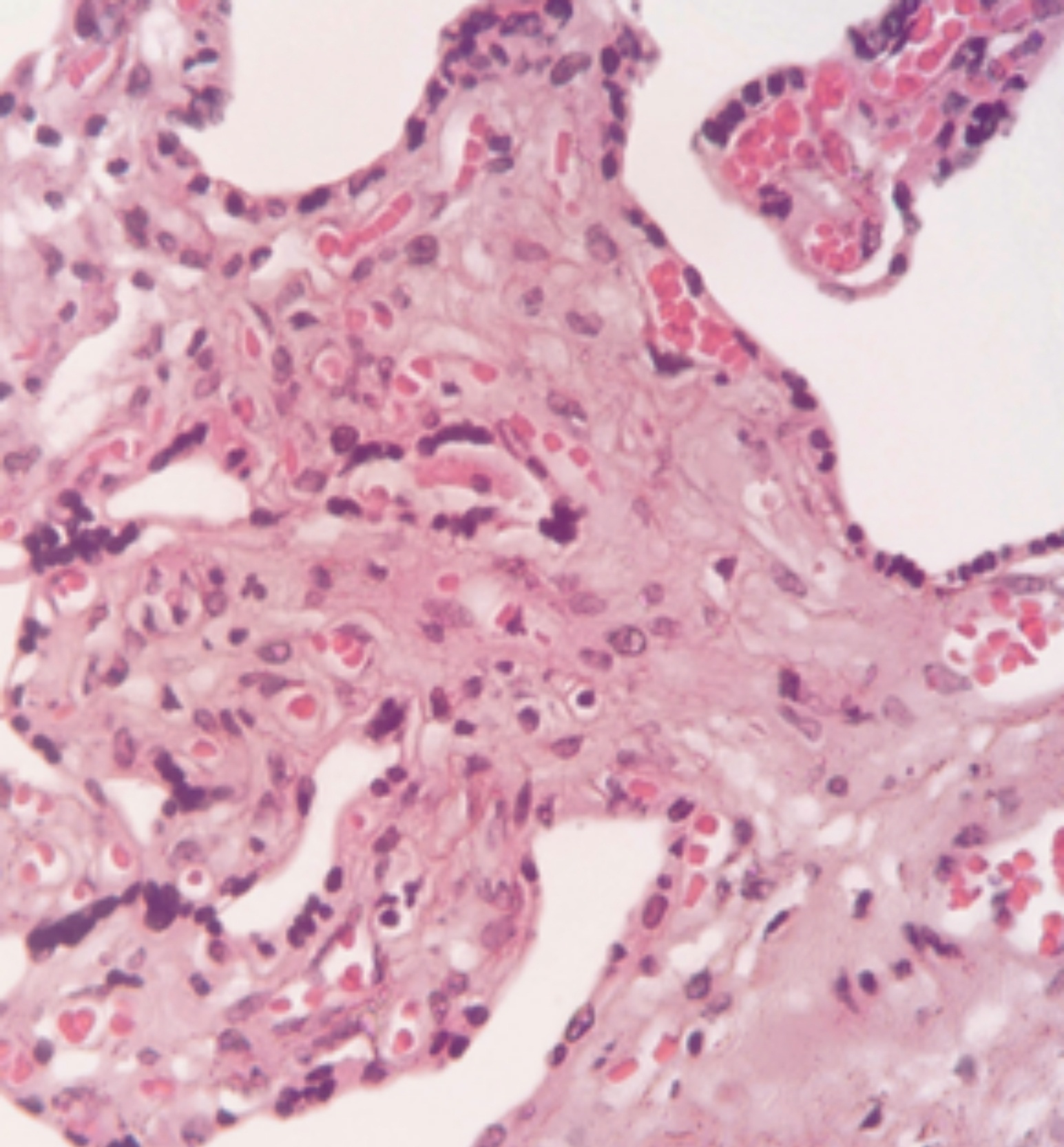

Supplement: Supplementary Figure S1 — Light micrographs of non-infected placental explants stained with Kinyoun at baseline (4 h). [file Data_Sheet_1.zip › Supplementary figures/HE S58-S69, S74/Figure S66.jpg]

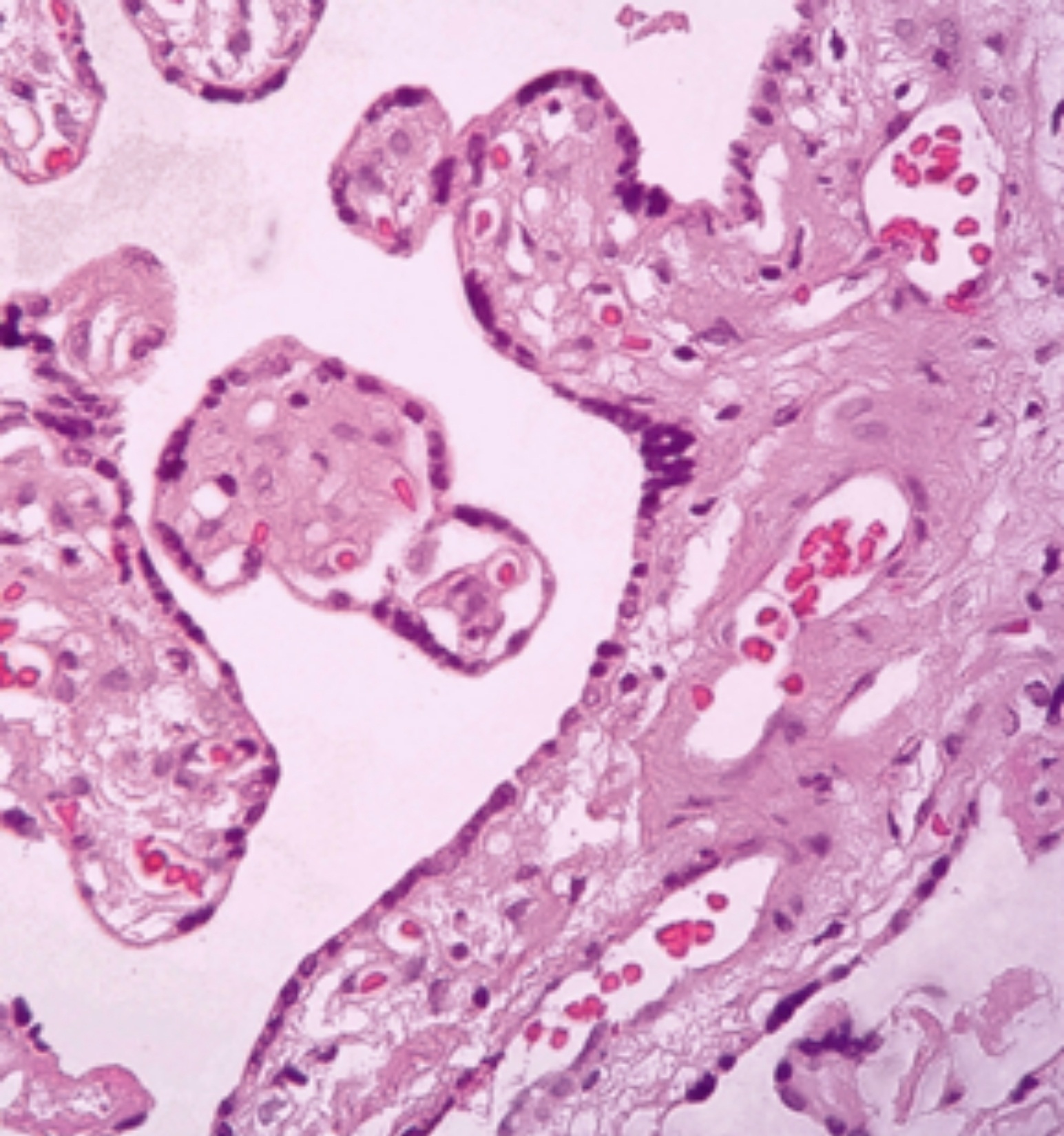

Supplement: Supplementary Figure S1 — Light micrographs of non-infected placental explants stained with Kinyoun at baseline (4 h). [file Data_Sheet_1.zip › Supplementary figures/HE S58-S69, S74/Figure S67.jpg]

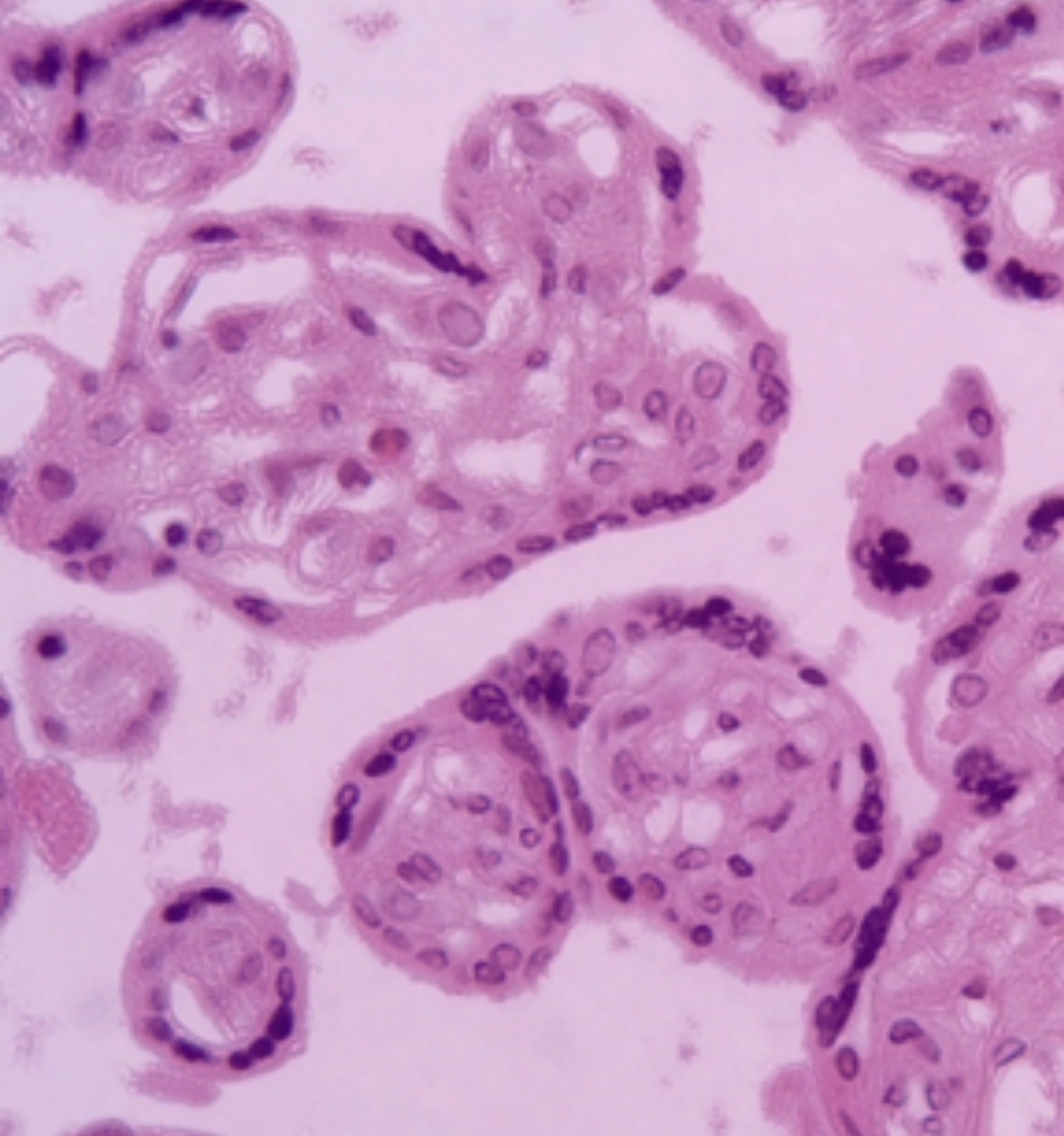

Supplement: Supplementary Figure S1 — Light micrographs of non-infected placental explants stained with Kinyoun at baseline (4 h). [file Data_Sheet_1.zip › Supplementary figures/HE S58-S69, S74/Figure S68.jpg]

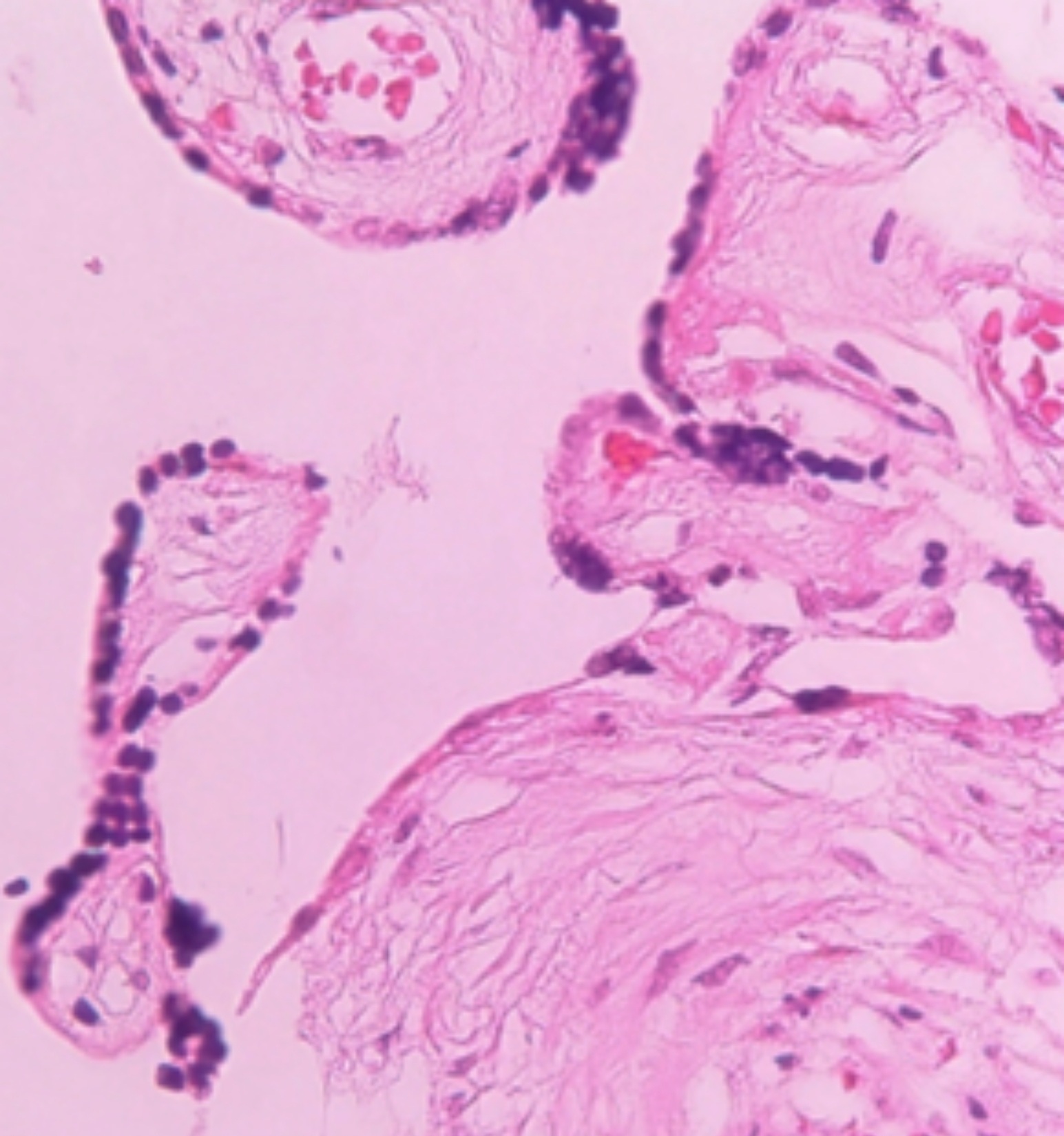

Supplement: Supplementary Figure S1 — Light micrographs of non-infected placental explants stained with Kinyoun at baseline (4 h). [file Data_Sheet_1.zip › Supplementary figures/HE S58-S69, S74/Figure S69.jpg]

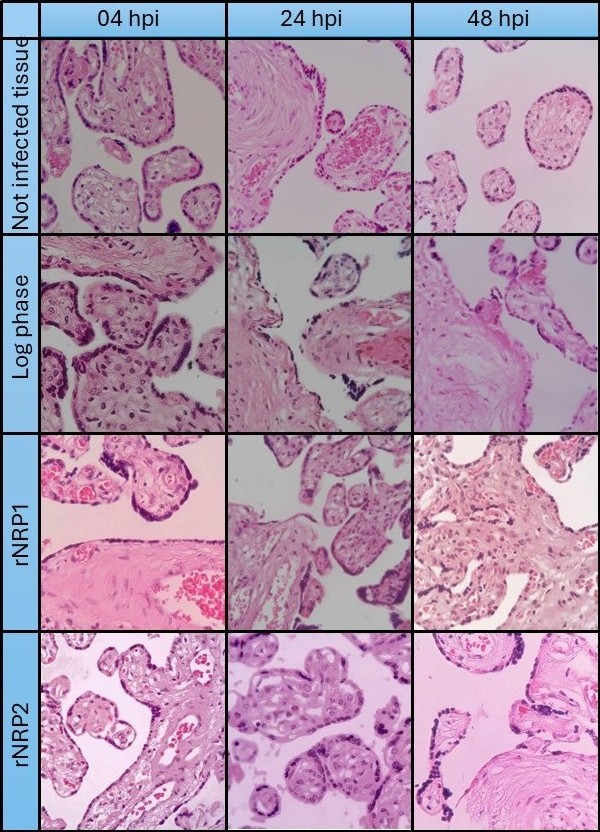

Supplement: Supplementary Figure S1 — Light micrographs of non-infected placental explants stained with Kinyoun at baseline (4 h). [file Data_Sheet_1.zip › Supplementary figures/HE S58-S69, S74/Figure S74.jpg]

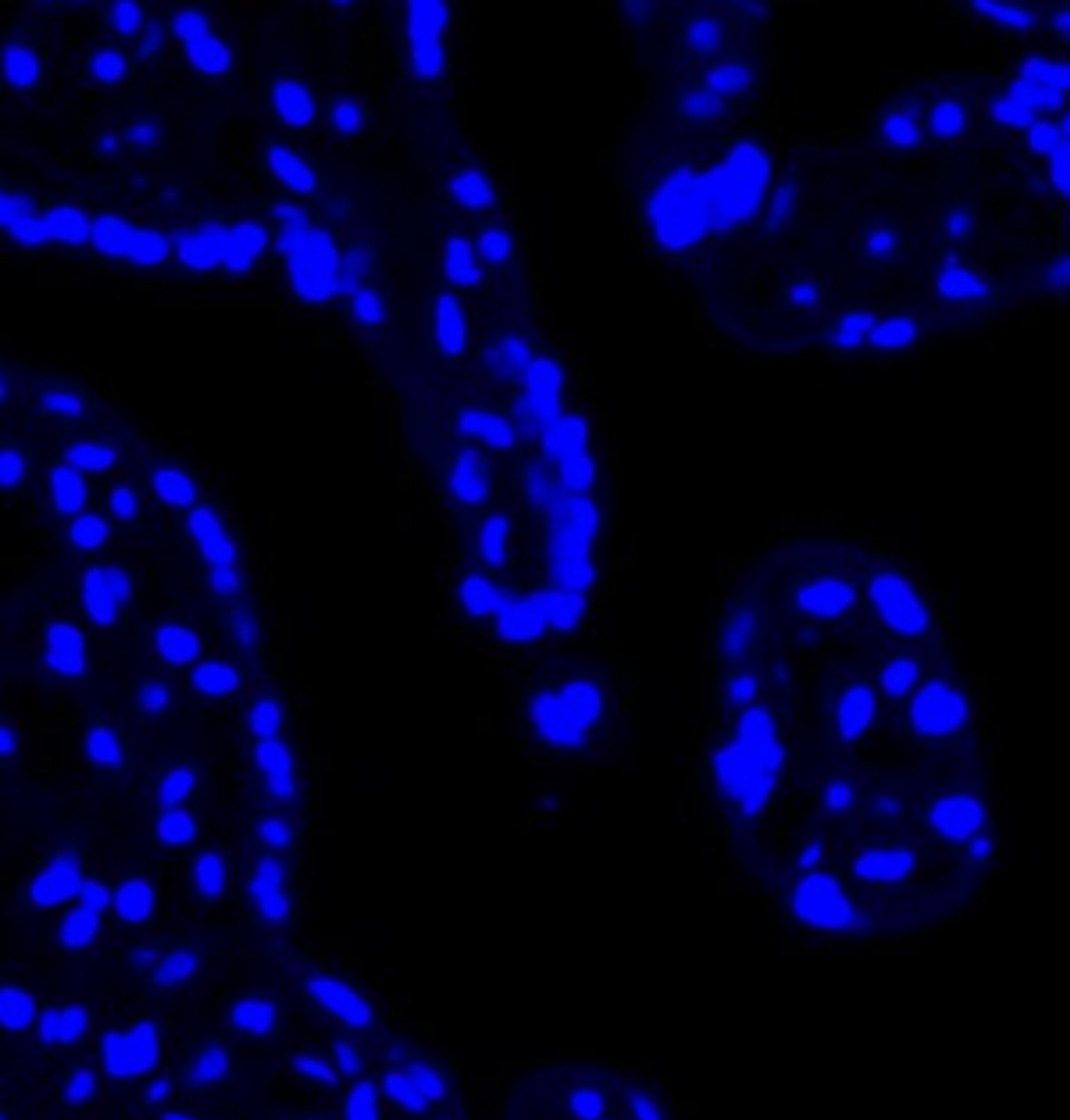

Supplement: Supplementary Figure S1 — Light micrographs of non-infected placental explants stained with Kinyoun at baseline (4 h). [file Data_Sheet_1.zip › Supplementary figures/Immunofluorescence S13-S57, S71-S73/Figure S13.jpg]

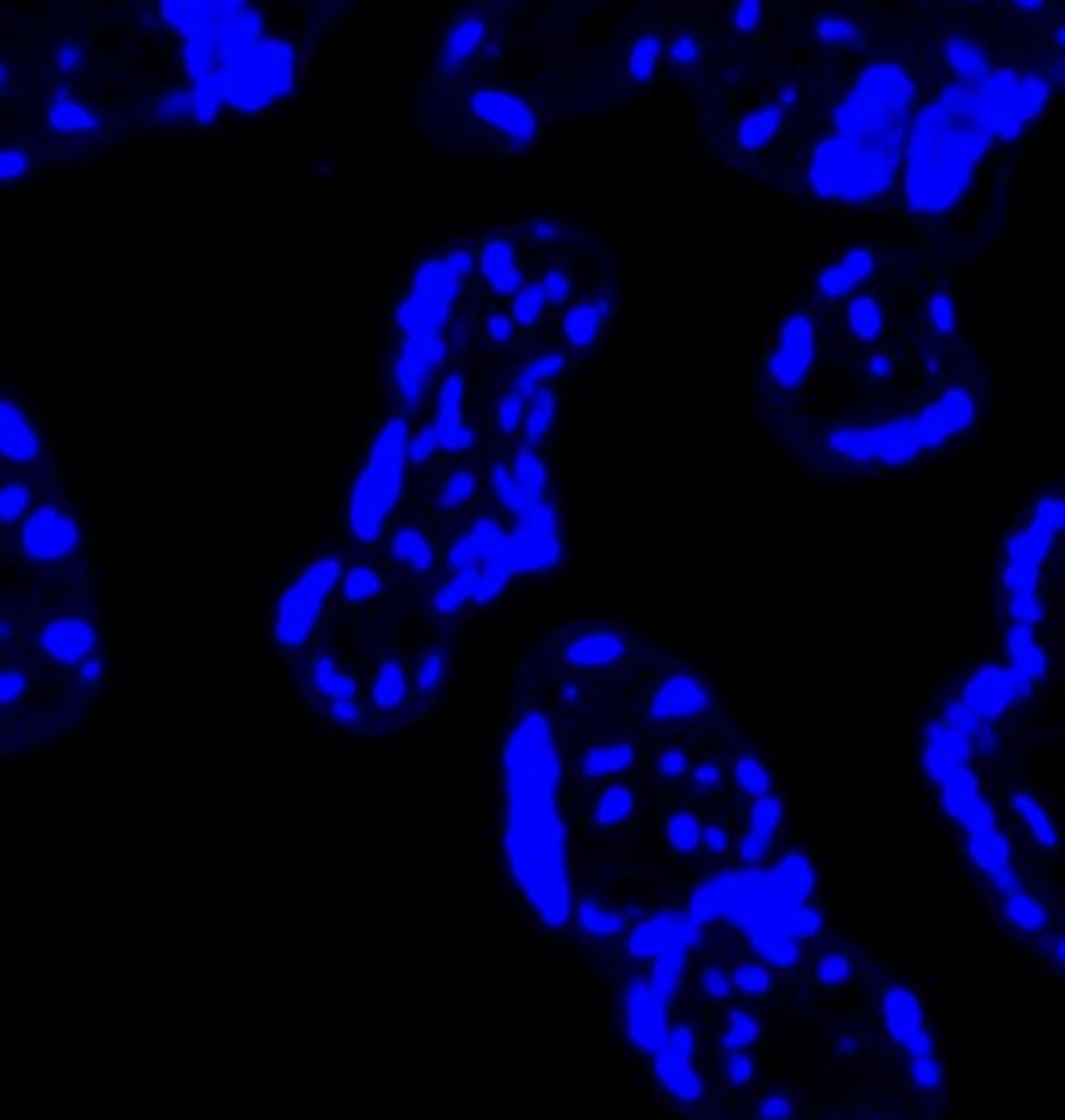

Supplement: Supplementary Figure S1 — Light micrographs of non-infected placental explants stained with Kinyoun at baseline (4 h). [file Data_Sheet_1.zip › Supplementary figures/Immunofluorescence S13-S57, S71-S73/Figure S14.jpg]

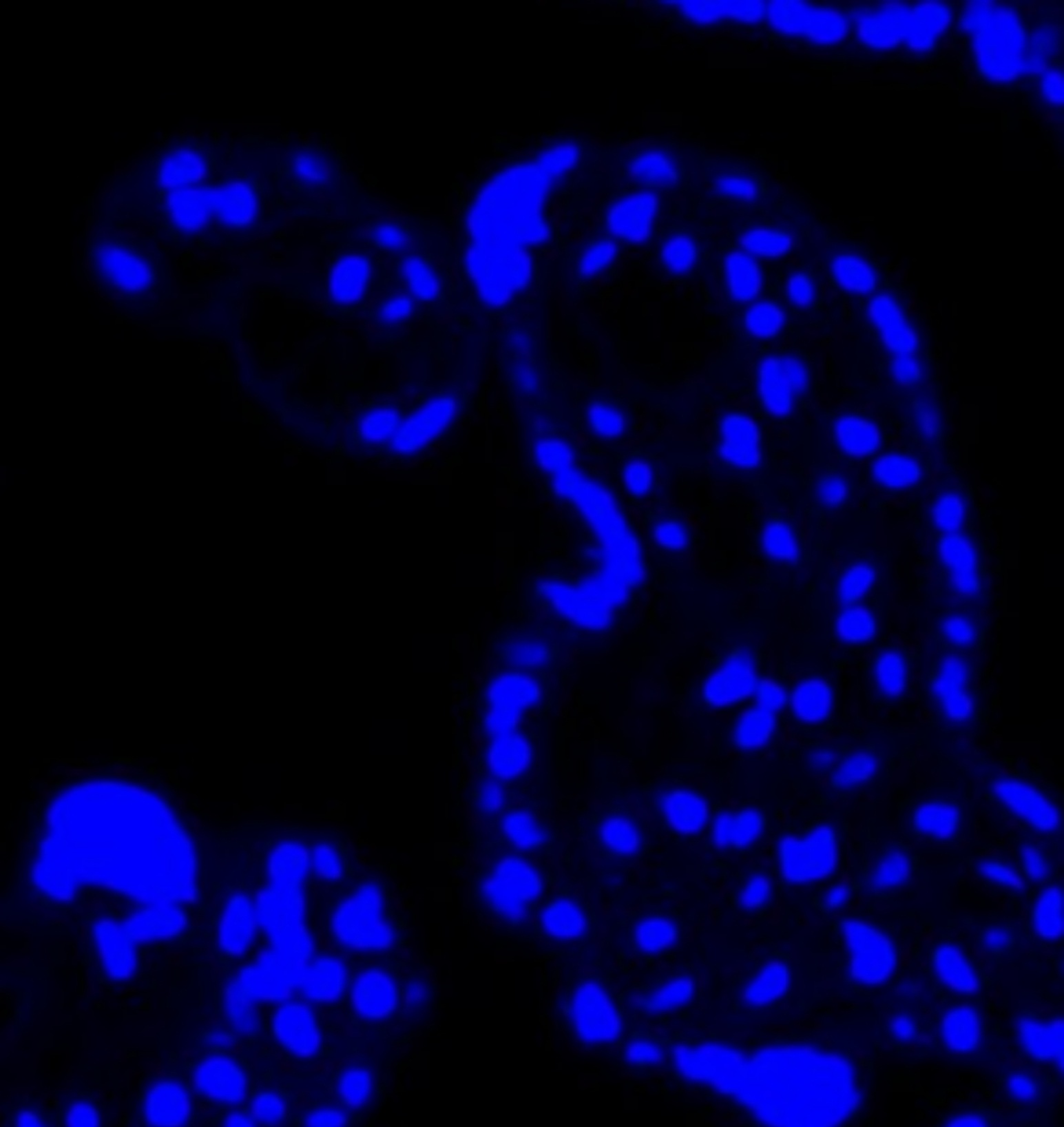

Supplement: Supplementary Figure S1 — Light micrographs of non-infected placental explants stained with Kinyoun at baseline (4 h). [file Data_Sheet_1.zip › Supplementary figures/Immunofluorescence S13-S57, S71-S73/Figure S15.jpg]

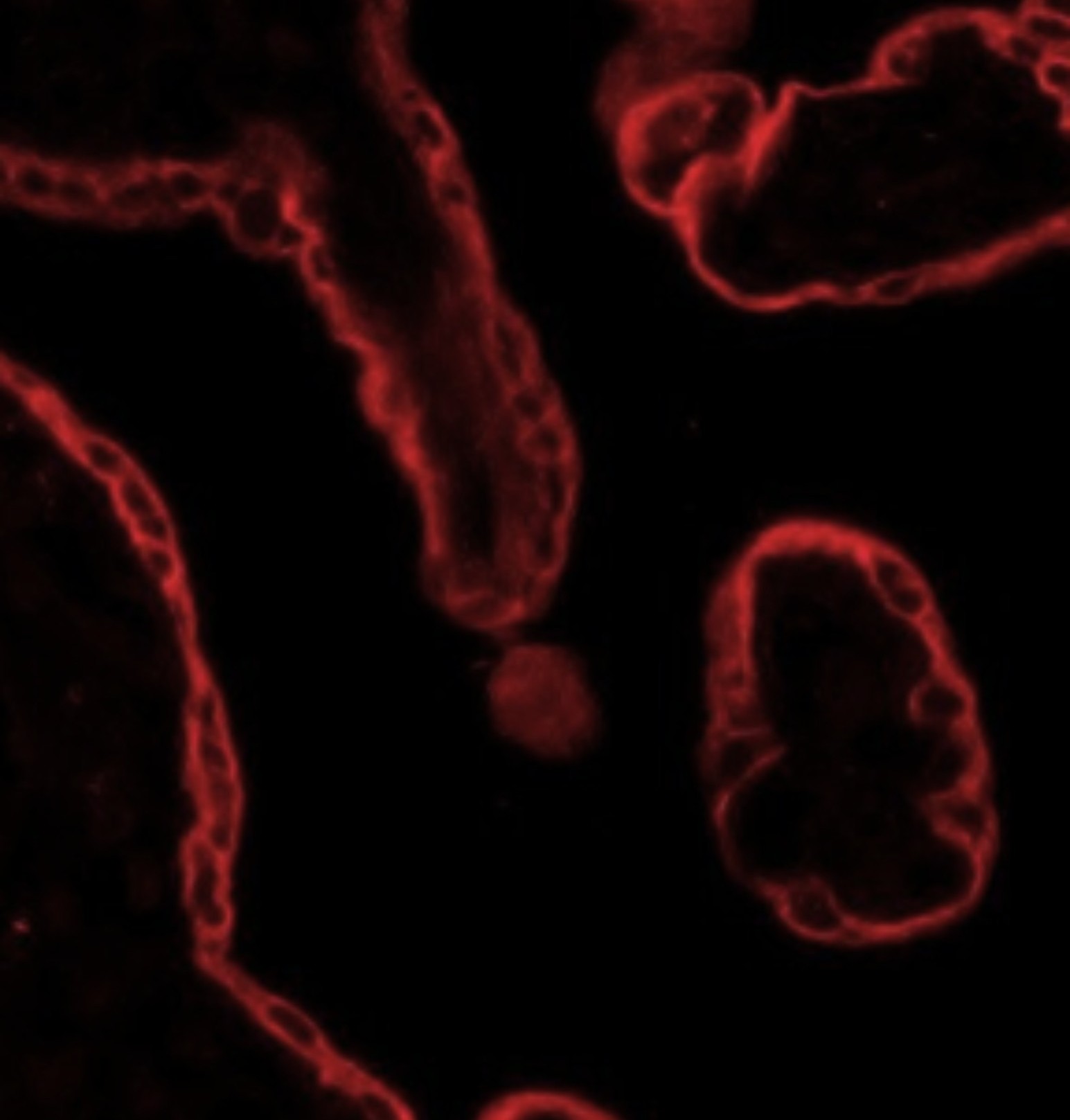

Supplement: Supplementary Figure S1 — Light micrographs of non-infected placental explants stained with Kinyoun at baseline (4 h). [file Data_Sheet_1.zip › Supplementary figures/Immunofluorescence S13-S57, S71-S73/Figure S16.jpg]

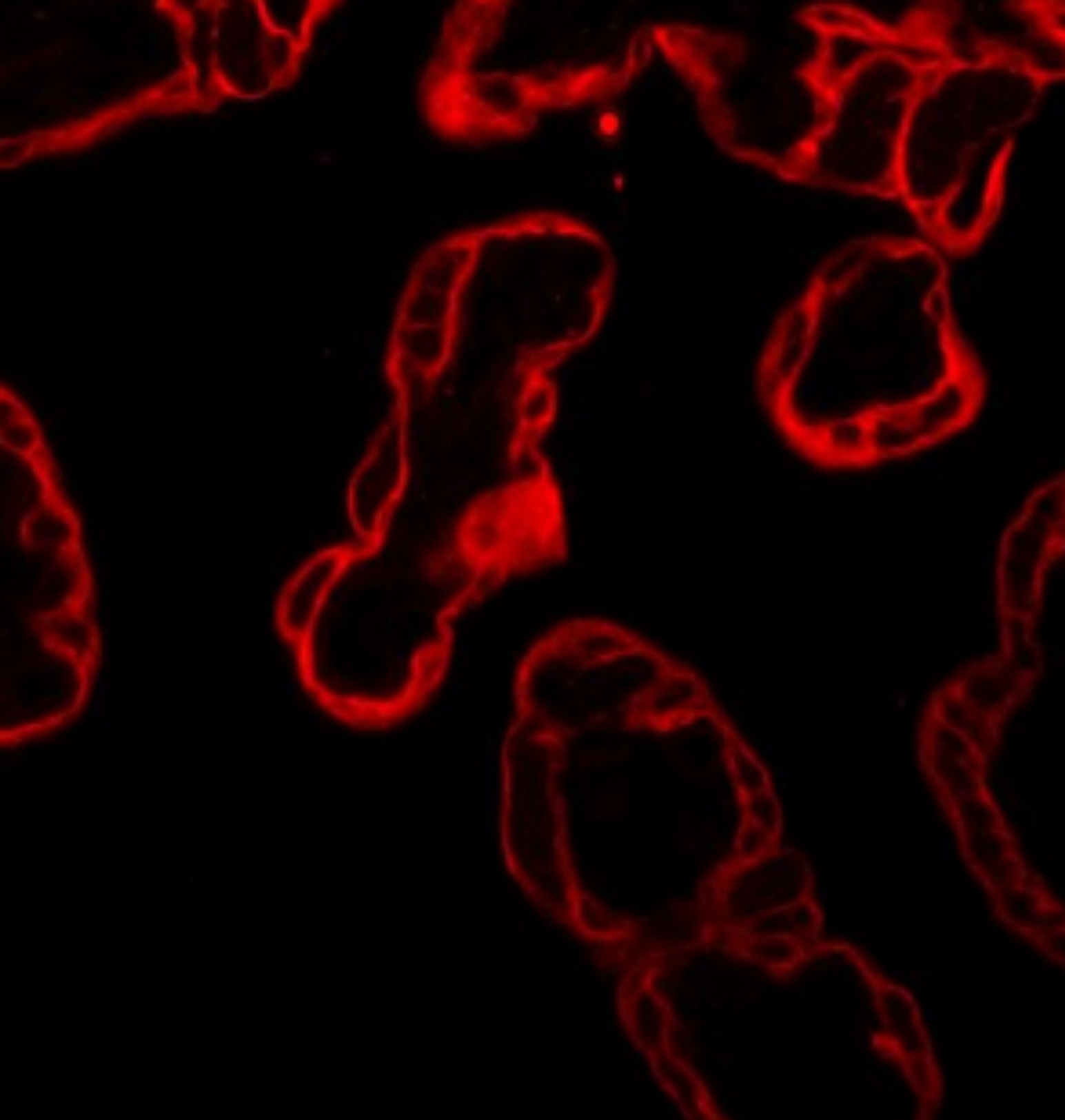

Supplement: Supplementary Figure S1 — Light micrographs of non-infected placental explants stained with Kinyoun at baseline (4 h). [file Data_Sheet_1.zip › Supplementary figures/Immunofluorescence S13-S57, S71-S73/Figure S17.jpg]

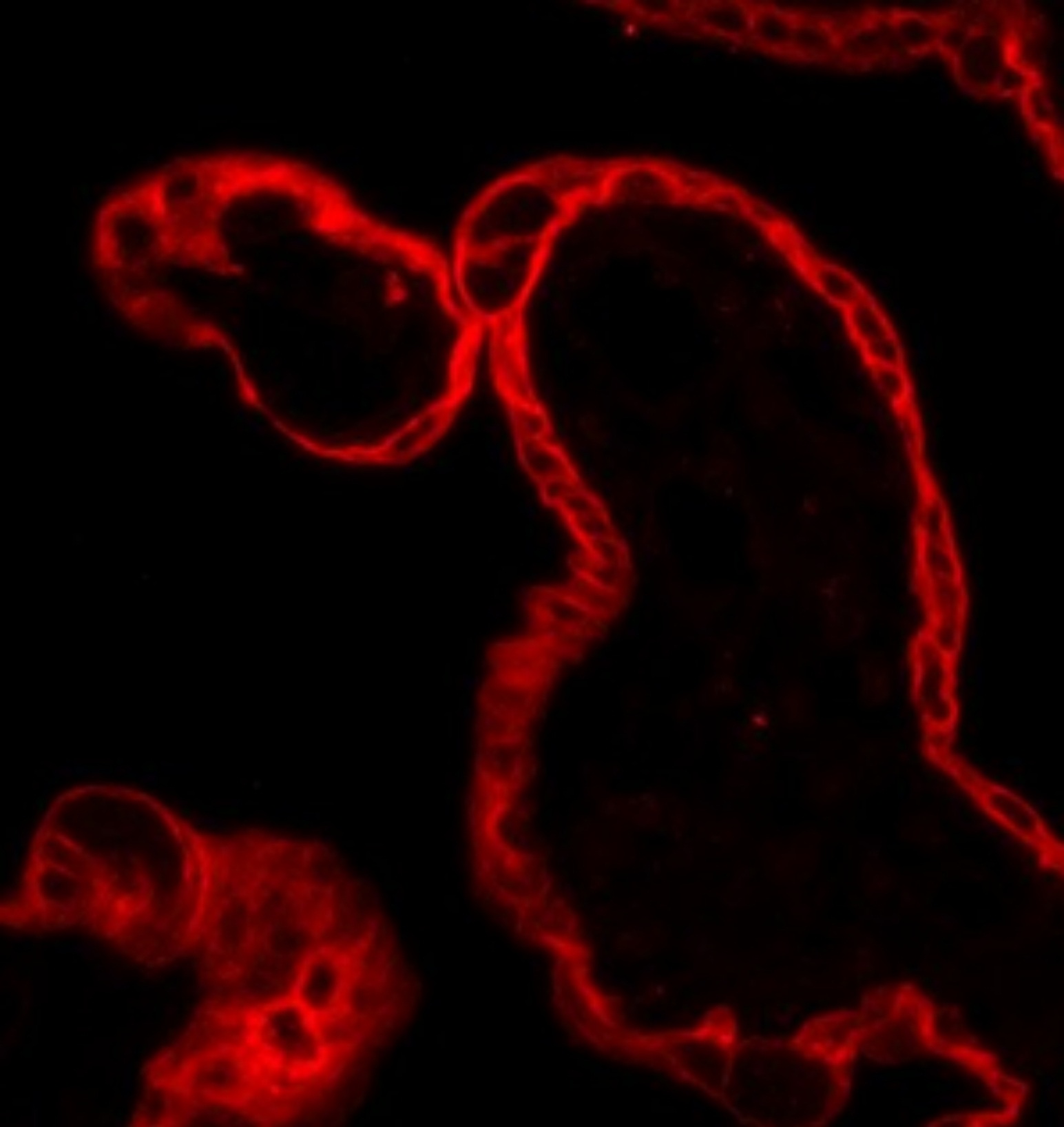

Supplement: Supplementary Figure S1 — Light micrographs of non-infected placental explants stained with Kinyoun at baseline (4 h). [file Data_Sheet_1.zip › Supplementary figures/Immunofluorescence S13-S57, S71-S73/Figure S18.jpg]

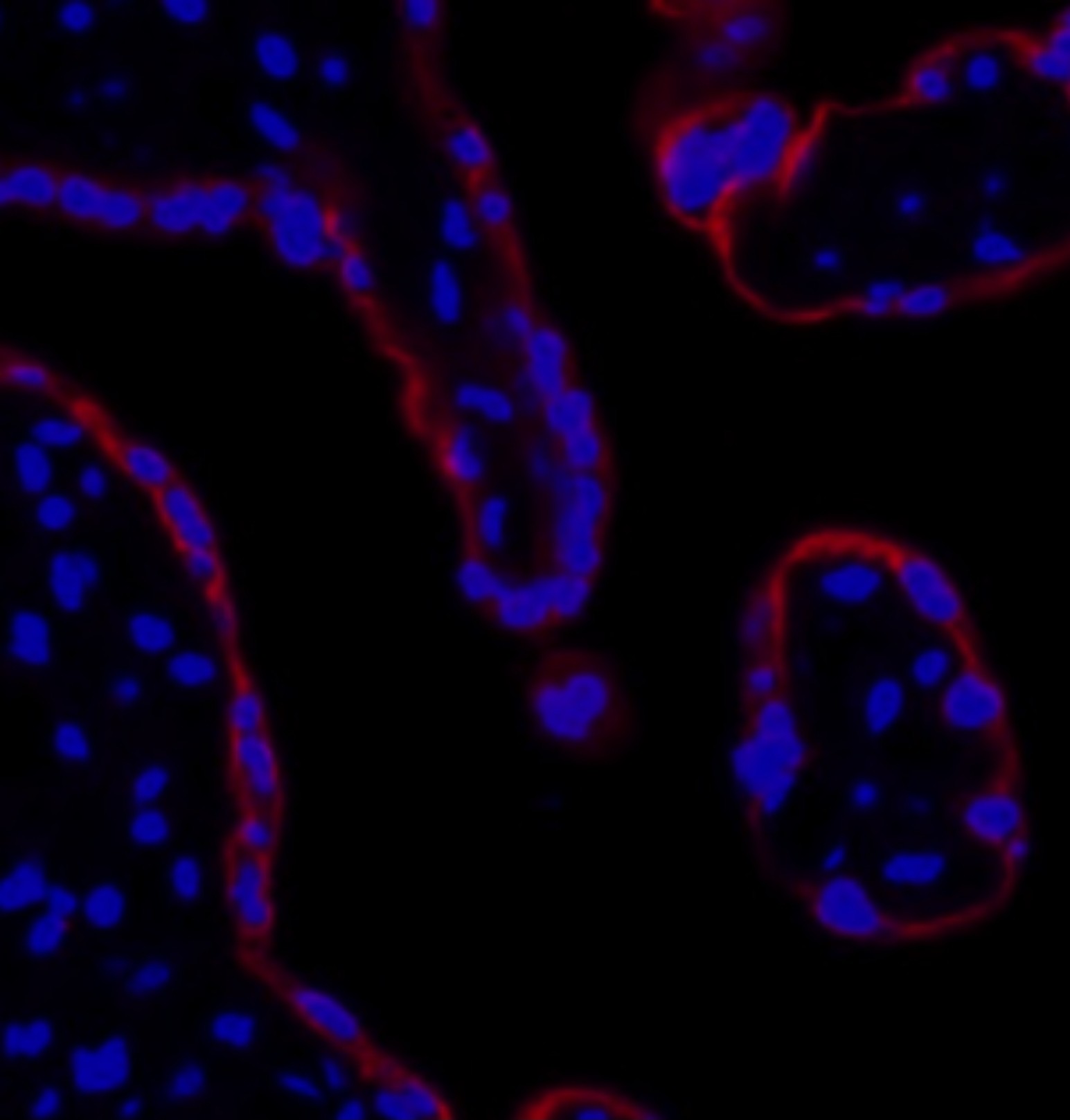

Supplement: Supplementary Figure S1 — Light micrographs of non-infected placental explants stained with Kinyoun at baseline (4 h). [file Data_Sheet_1.zip › Supplementary figures/Immunofluorescence S13-S57, S71-S73/Figure S19.jpg]

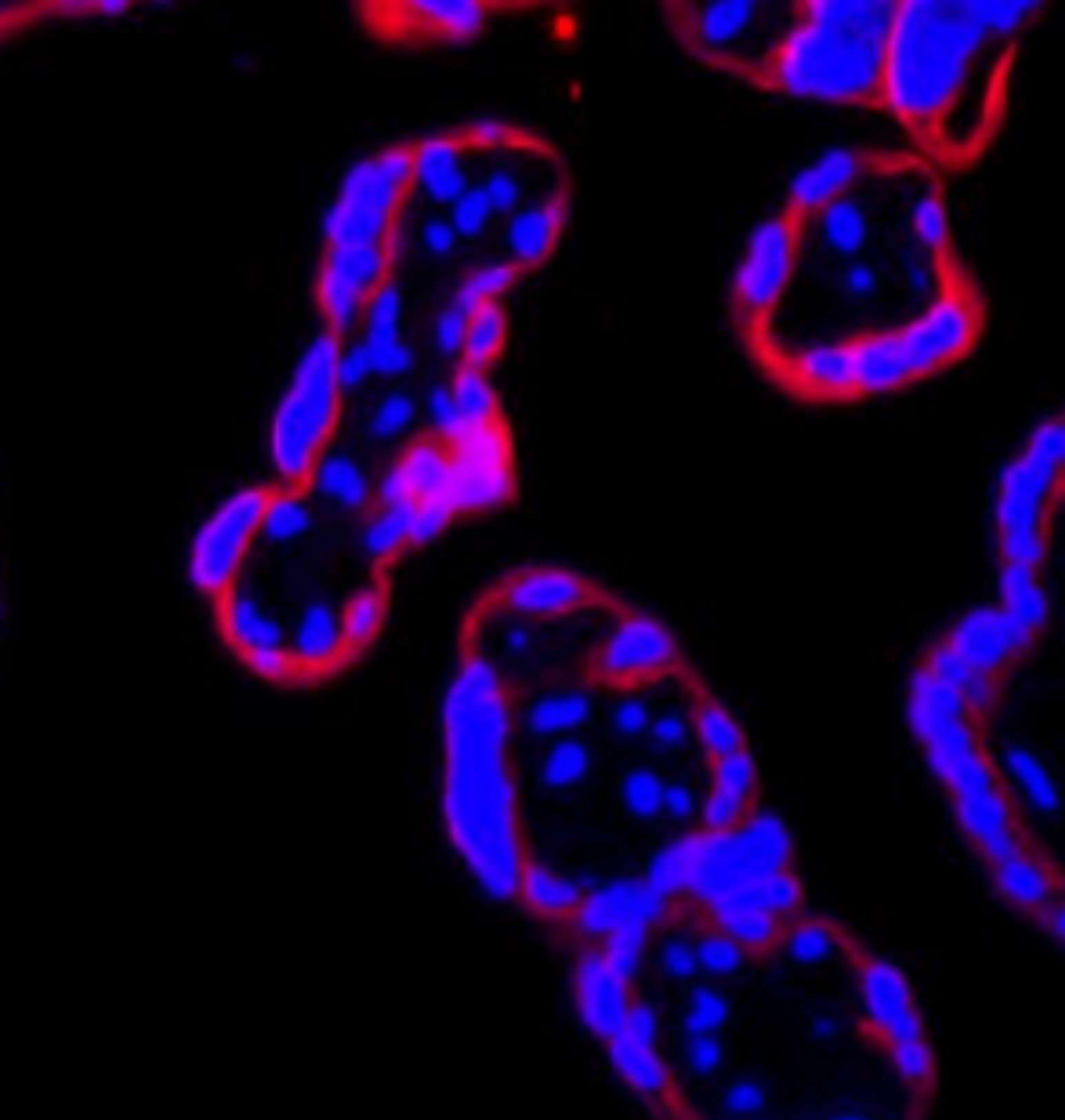

Supplement: Supplementary Figure S1 — Light micrographs of non-infected placental explants stained with Kinyoun at baseline (4 h). [file Data_Sheet_1.zip › Supplementary figures/Immunofluorescence S13-S57, S71-S73/Figure S20.jpg]

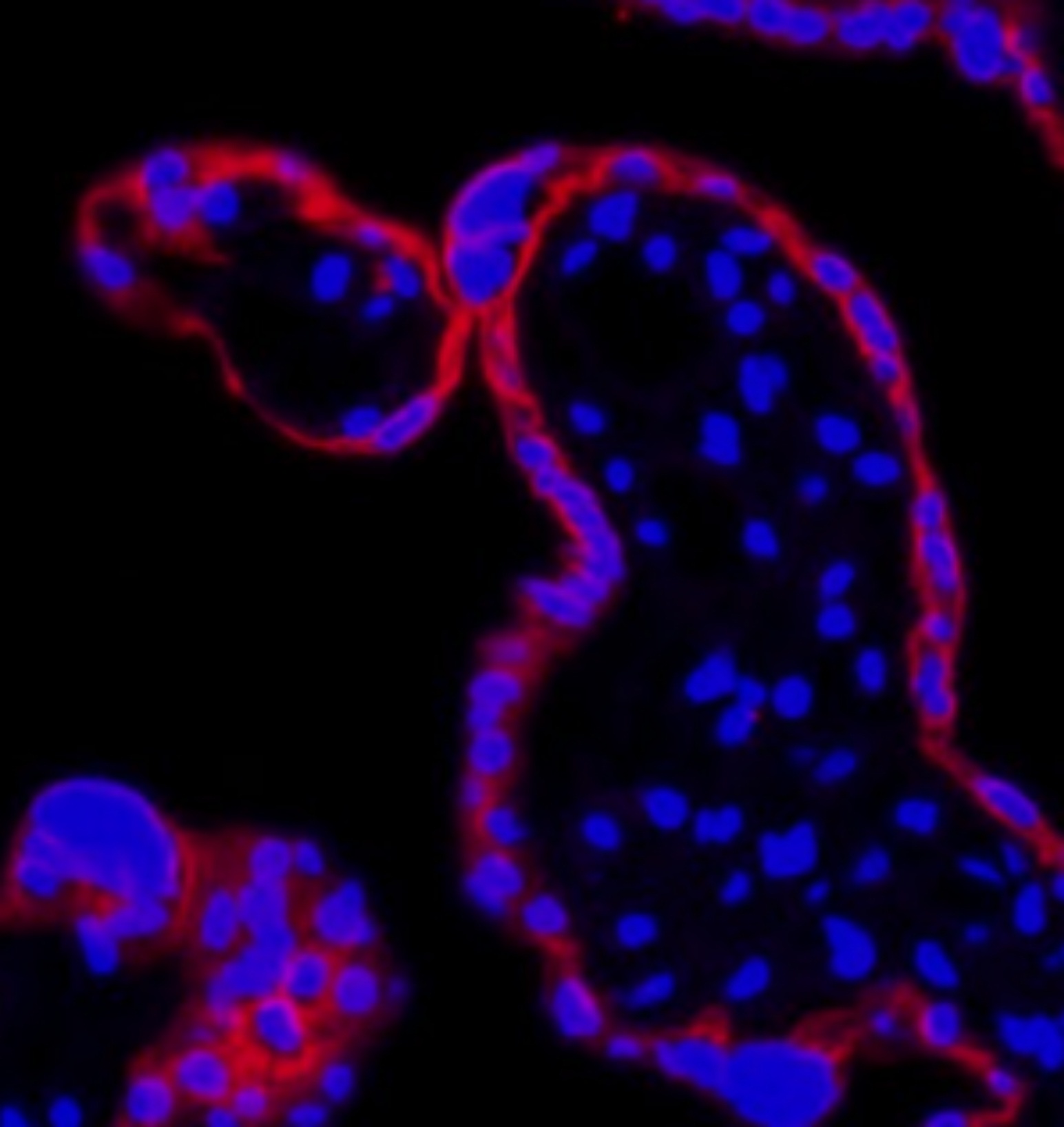

Supplement: Supplementary Figure S1 — Light micrographs of non-infected placental explants stained with Kinyoun at baseline (4 h). [file Data_Sheet_1.zip › Supplementary figures/Immunofluorescence S13-S57, S71-S73/Figure S21.jpg]

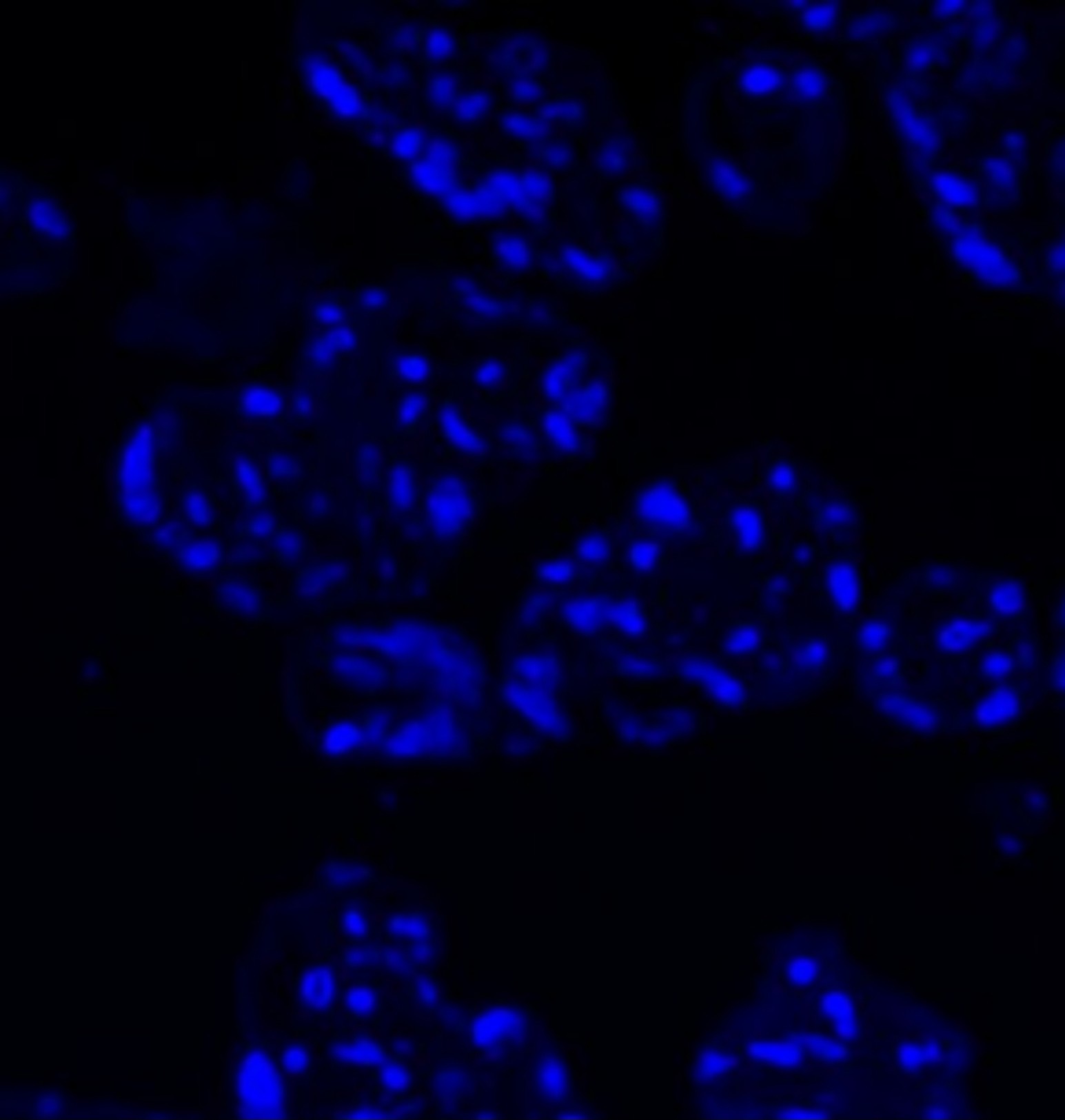

Supplement: Supplementary Figure S1 — Light micrographs of non-infected placental explants stained with Kinyoun at baseline (4 h). [file Data_Sheet_1.zip › Supplementary figures/Immunofluorescence S13-S57, S71-S73/Figure S22.jpg]

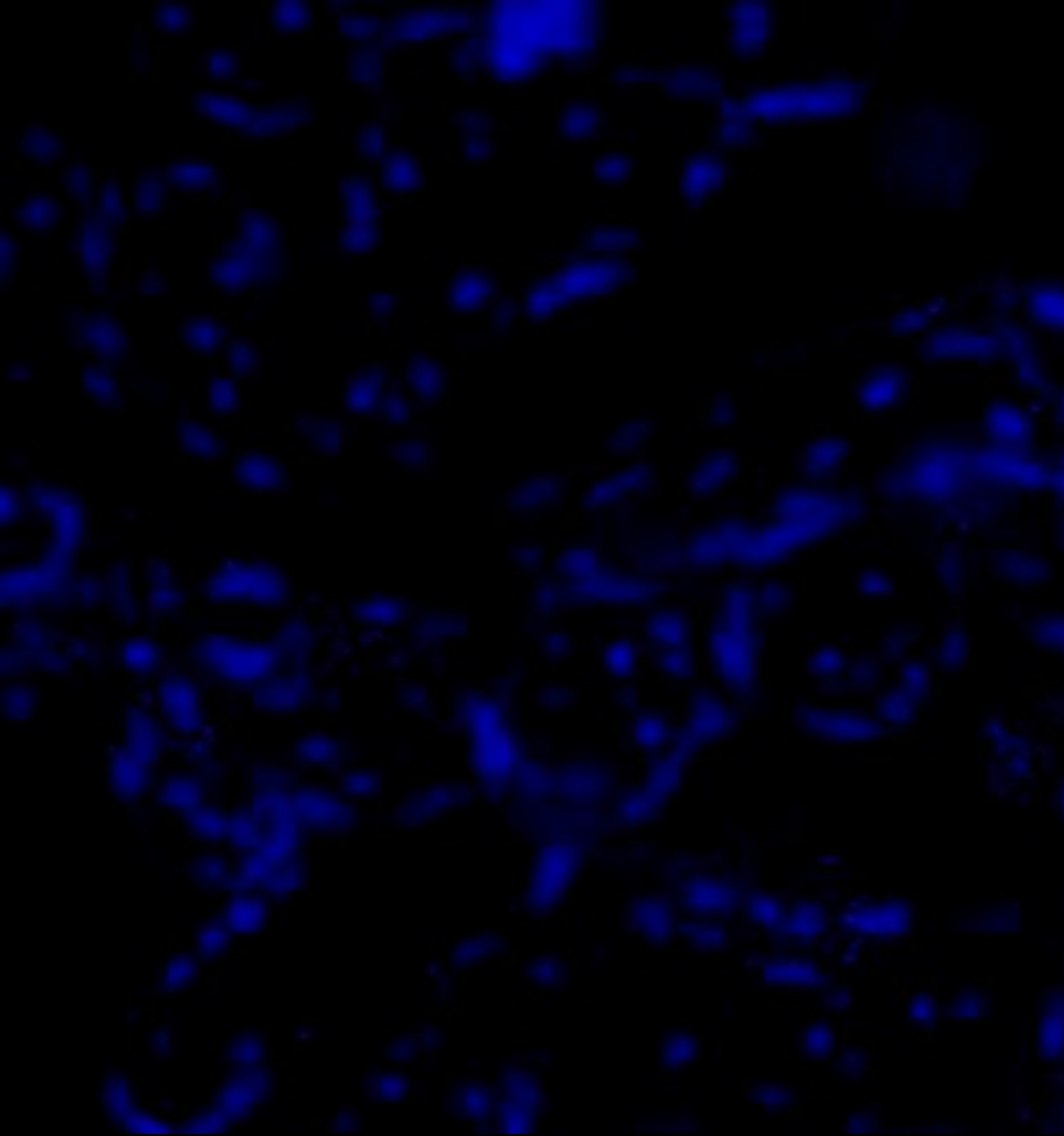

Supplement: Supplementary Figure S1 — Light micrographs of non-infected placental explants stained with Kinyoun at baseline (4 h). [file Data_Sheet_1.zip › Supplementary figures/Immunofluorescence S13-S57, S71-S73/Figure S23.jpg]

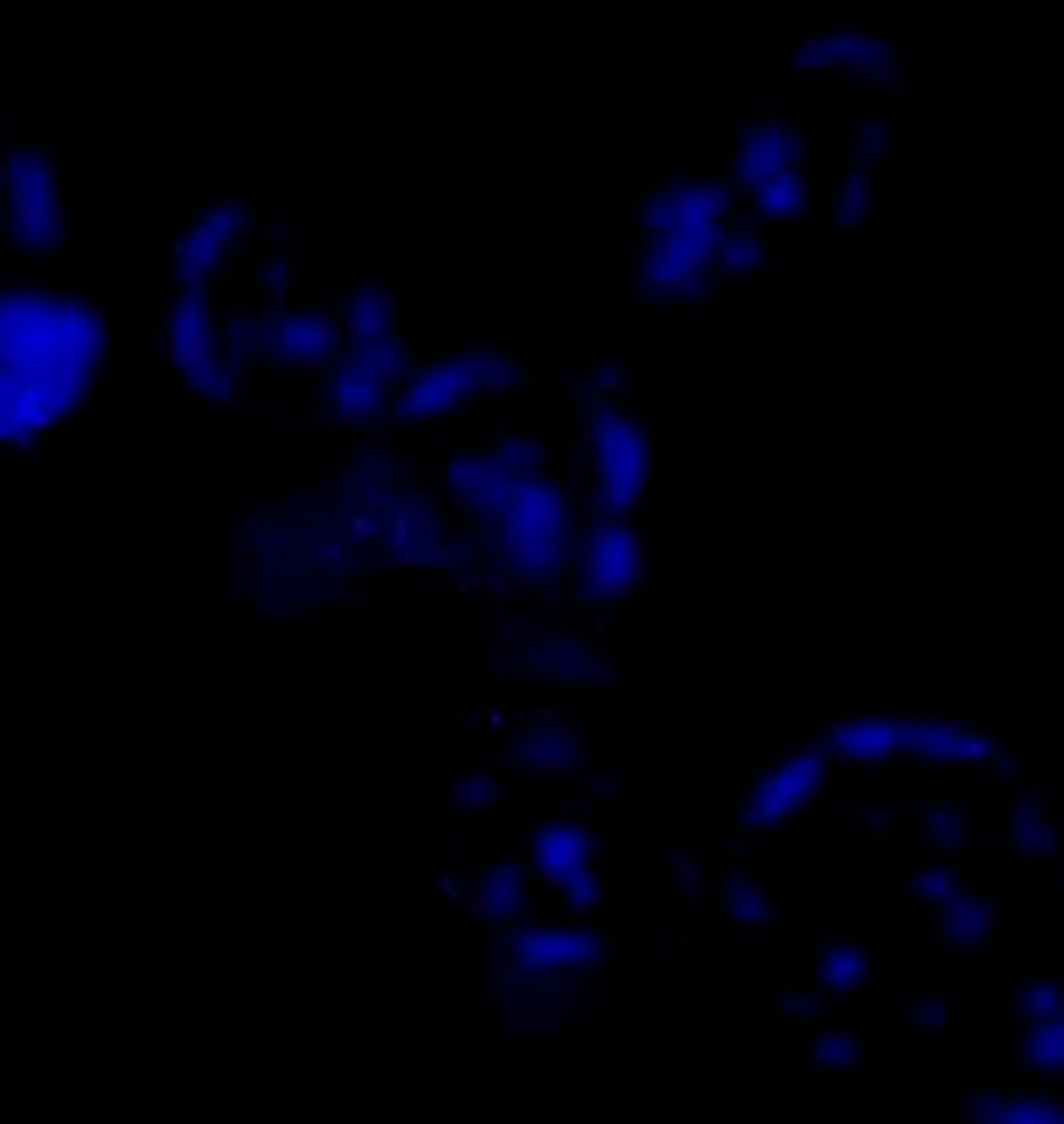

Supplement: Supplementary Figure S1 — Light micrographs of non-infected placental explants stained with Kinyoun at baseline (4 h). [file Data_Sheet_1.zip › Supplementary figures/Immunofluorescence S13-S57, S71-S73/Figure S24.jpg]

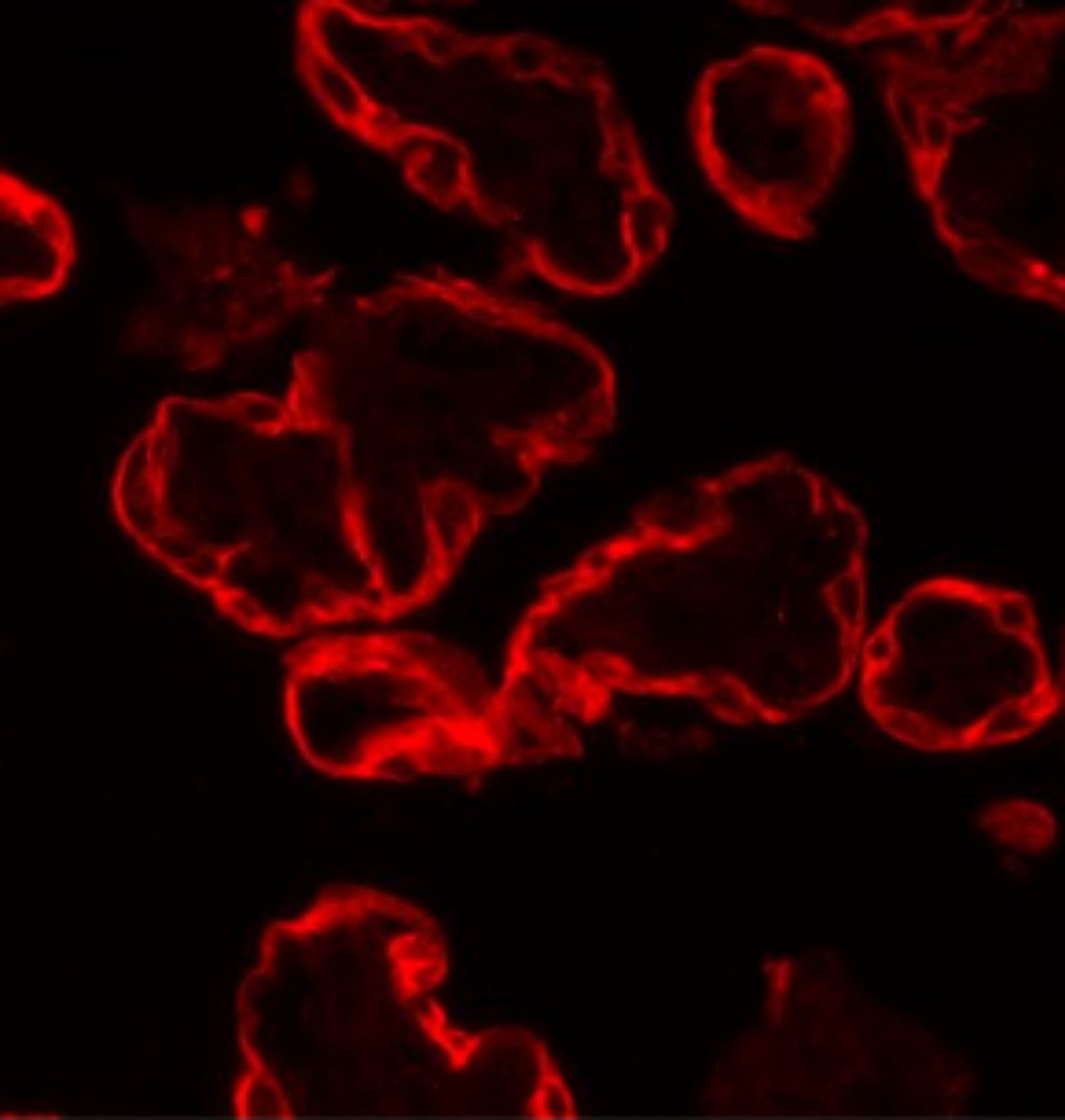

Supplement: Supplementary Figure S1 — Light micrographs of non-infected placental explants stained with Kinyoun at baseline (4 h). [file Data_Sheet_1.zip › Supplementary figures/Immunofluorescence S13-S57, S71-S73/Figure S25.jpg]

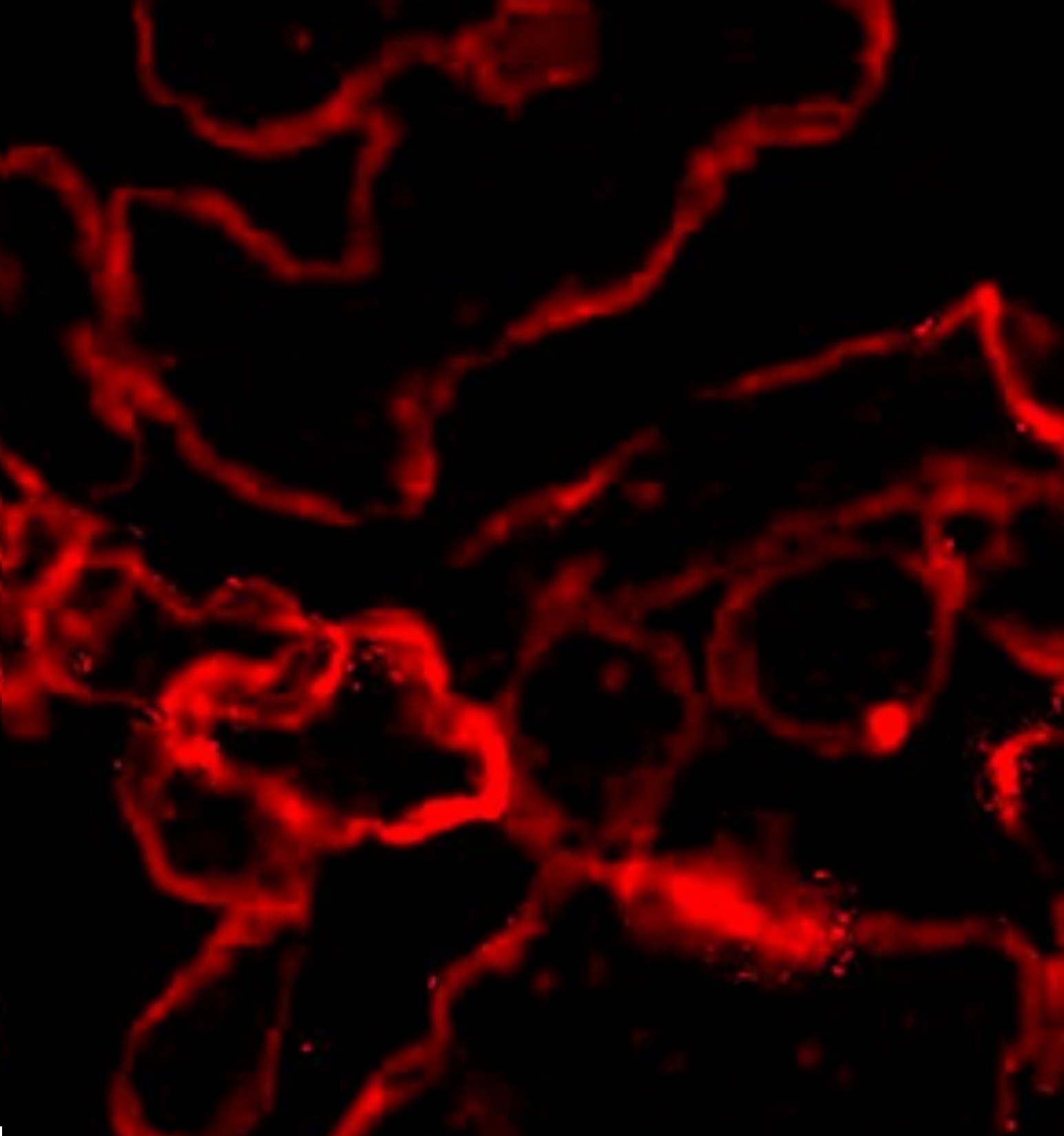

Supplement: Supplementary Figure S1 — Light micrographs of non-infected placental explants stained with Kinyoun at baseline (4 h). [file Data_Sheet_1.zip › Supplementary figures/Immunofluorescence S13-S57, S71-S73/Figure S26.jpg]

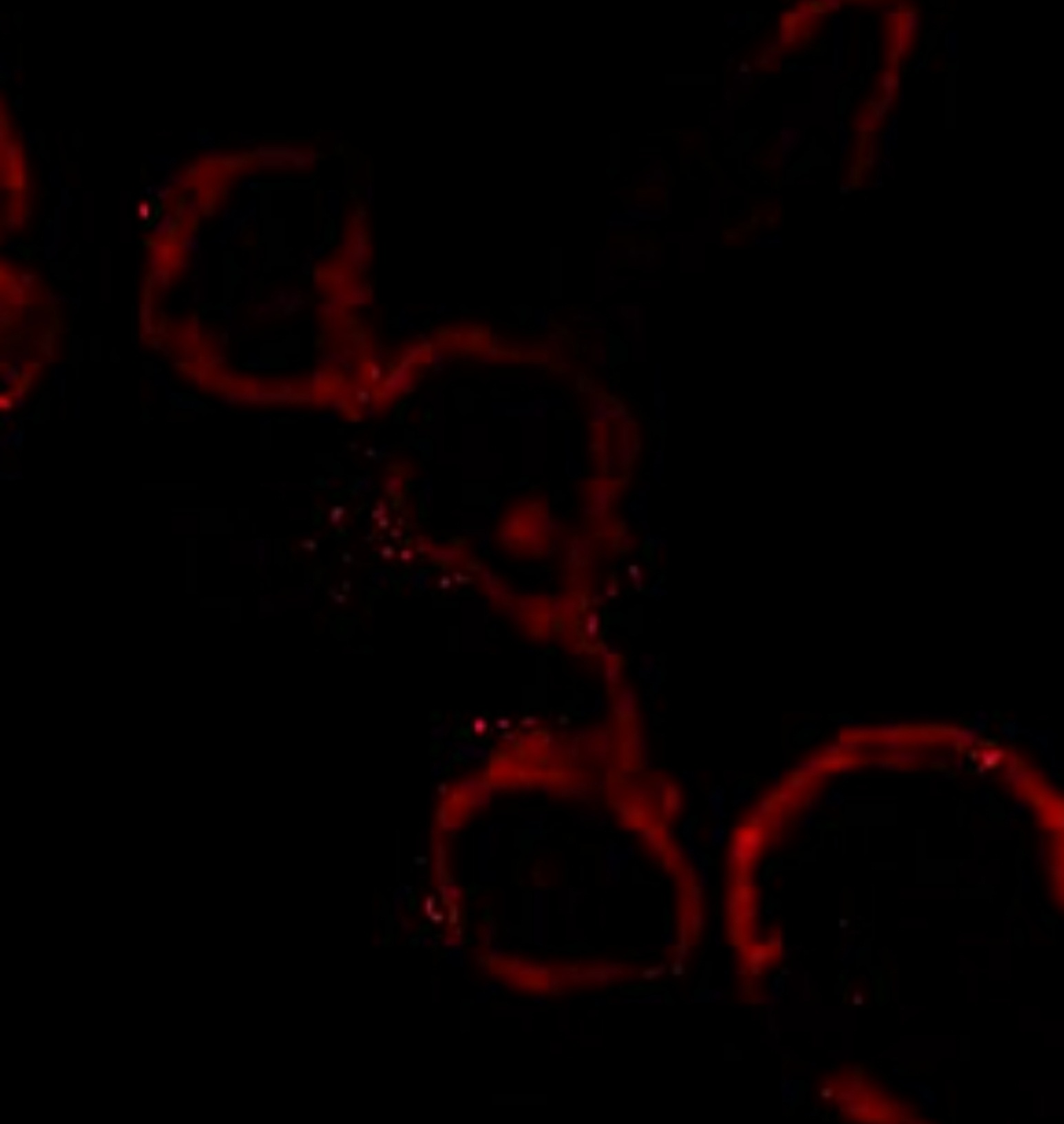

Supplement: Supplementary Figure S1 — Light micrographs of non-infected placental explants stained with Kinyoun at baseline (4 h). [file Data_Sheet_1.zip › Supplementary figures/Immunofluorescence S13-S57, S71-S73/Figure S27.jpg]

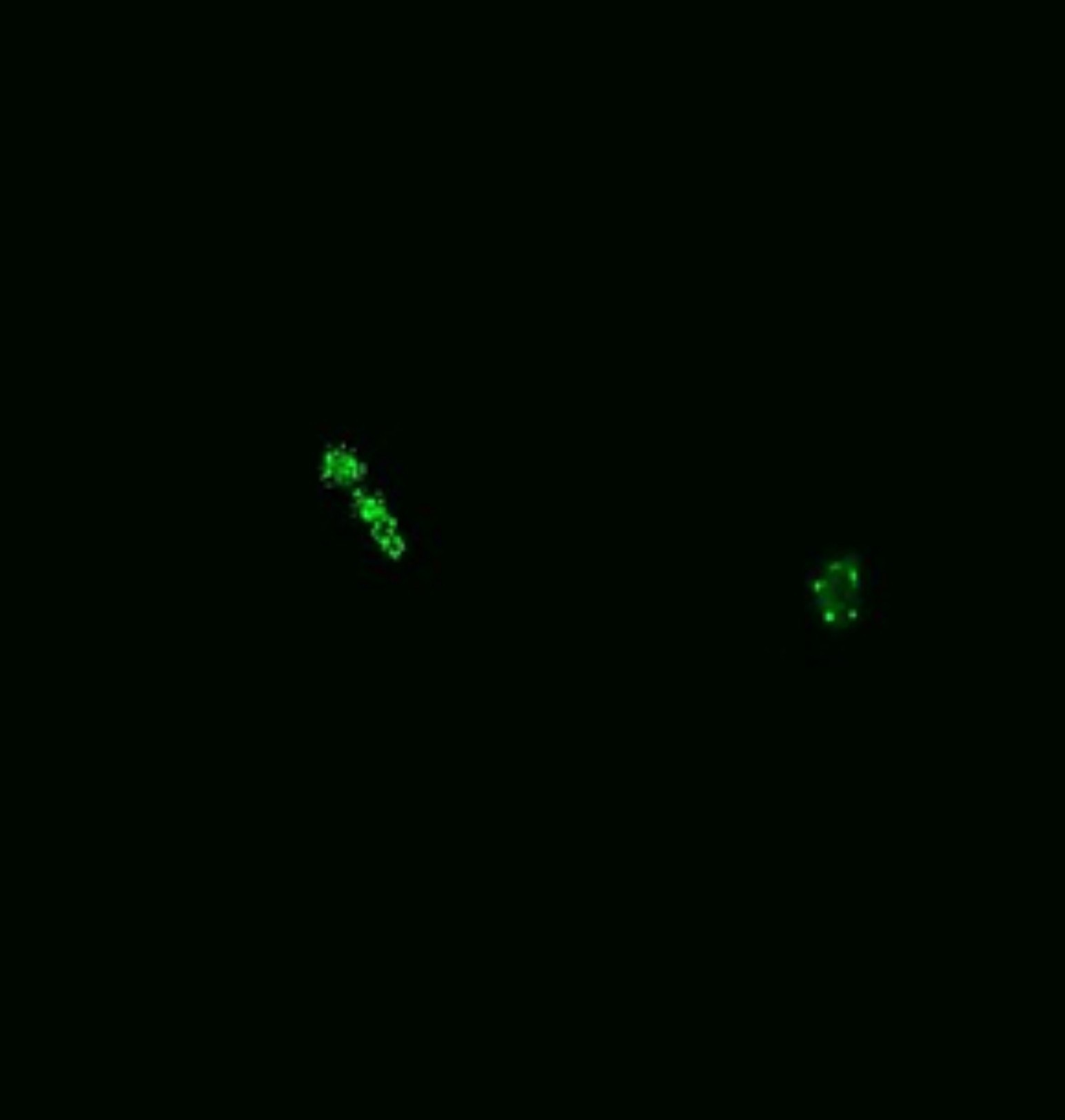

Supplement: Supplementary Figure S1 — Light micrographs of non-infected placental explants stained with Kinyoun at baseline (4 h). [file Data_Sheet_1.zip › Supplementary figures/Immunofluorescence S13-S57, S71-S73/Figure S28.jpg]

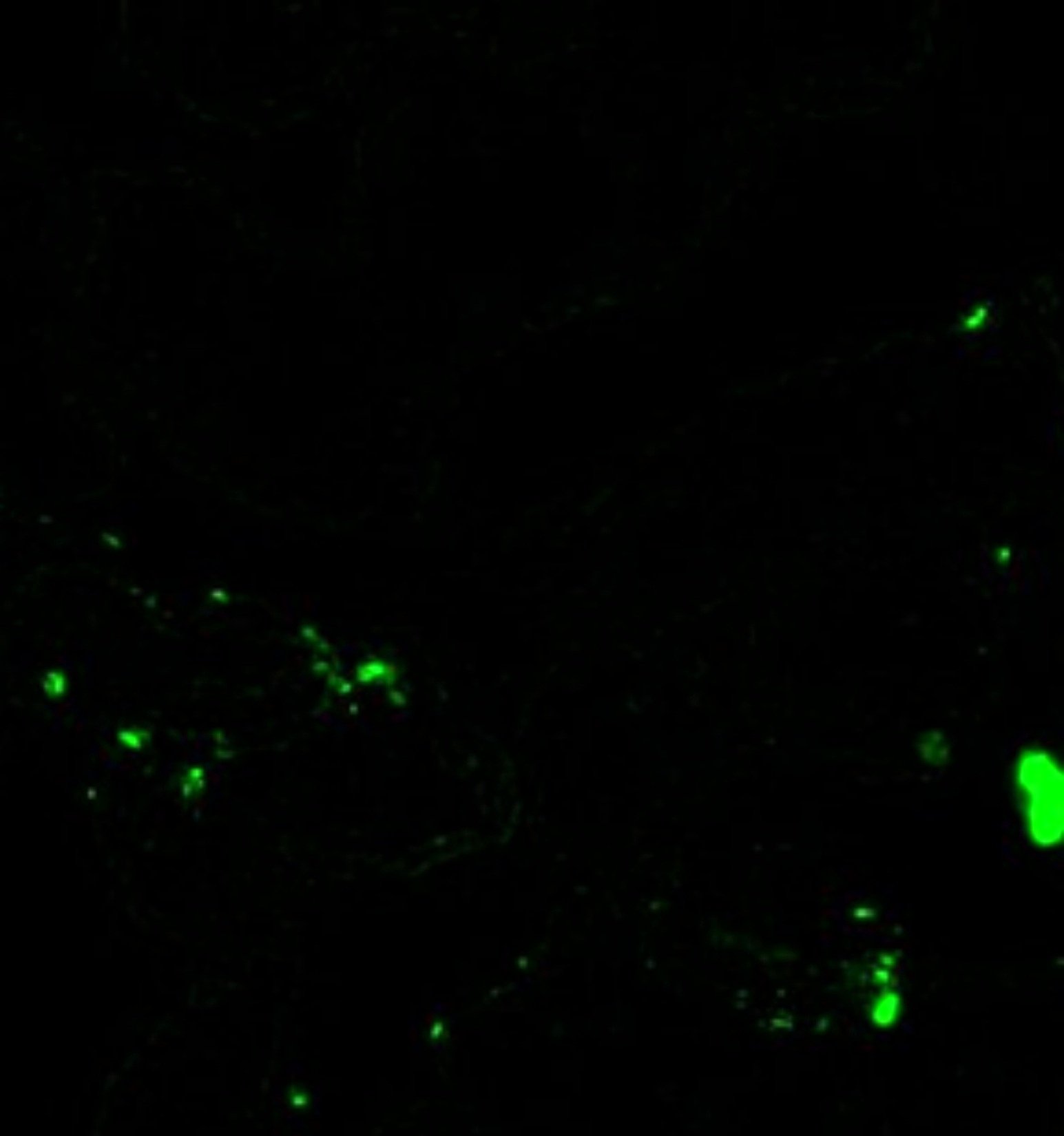

Supplement: Supplementary Figure S1 — Light micrographs of non-infected placental explants stained with Kinyoun at baseline (4 h). [file Data_Sheet_1.zip › Supplementary figures/Immunofluorescence S13-S57, S71-S73/Figure S29.jpg]

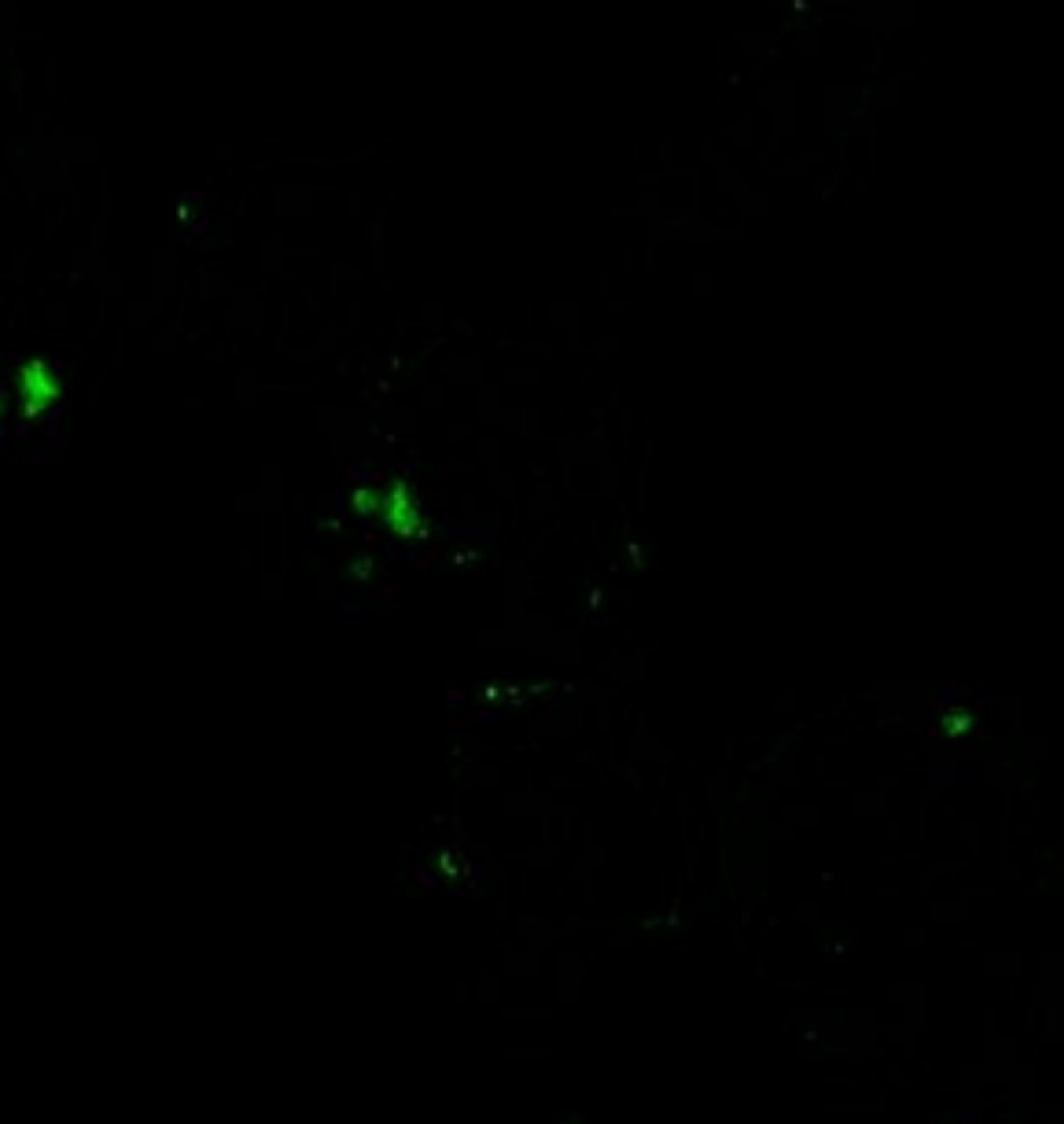

Supplement: Supplementary Figure S1 — Light micrographs of non-infected placental explants stained with Kinyoun at baseline (4 h). [file Data_Sheet_1.zip › Supplementary figures/Immunofluorescence S13-S57, S71-S73/Figure S30.jpg]

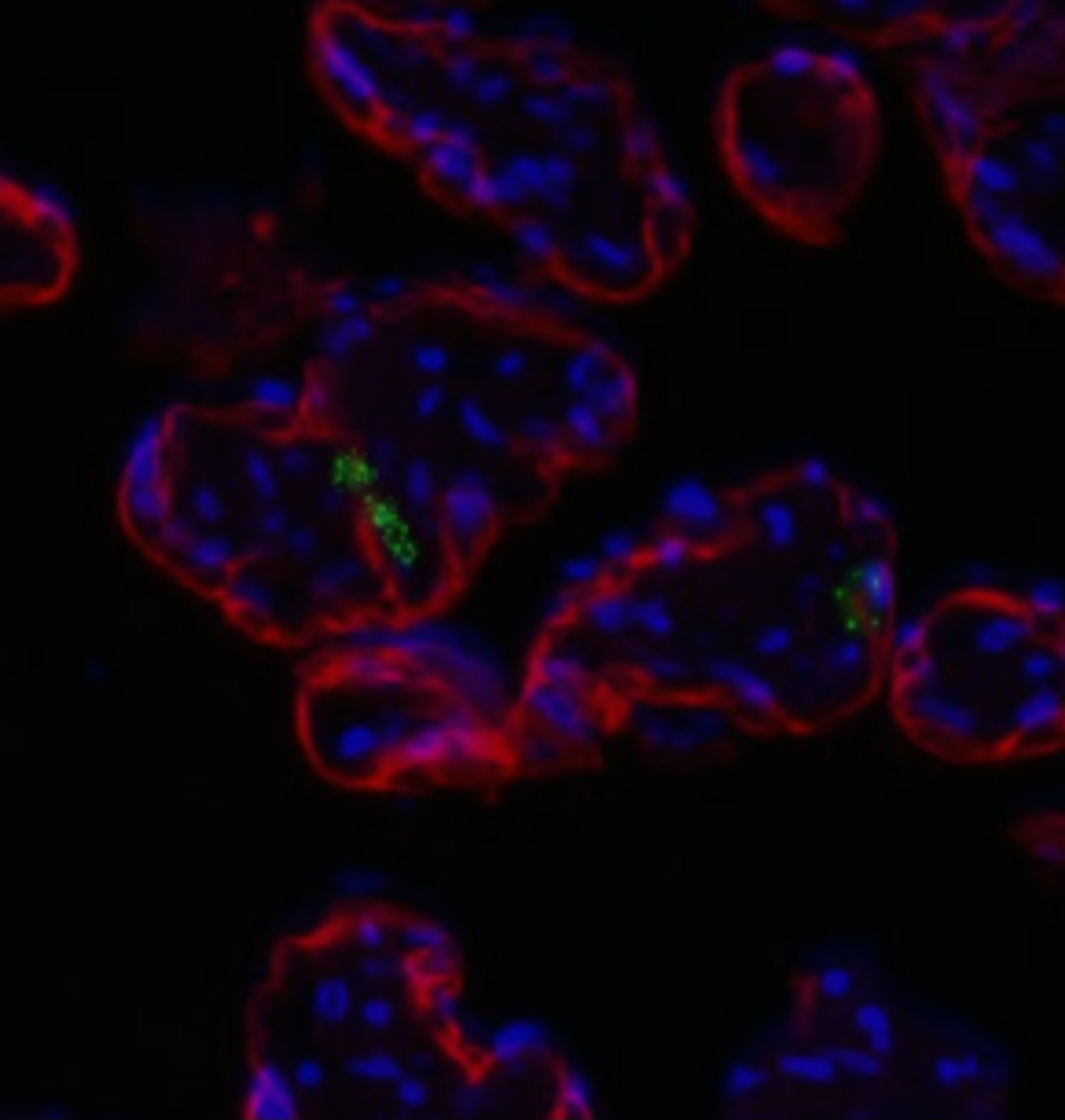

Supplement: Supplementary Figure S1 — Light micrographs of non-infected placental explants stained with Kinyoun at baseline (4 h). [file Data_Sheet_1.zip › Supplementary figures/Immunofluorescence S13-S57, S71-S73/Figure S31.jpg]

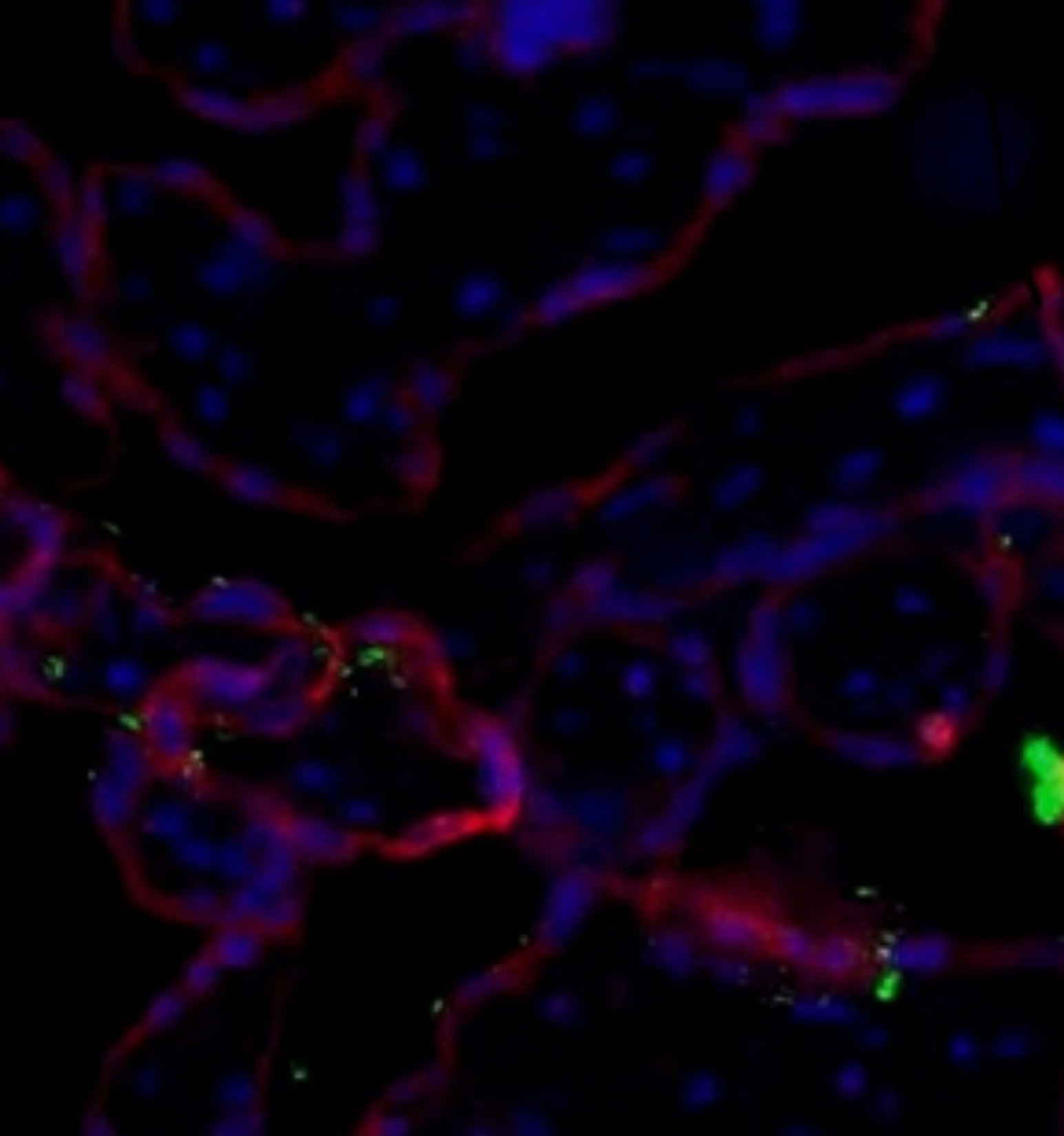

Supplement: Supplementary Figure S1 — Light micrographs of non-infected placental explants stained with Kinyoun at baseline (4 h). [file Data_Sheet_1.zip › Supplementary figures/Immunofluorescence S13-S57, S71-S73/Figure S32.jpg]

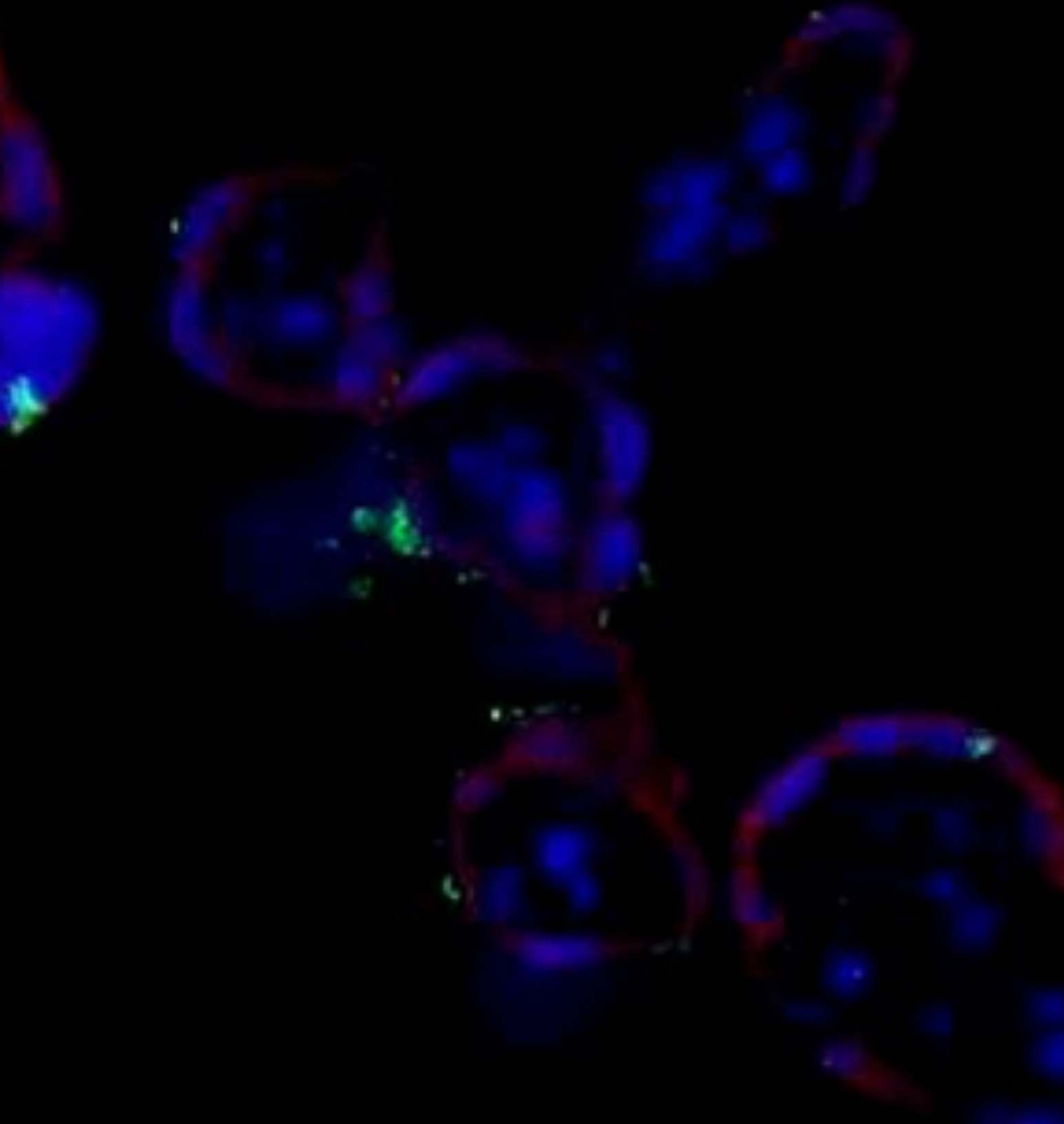

Supplement: Supplementary Figure S1 — Light micrographs of non-infected placental explants stained with Kinyoun at baseline (4 h). [file Data_Sheet_1.zip › Supplementary figures/Immunofluorescence S13-S57, S71-S73/Figure S33.jpg]

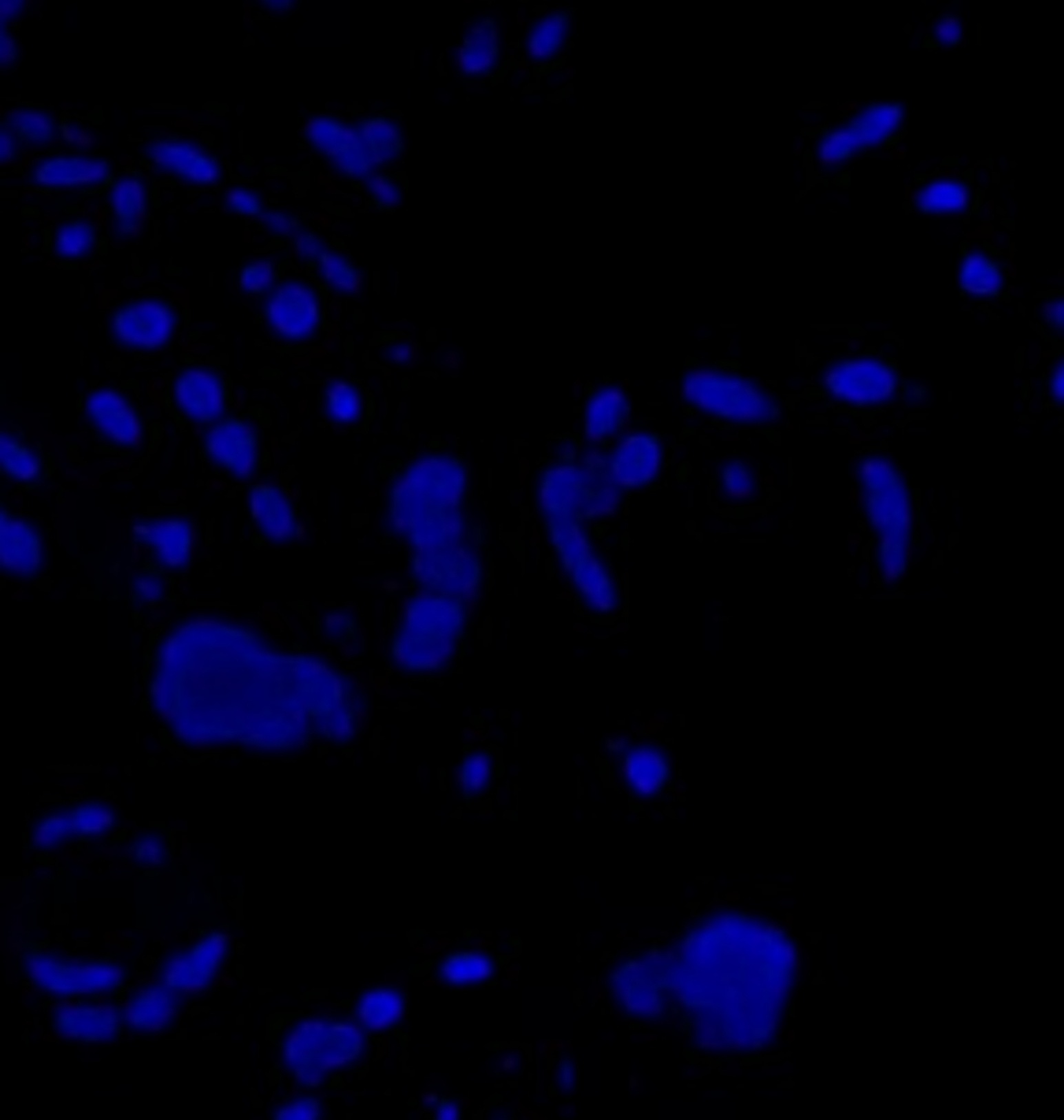

Supplement: Supplementary Figure S1 — Light micrographs of non-infected placental explants stained with Kinyoun at baseline (4 h). [file Data_Sheet_1.zip › Supplementary figures/Immunofluorescence S13-S57, S71-S73/Figure S34.jpg]

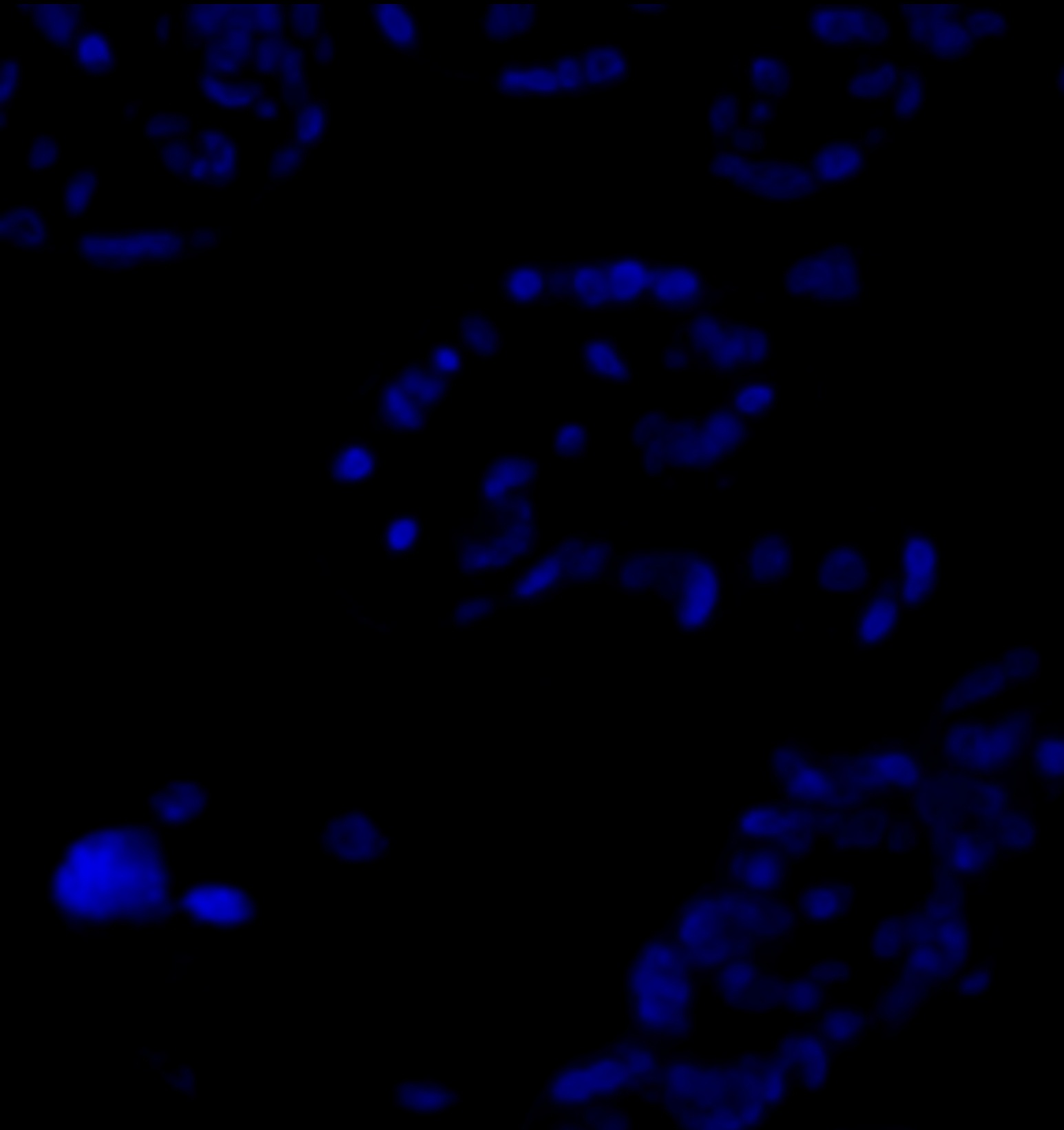

Supplement: Supplementary Figure S1 — Light micrographs of non-infected placental explants stained with Kinyoun at baseline (4 h). [file Data_Sheet_1.zip › Supplementary figures/Immunofluorescence S13-S57, S71-S73/Figure S35.jpg]

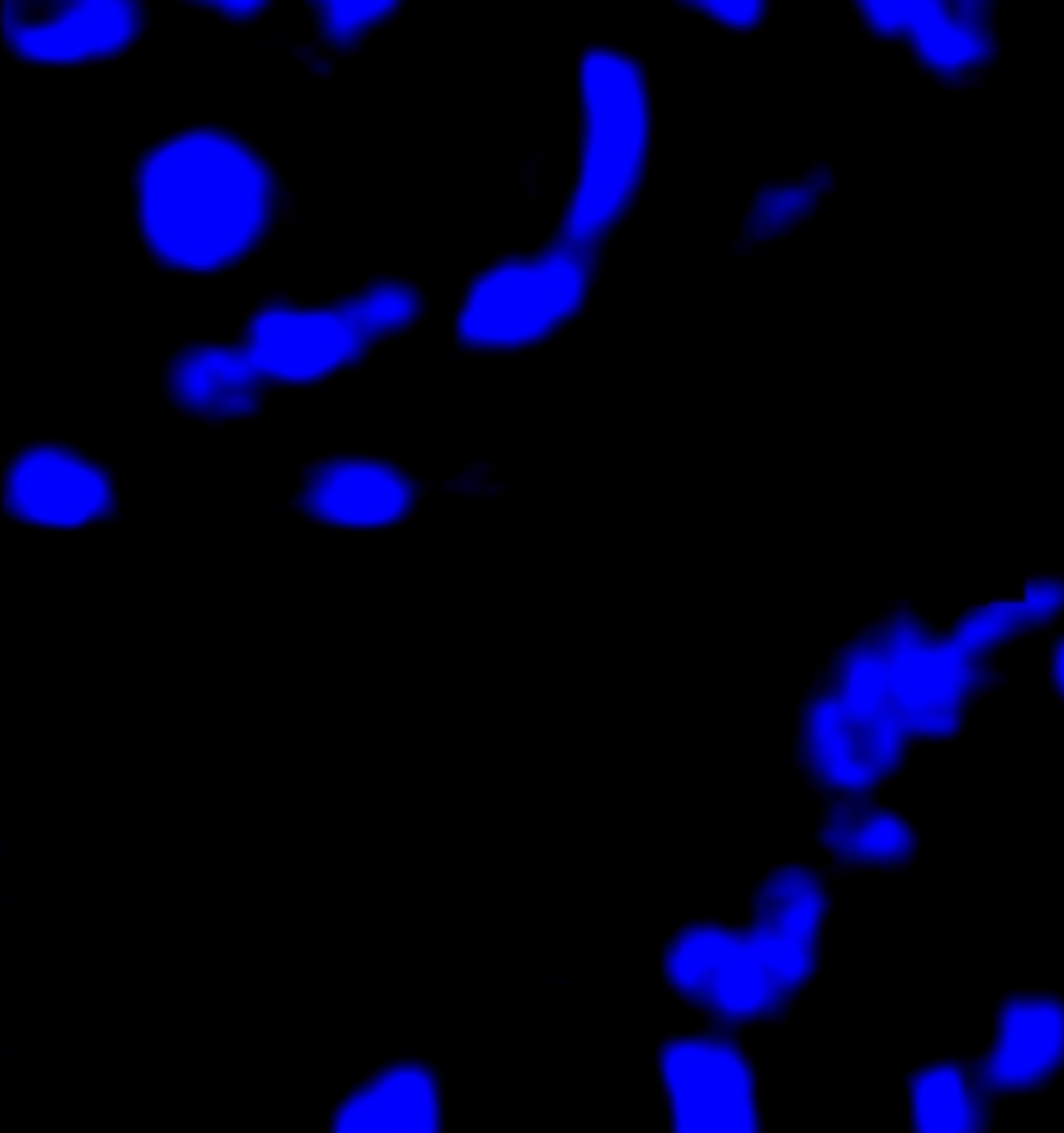

Supplement: Supplementary Figure S1 — Light micrographs of non-infected placental explants stained with Kinyoun at baseline (4 h). [file Data_Sheet_1.zip › Supplementary figures/Immunofluorescence S13-S57, S71-S73/Figure S36.jpg]

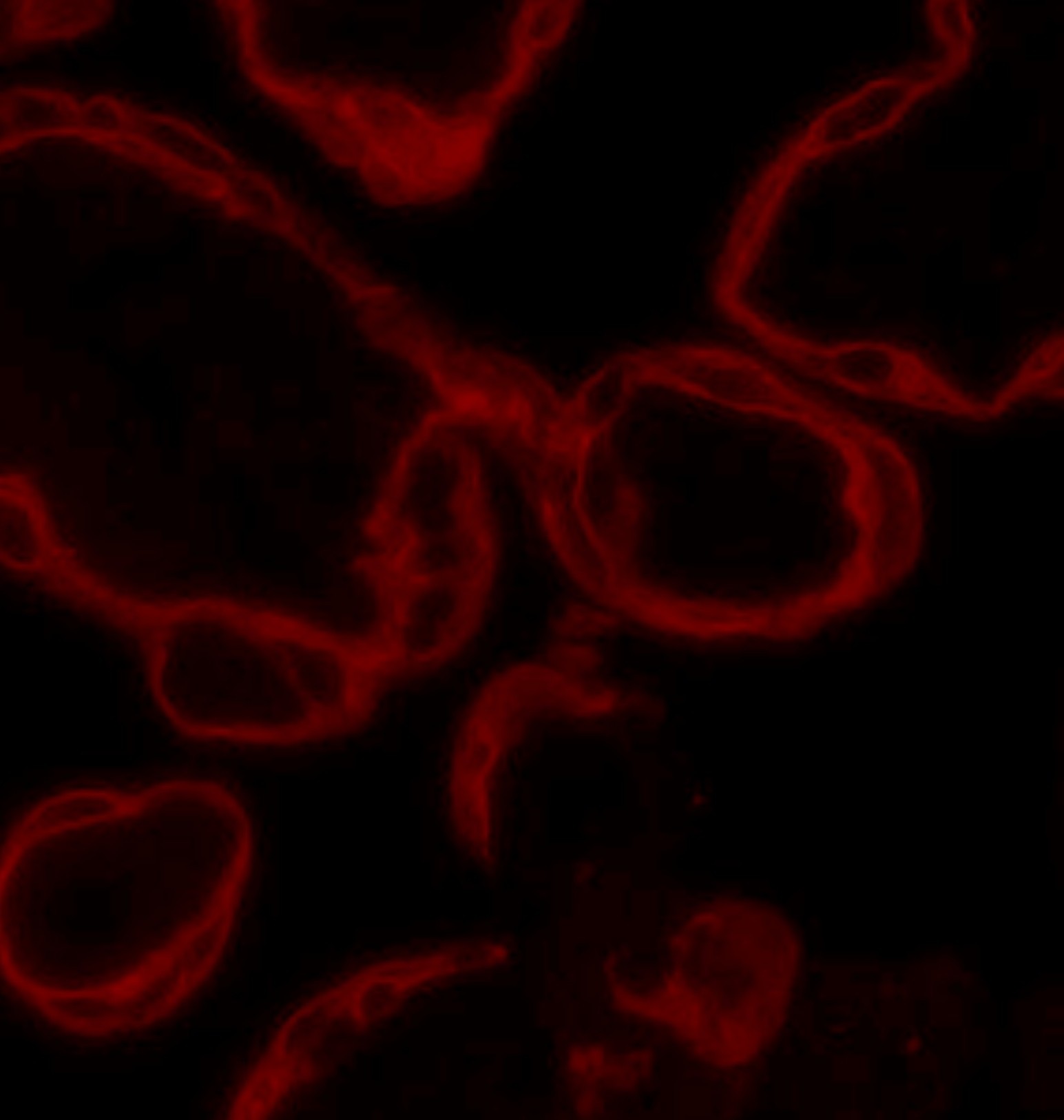

Supplement: Supplementary Figure S1 — Light micrographs of non-infected placental explants stained with Kinyoun at baseline (4 h). [file Data_Sheet_1.zip › Supplementary figures/Immunofluorescence S13-S57, S71-S73/Figure S37.jpg]

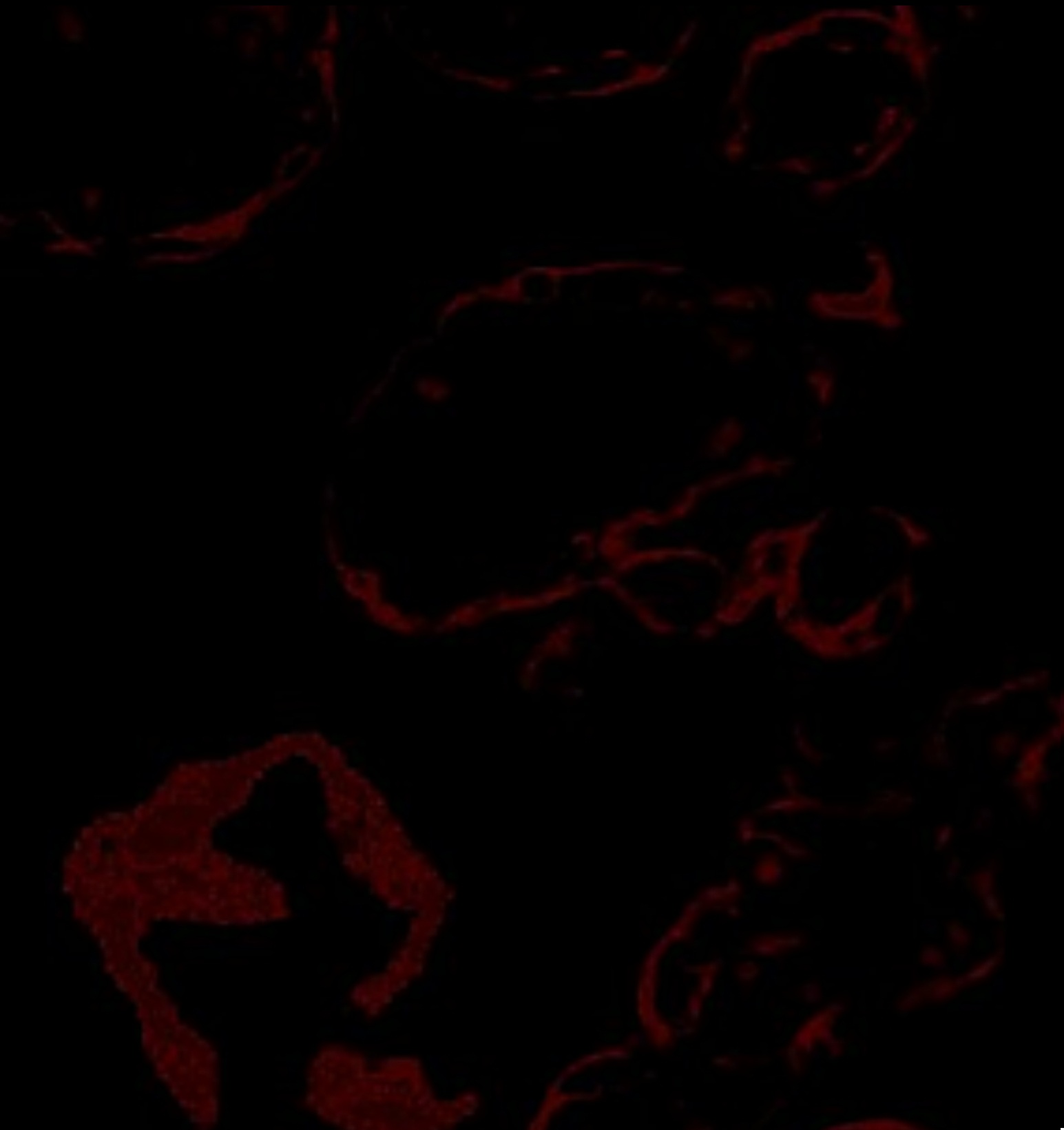

Supplement: Supplementary Figure S1 — Light micrographs of non-infected placental explants stained with Kinyoun at baseline (4 h). [file Data_Sheet_1.zip › Supplementary figures/Immunofluorescence S13-S57, S71-S73/Figure S38.jpg]

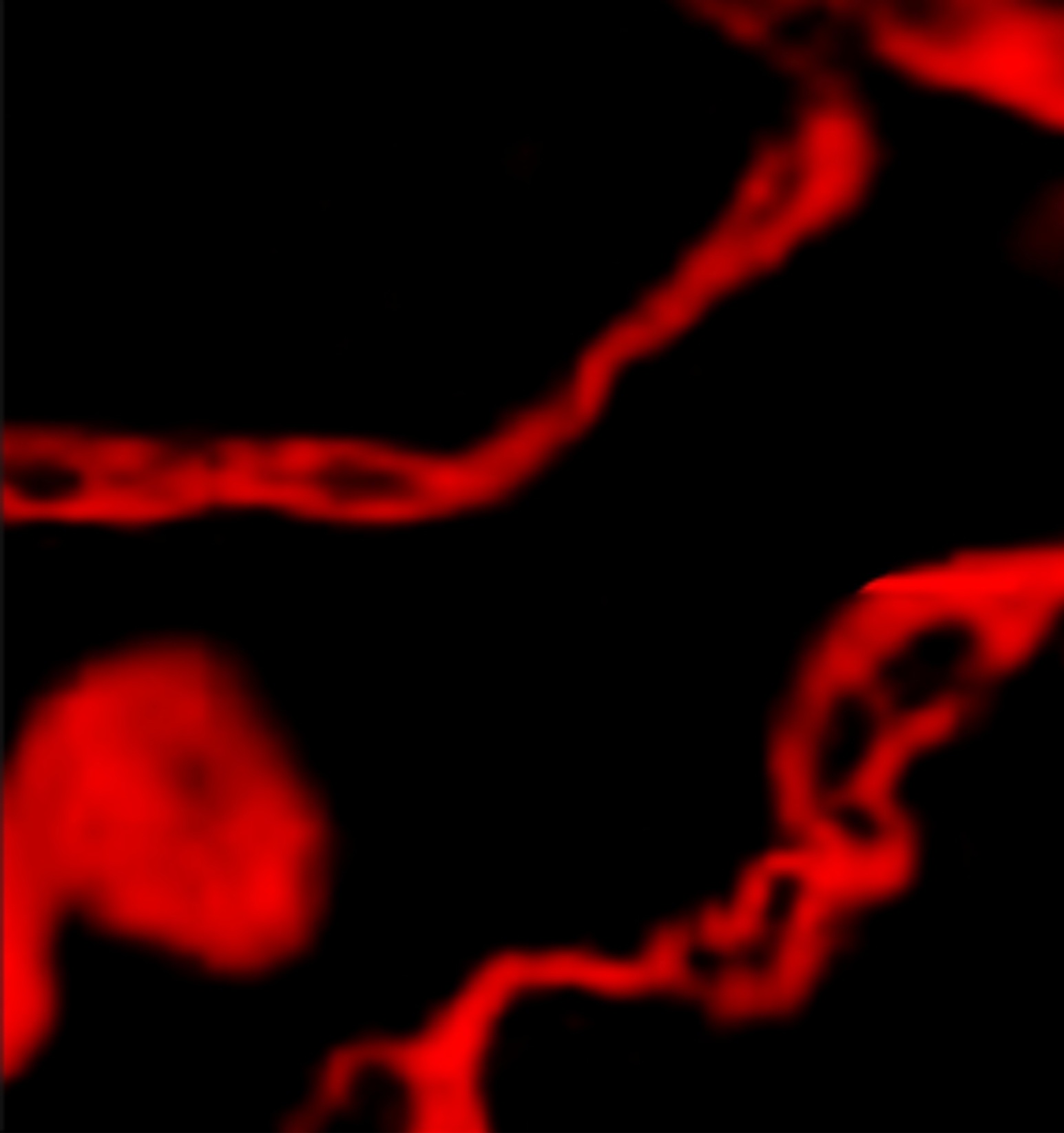

Supplement: Supplementary Figure S1 — Light micrographs of non-infected placental explants stained with Kinyoun at baseline (4 h). [file Data_Sheet_1.zip › Supplementary figures/Immunofluorescence S13-S57, S71-S73/Figure S39.jpg]

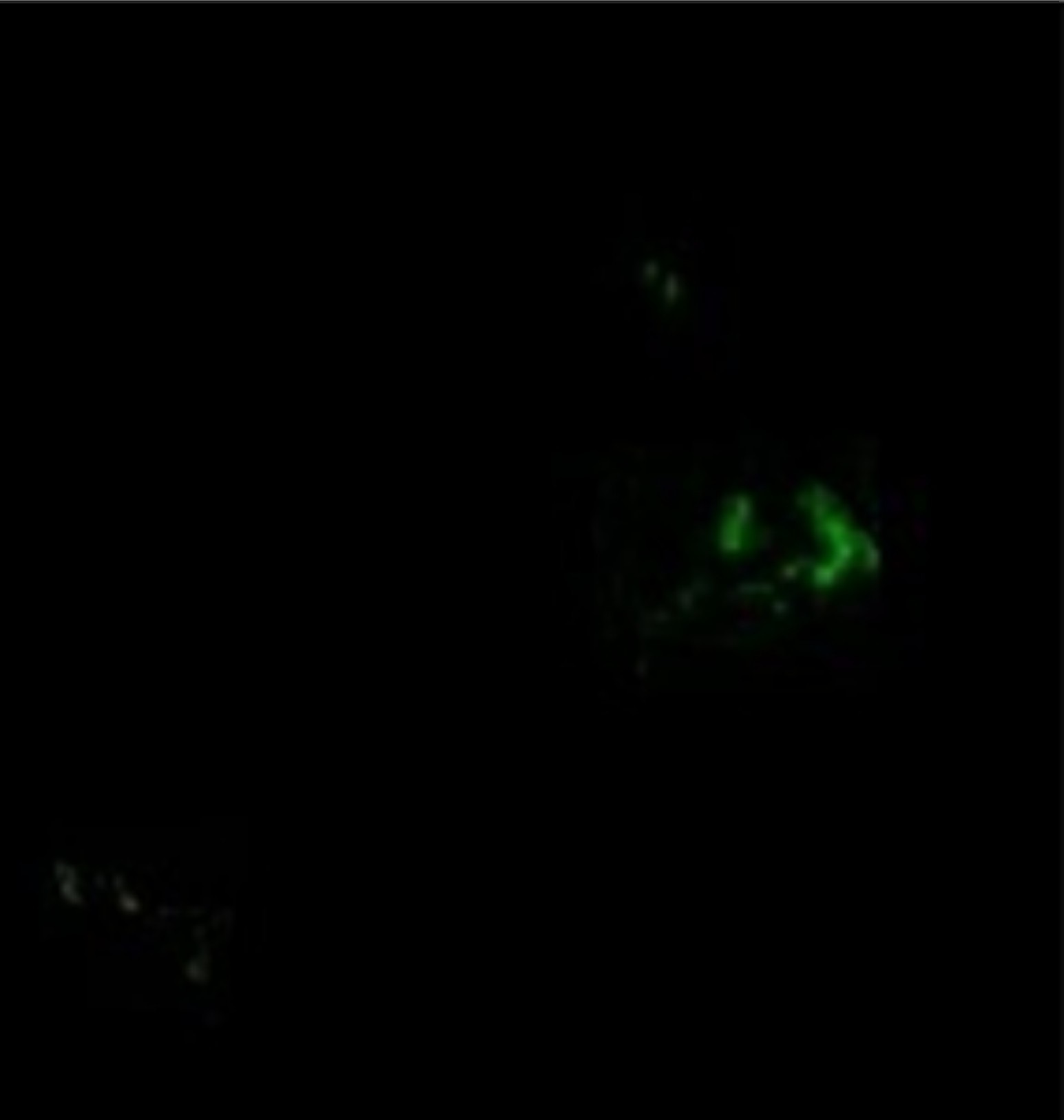

Supplement: Supplementary Figure S1 — Light micrographs of non-infected placental explants stained with Kinyoun at baseline (4 h). [file Data_Sheet_1.zip › Supplementary figures/Immunofluorescence S13-S57, S71-S73/Figure S40.jpg]

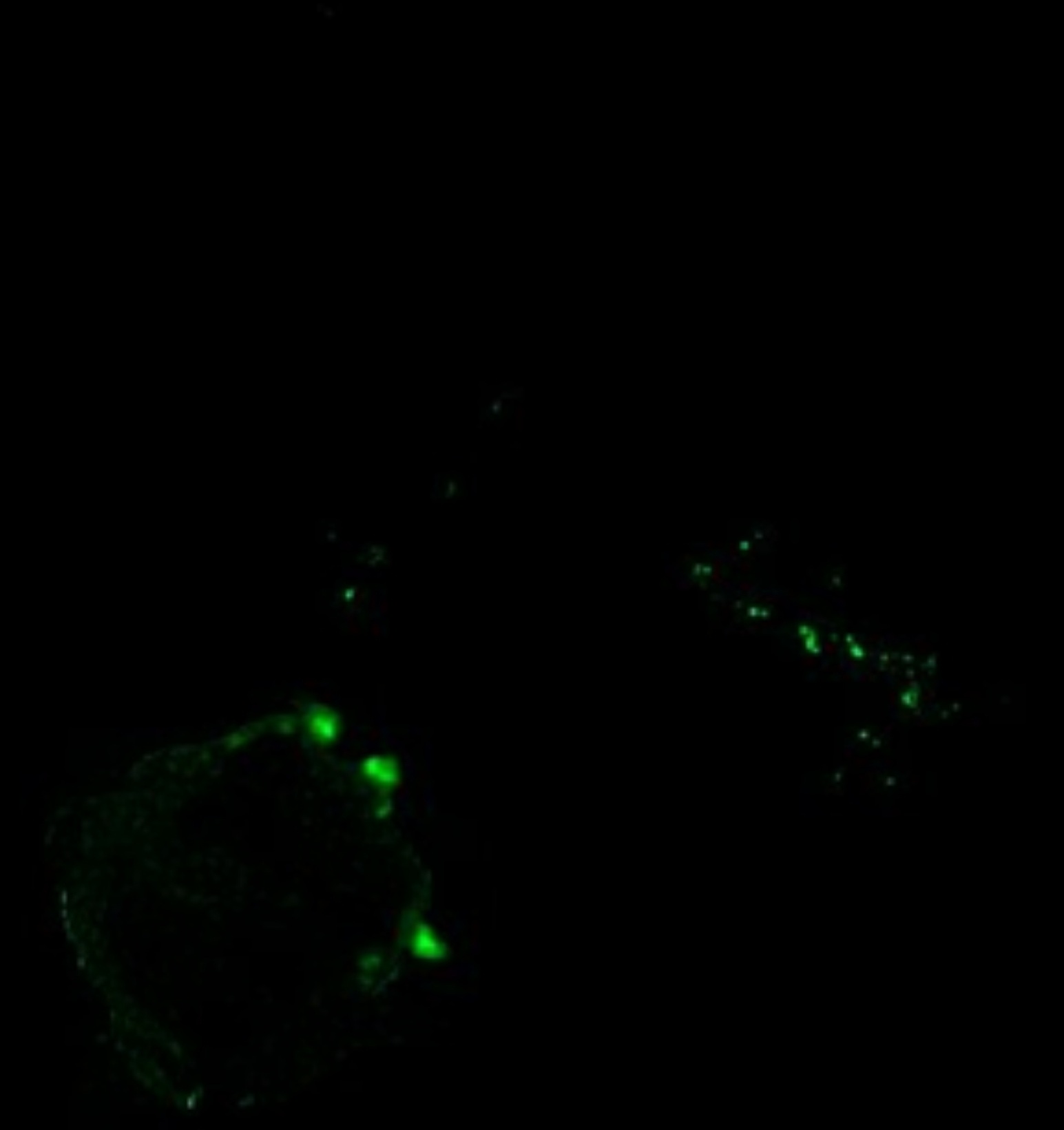

Supplement: Supplementary Figure S1 — Light micrographs of non-infected placental explants stained with Kinyoun at baseline (4 h). [file Data_Sheet_1.zip › Supplementary figures/Immunofluorescence S13-S57, S71-S73/Figure S41.jpg]

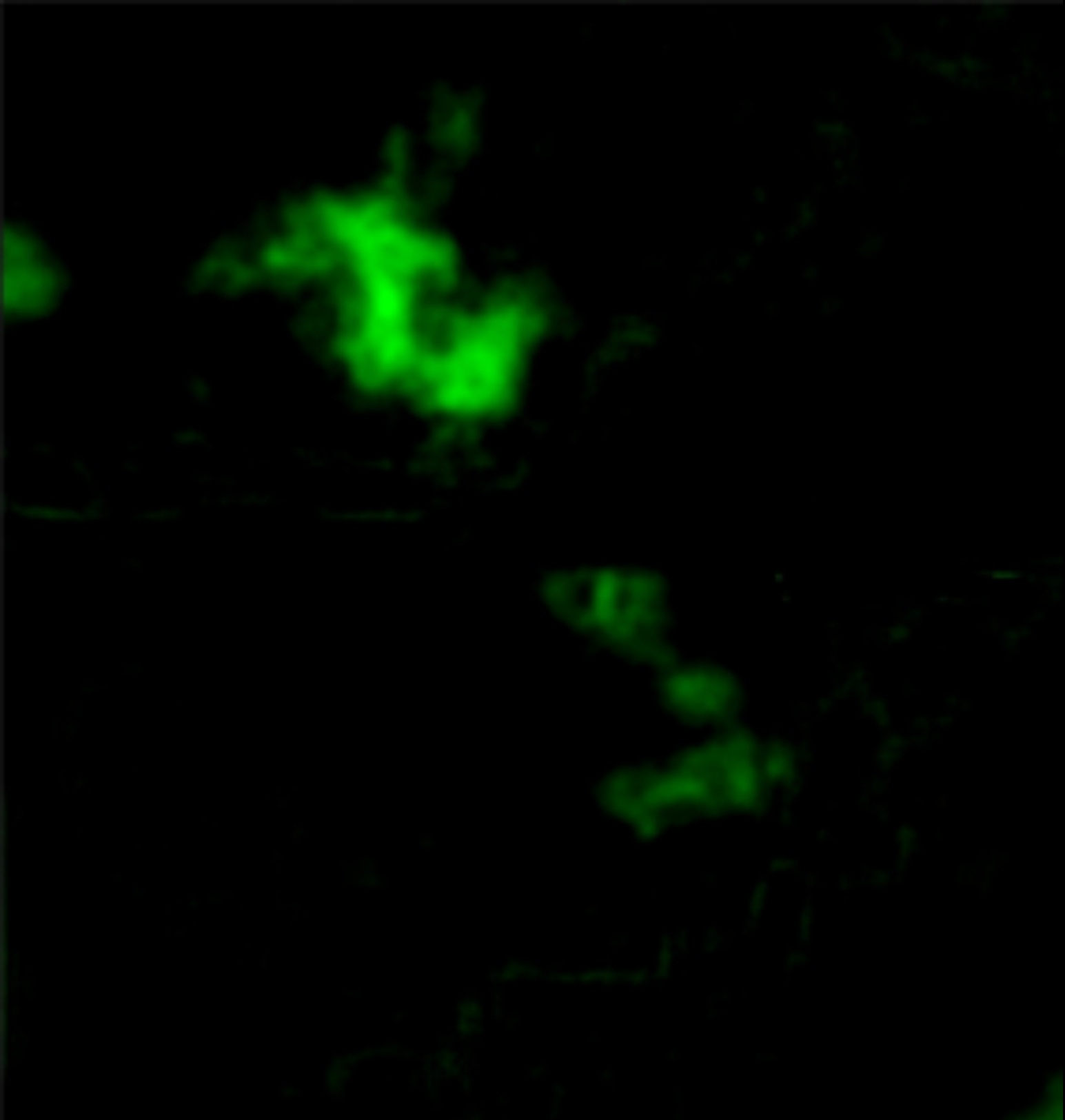

Supplement: Supplementary Figure S1 — Light micrographs of non-infected placental explants stained with Kinyoun at baseline (4 h). [file Data_Sheet_1.zip › Supplementary figures/Immunofluorescence S13-S57, S71-S73/Figure S42.jpg]

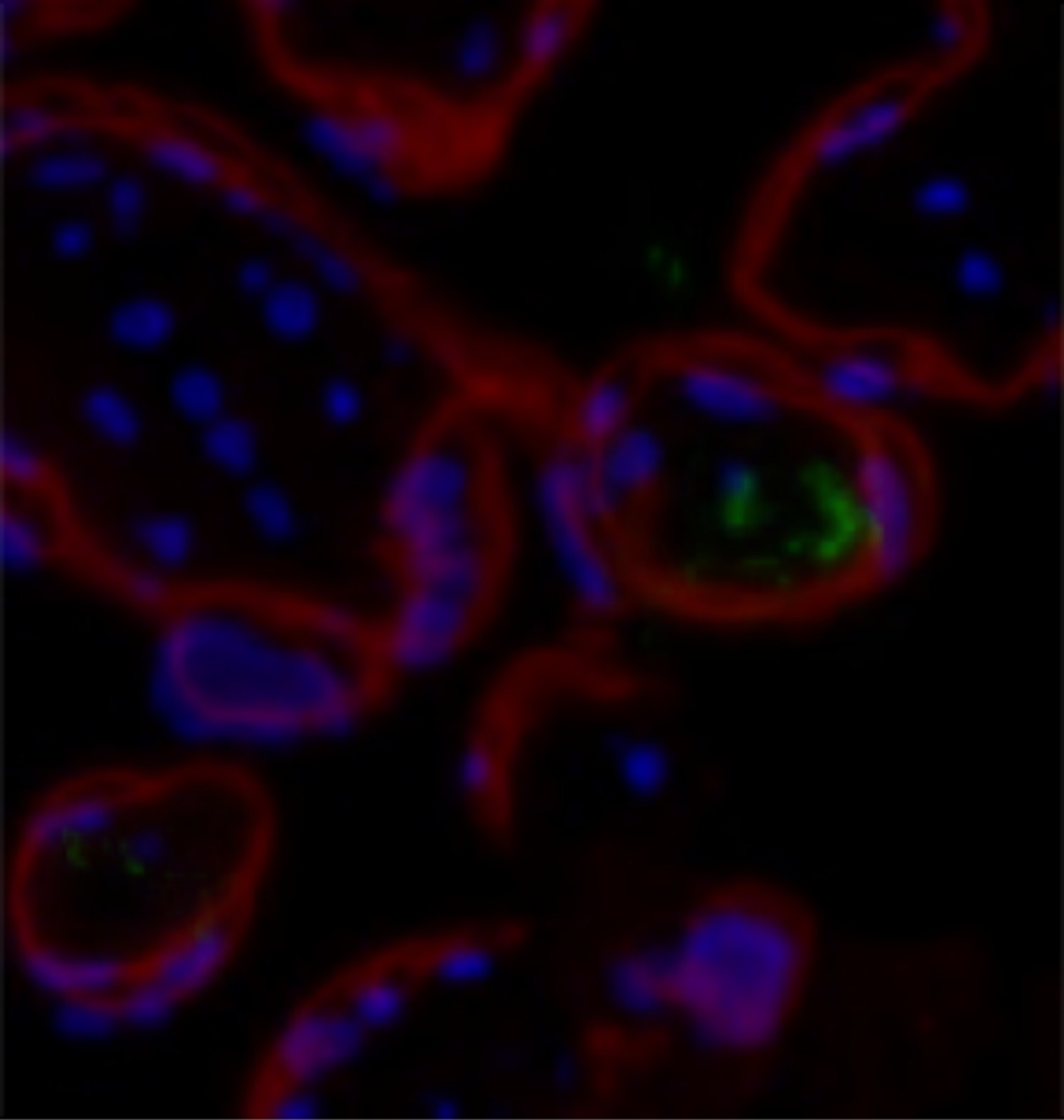

Supplement: Supplementary Figure S1 — Light micrographs of non-infected placental explants stained with Kinyoun at baseline (4 h). [file Data_Sheet_1.zip › Supplementary figures/Immunofluorescence S13-S57, S71-S73/Figure S43.jpg]

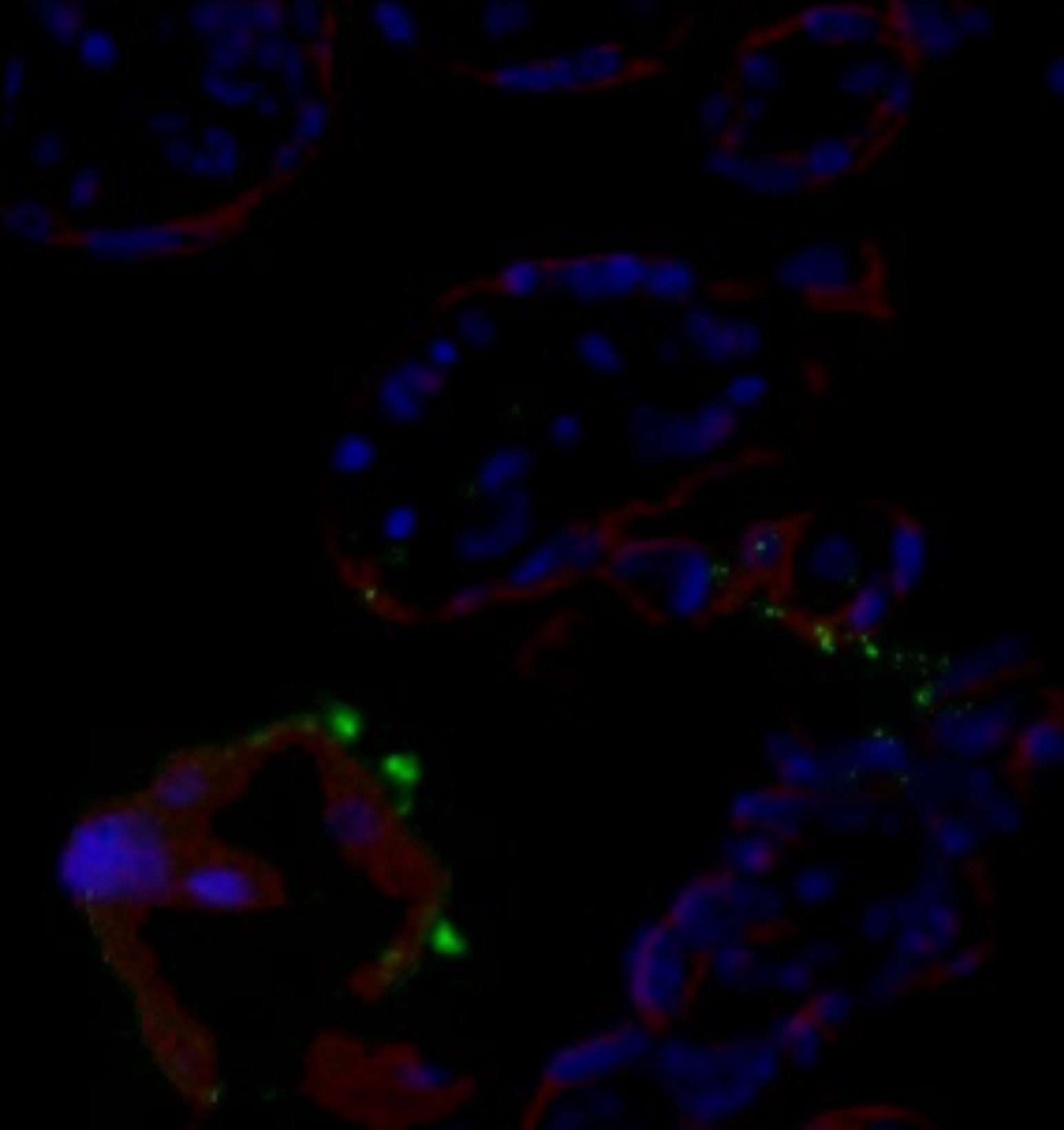

Supplement: Supplementary Figure S1 — Light micrographs of non-infected placental explants stained with Kinyoun at baseline (4 h). [file Data_Sheet_1.zip › Supplementary figures/Immunofluorescence S13-S57, S71-S73/Figure S44.jpg]

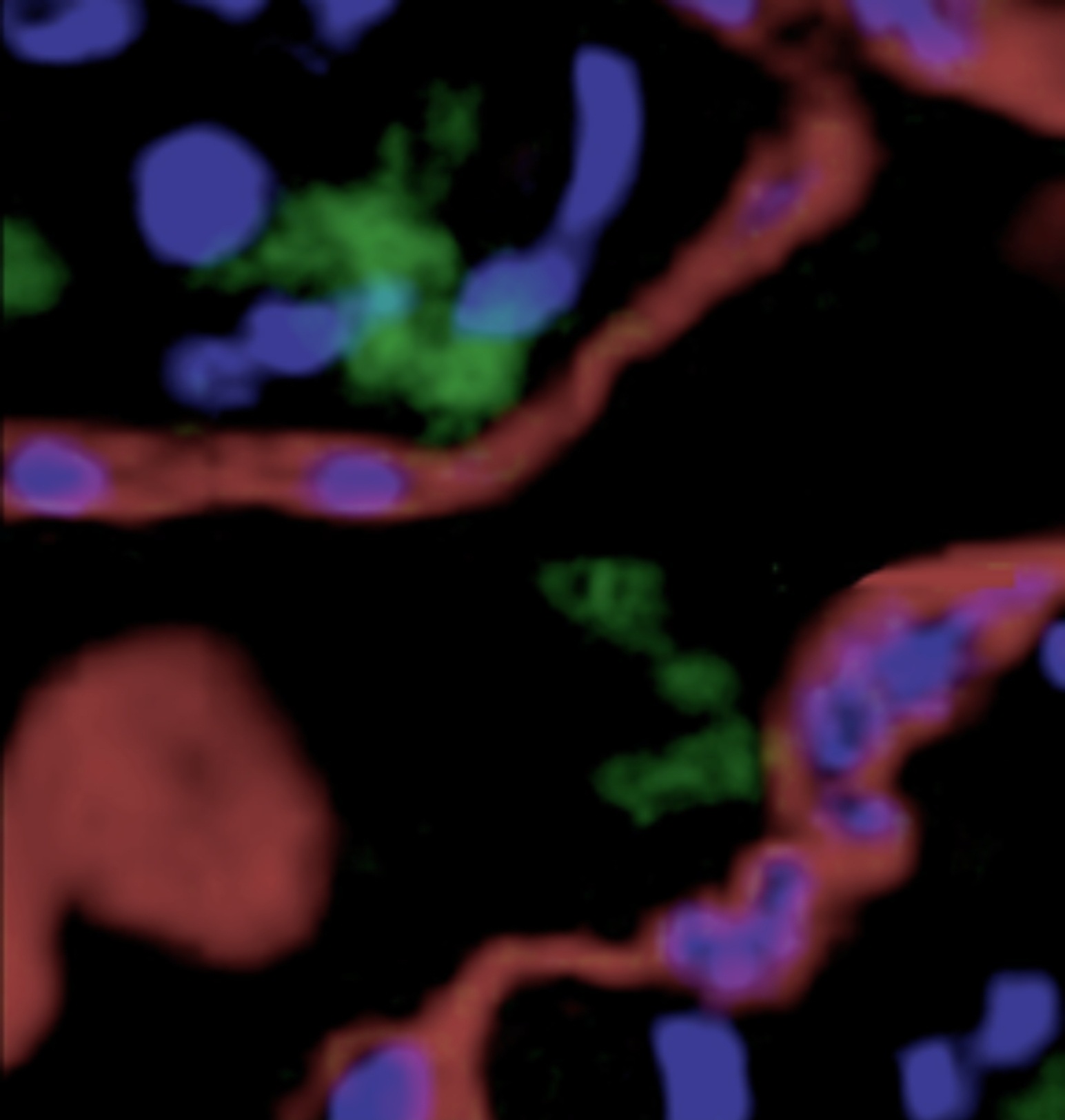

Supplement: Supplementary Figure S1 — Light micrographs of non-infected placental explants stained with Kinyoun at baseline (4 h). [file Data_Sheet_1.zip › Supplementary figures/Immunofluorescence S13-S57, S71-S73/Figure S45.jpg]

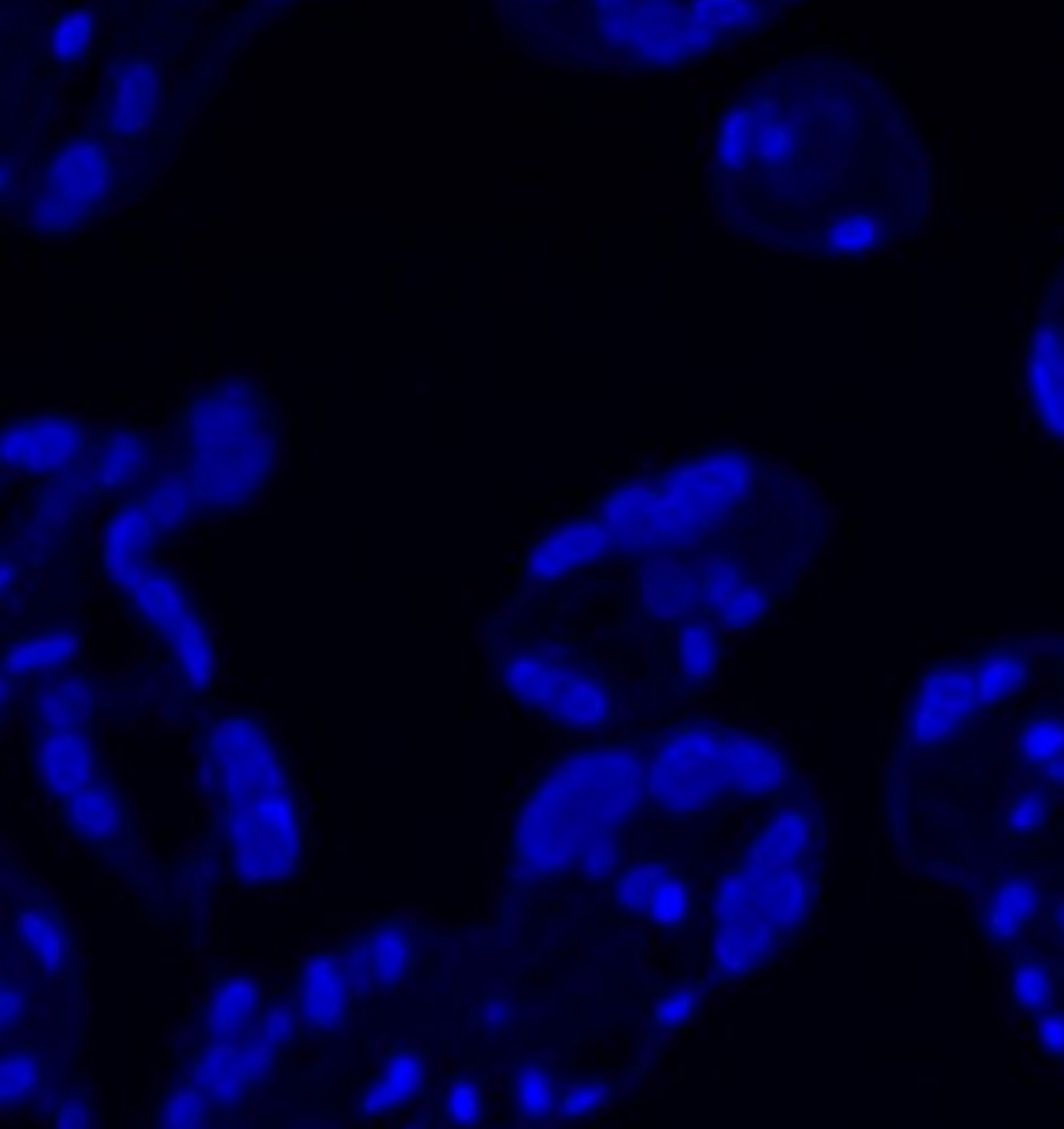

Supplement: Supplementary Figure S1 — Light micrographs of non-infected placental explants stained with Kinyoun at baseline (4 h). [file Data_Sheet_1.zip › Supplementary figures/Immunofluorescence S13-S57, S71-S73/Figure S46.jpg]

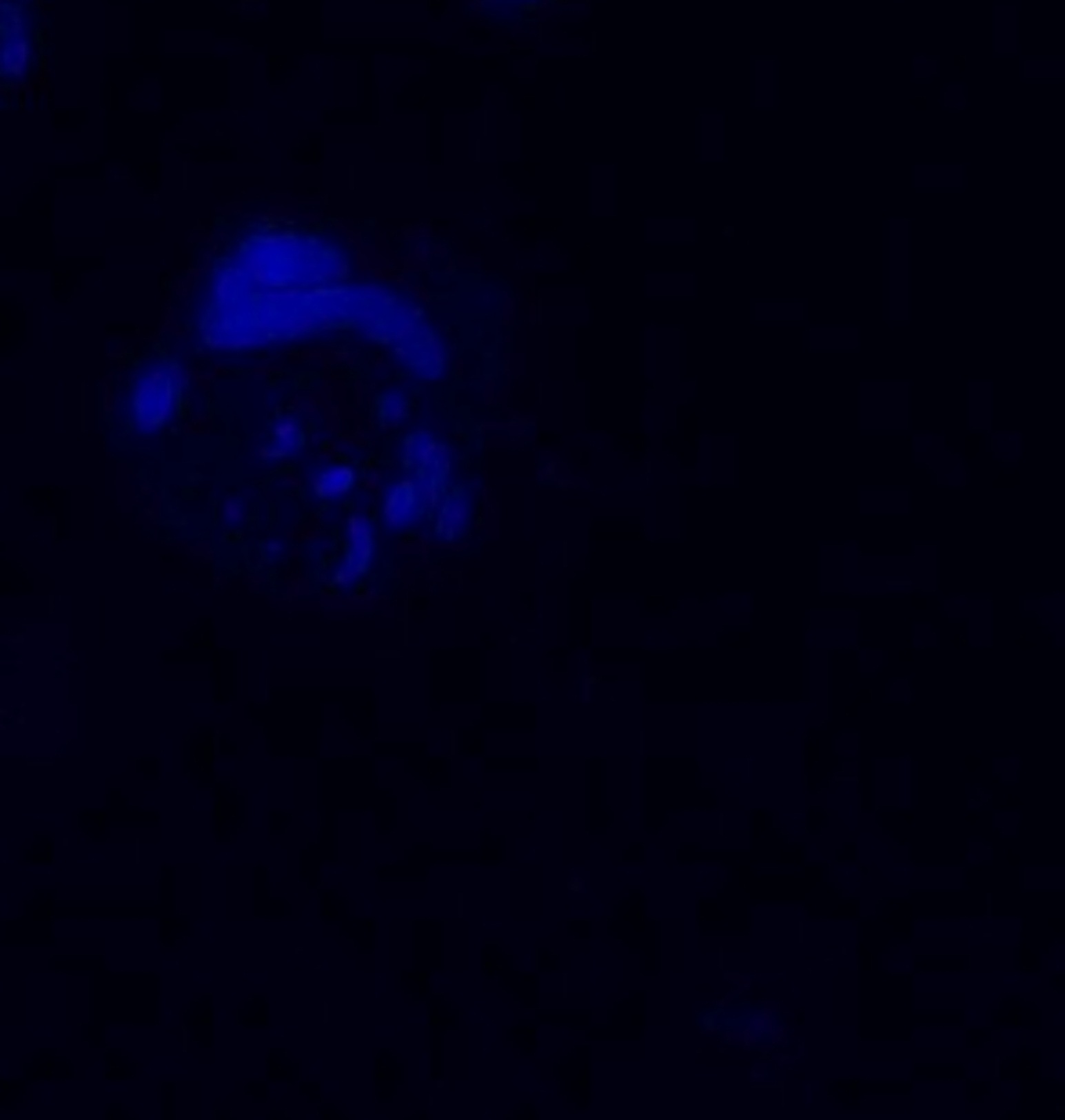

Supplement: Supplementary Figure S1 — Light micrographs of non-infected placental explants stained with Kinyoun at baseline (4 h). [file Data_Sheet_1.zip › Supplementary figures/Immunofluorescence S13-S57, S71-S73/Figure S47.jpg]

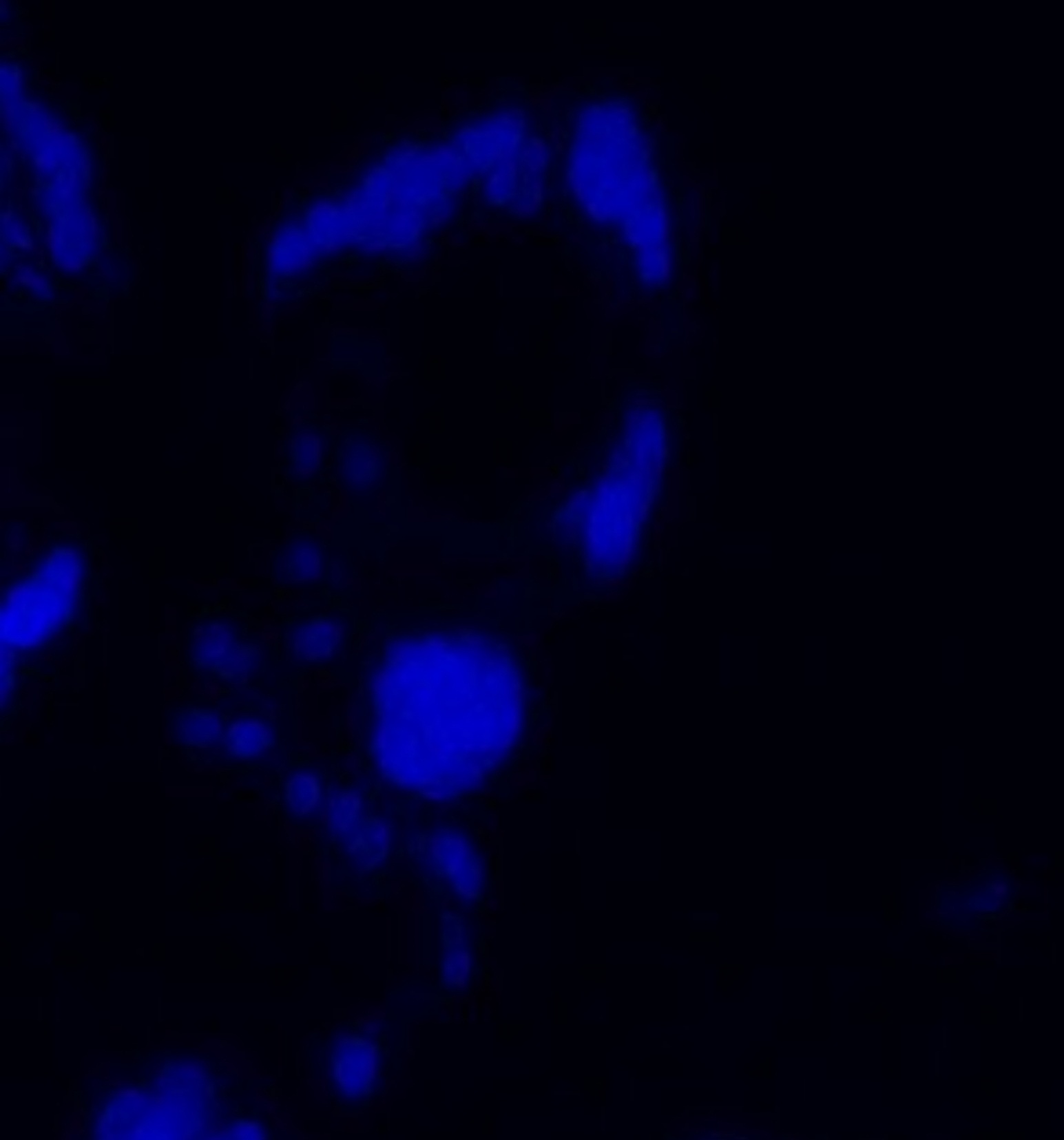

Supplement: Supplementary Figure S1 — Light micrographs of non-infected placental explants stained with Kinyoun at baseline (4 h). [file Data_Sheet_1.zip › Supplementary figures/Immunofluorescence S13-S57, S71-S73/Figure S48.jpg]

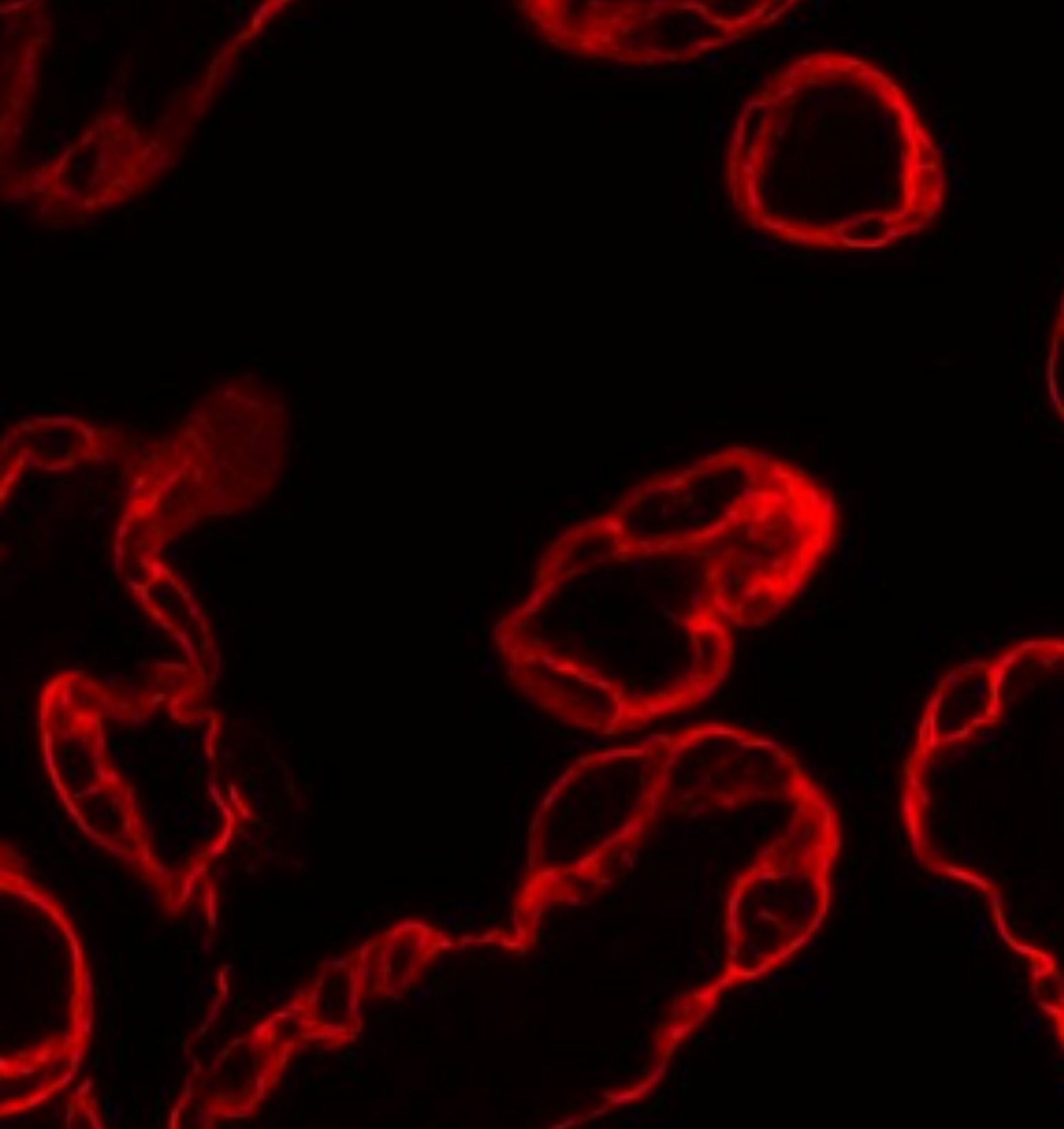

Supplement: Supplementary Figure S1 — Light micrographs of non-infected placental explants stained with Kinyoun at baseline (4 h). [file Data_Sheet_1.zip › Supplementary figures/Immunofluorescence S13-S57, S71-S73/Figure S49.jpg]

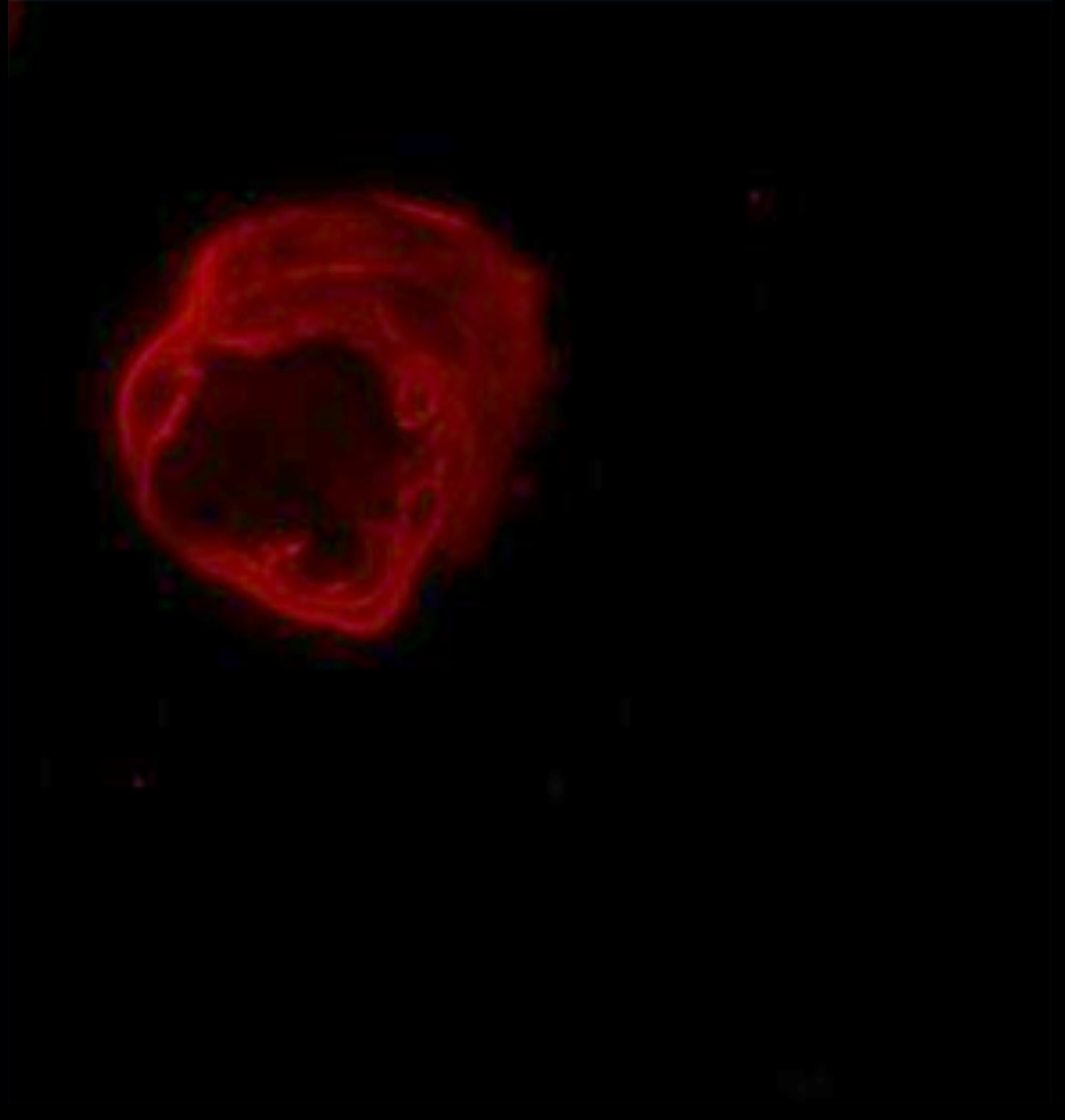

Supplement: Supplementary Figure S1 — Light micrographs of non-infected placental explants stained with Kinyoun at baseline (4 h). [file Data_Sheet_1.zip › Supplementary figures/Immunofluorescence S13-S57, S71-S73/Figure S50.jpg]

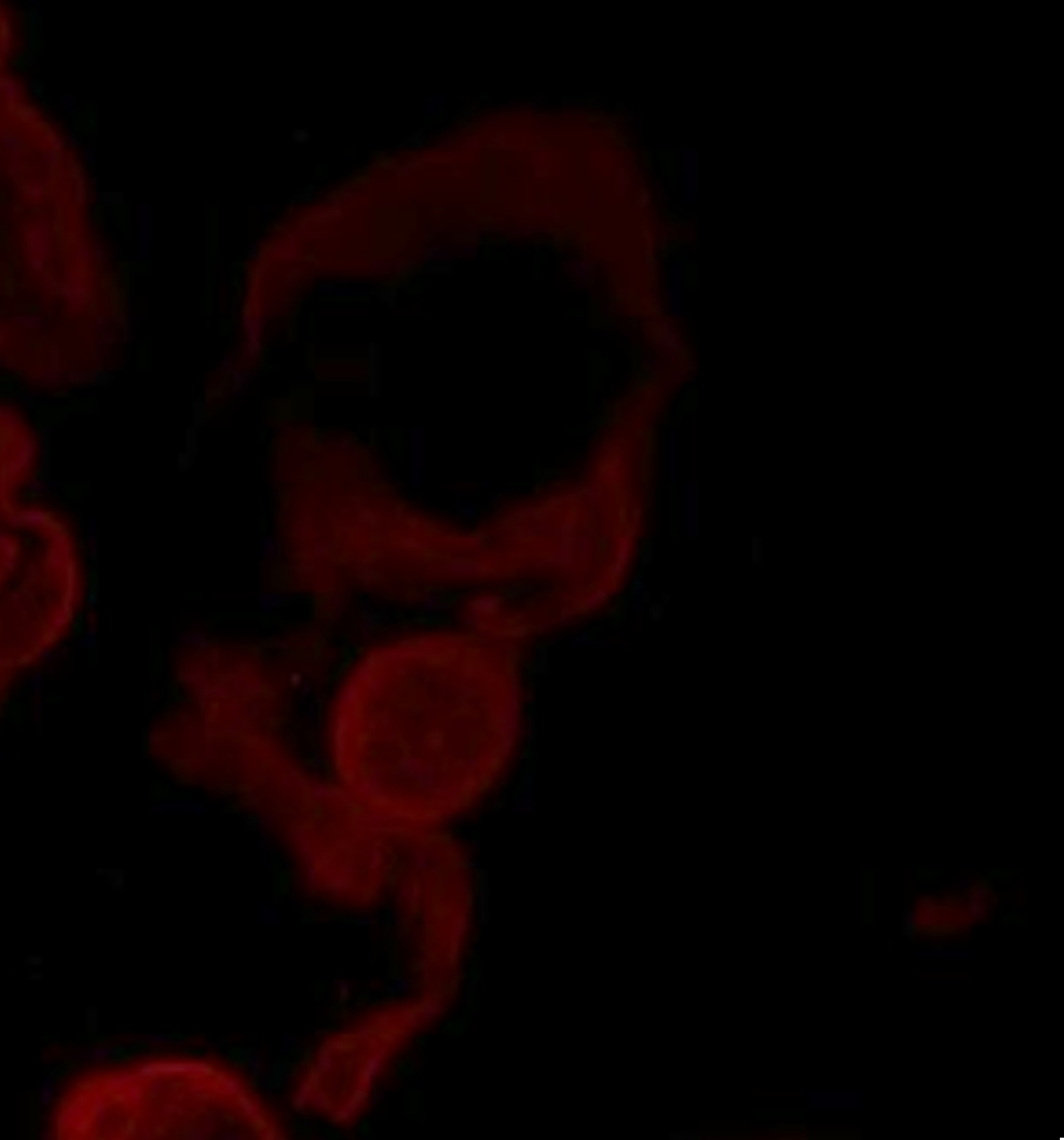

Supplement: Supplementary Figure S1 — Light micrographs of non-infected placental explants stained with Kinyoun at baseline (4 h). [file Data_Sheet_1.zip › Supplementary figures/Immunofluorescence S13-S57, S71-S73/Figure S51.jpg]

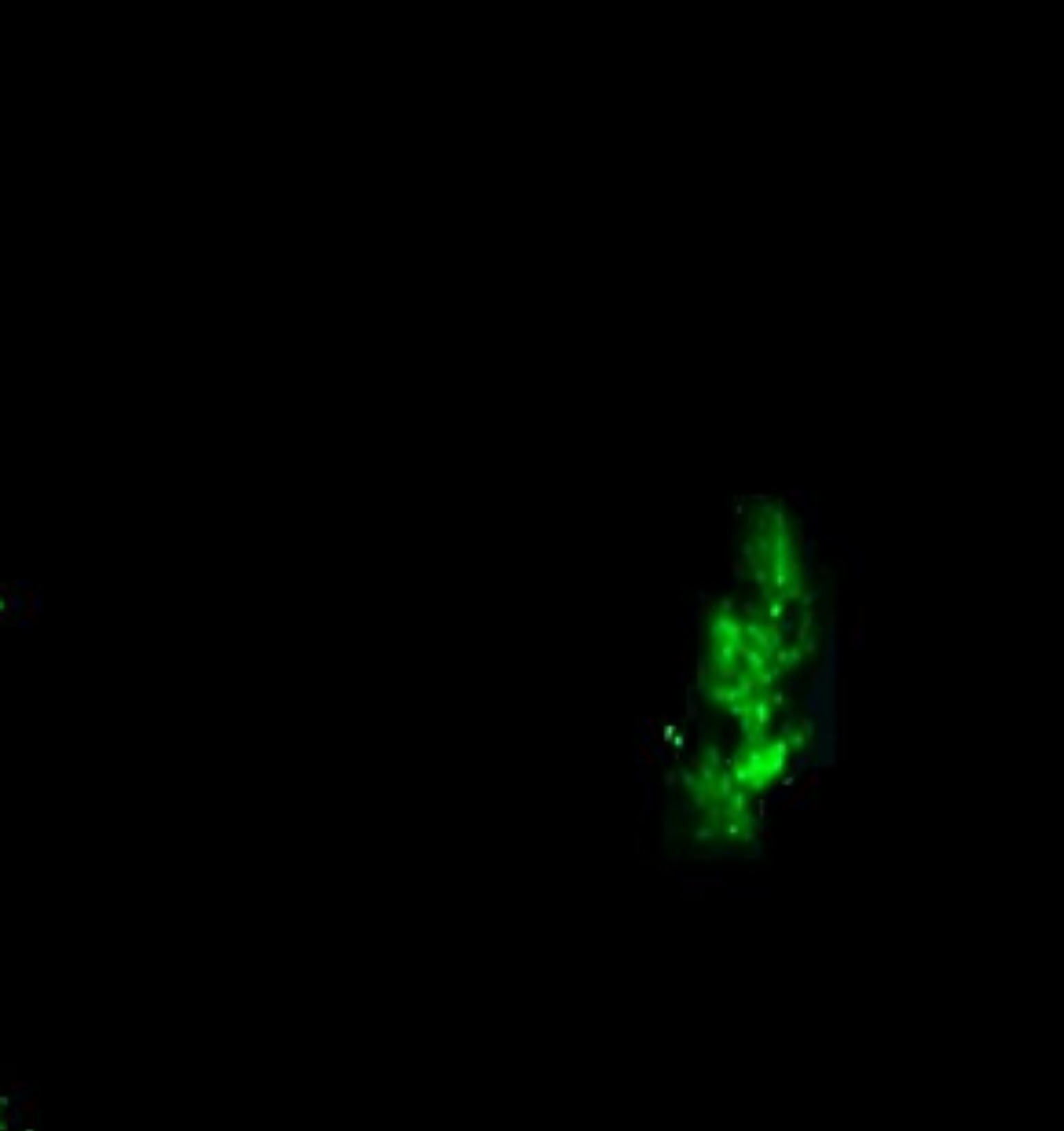

Supplement: Supplementary Figure S1 — Light micrographs of non-infected placental explants stained with Kinyoun at baseline (4 h). [file Data_Sheet_1.zip › Supplementary figures/Immunofluorescence S13-S57, S71-S73/Figure S52.jpg]

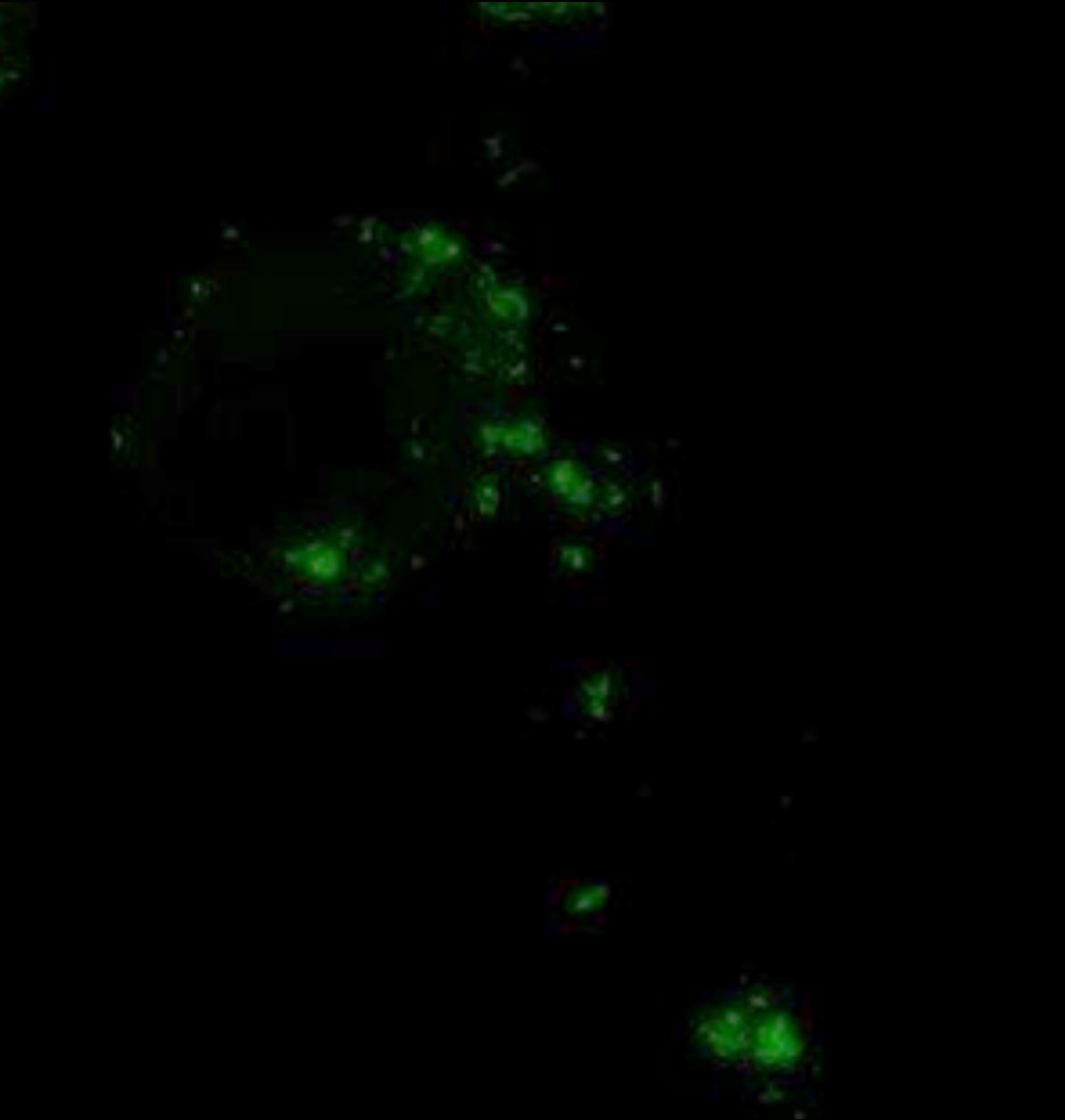

Supplement: Supplementary Figure S1 — Light micrographs of non-infected placental explants stained with Kinyoun at baseline (4 h). [file Data_Sheet_1.zip › Supplementary figures/Immunofluorescence S13-S57, S71-S73/Figure S53.jpg]

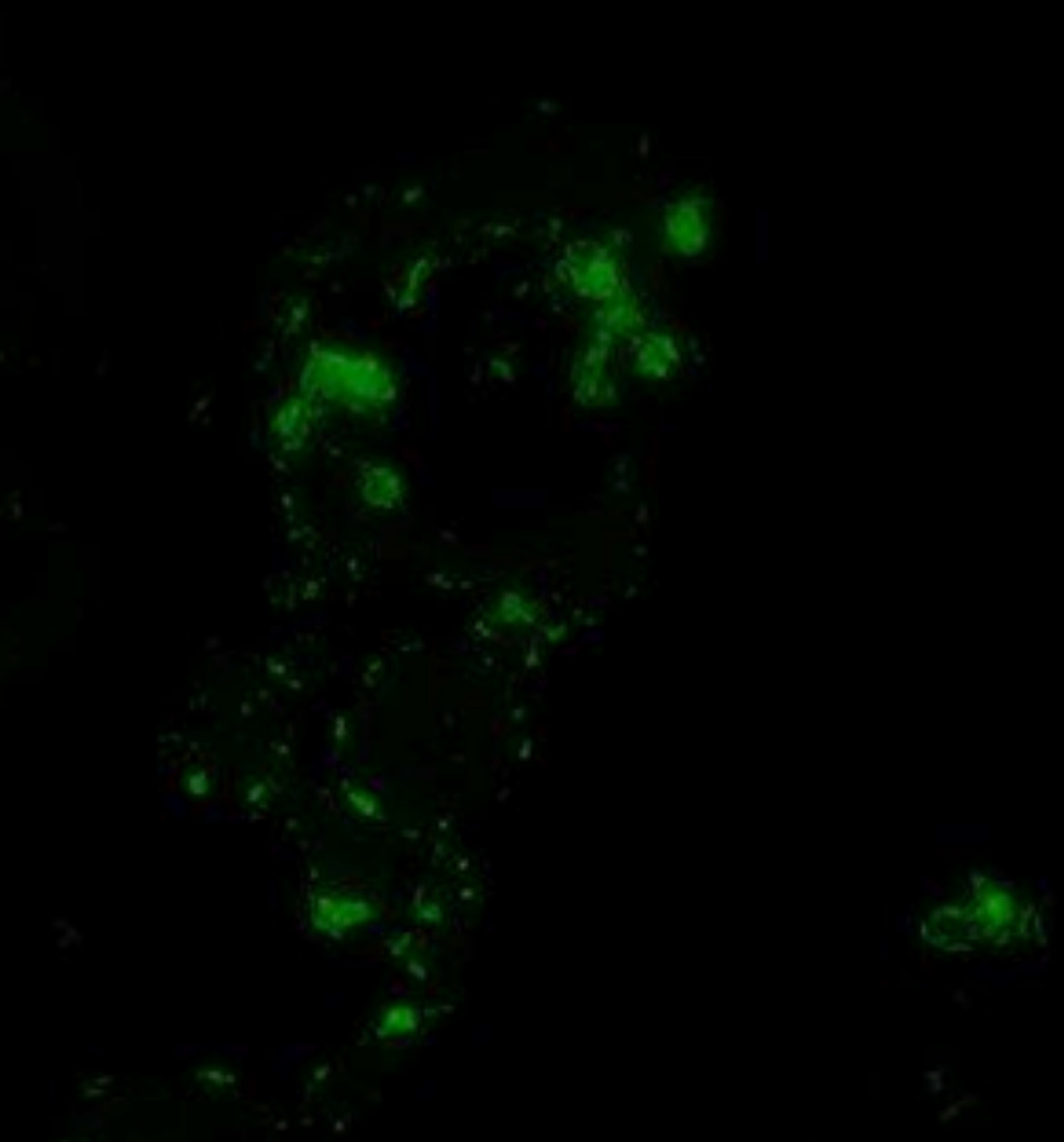

Supplement: Supplementary Figure S1 — Light micrographs of non-infected placental explants stained with Kinyoun at baseline (4 h). [file Data_Sheet_1.zip › Supplementary figures/Immunofluorescence S13-S57, S71-S73/Figure S54.jpg]

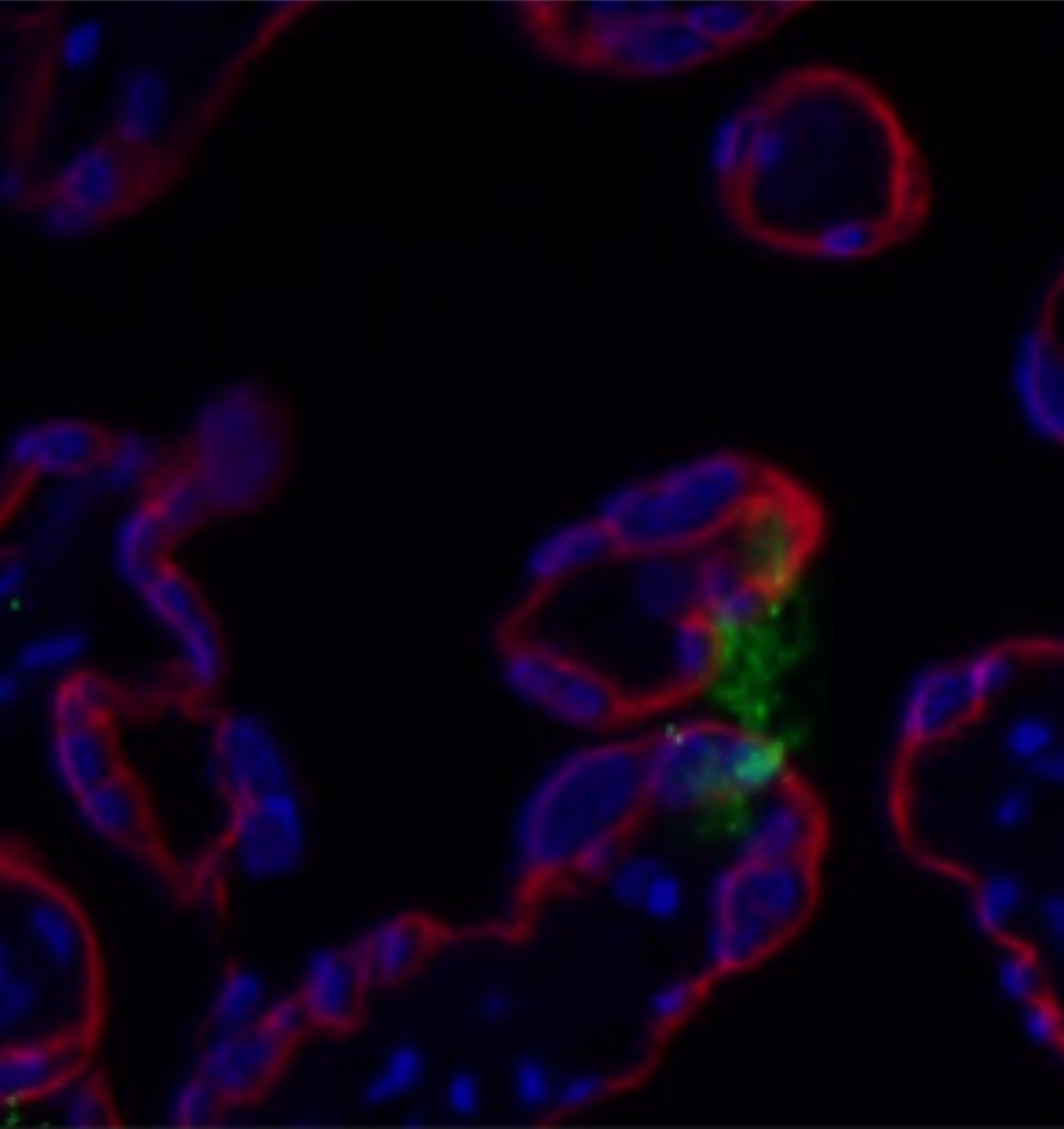

Supplement: Supplementary Figure S1 — Light micrographs of non-infected placental explants stained with Kinyoun at baseline (4 h). [file Data_Sheet_1.zip › Supplementary figures/Immunofluorescence S13-S57, S71-S73/Figure S55.jpg]

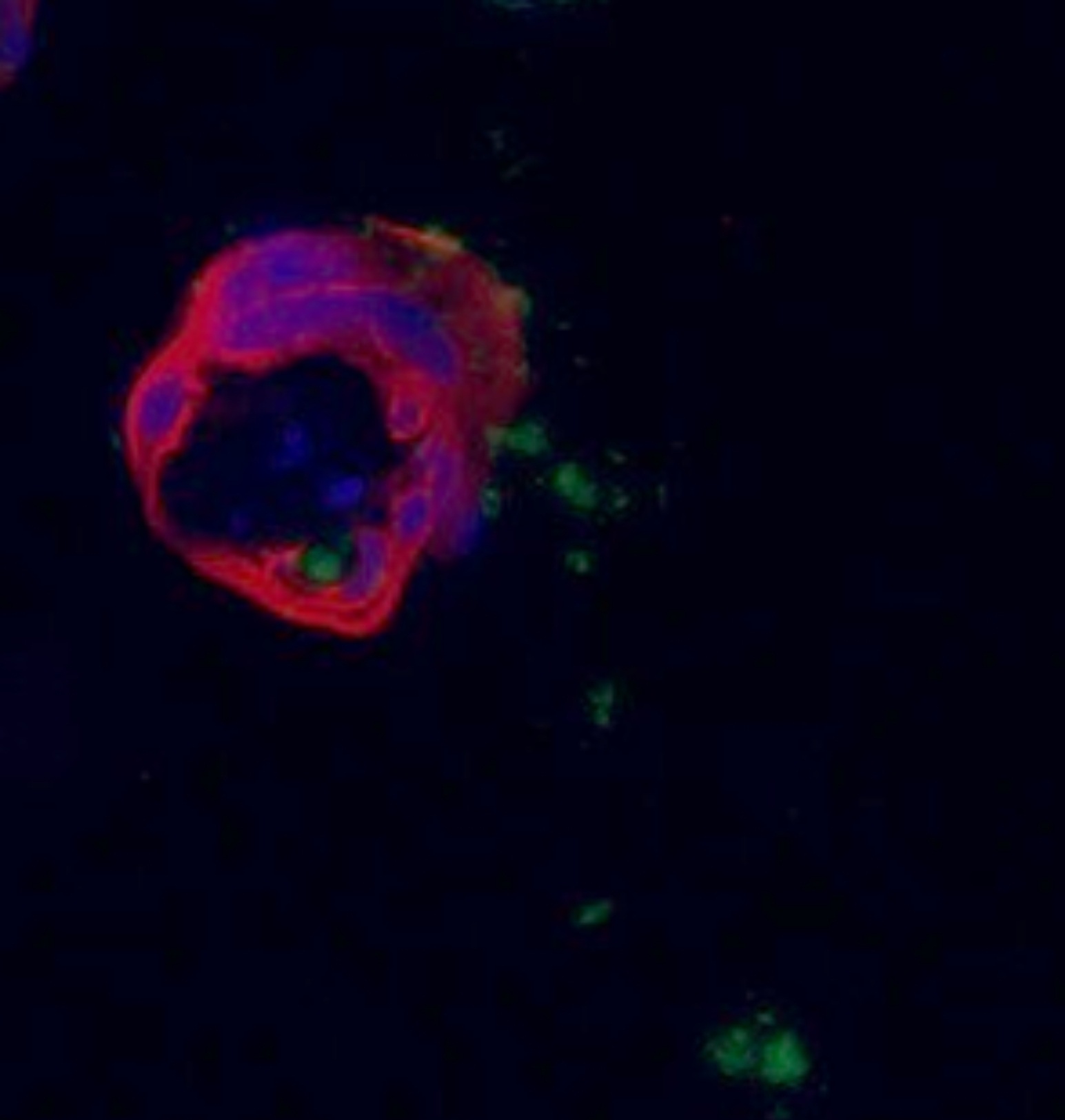

Supplement: Supplementary Figure S1 — Light micrographs of non-infected placental explants stained with Kinyoun at baseline (4 h). [file Data_Sheet_1.zip › Supplementary figures/Immunofluorescence S13-S57, S71-S73/Figure S56.jpg]

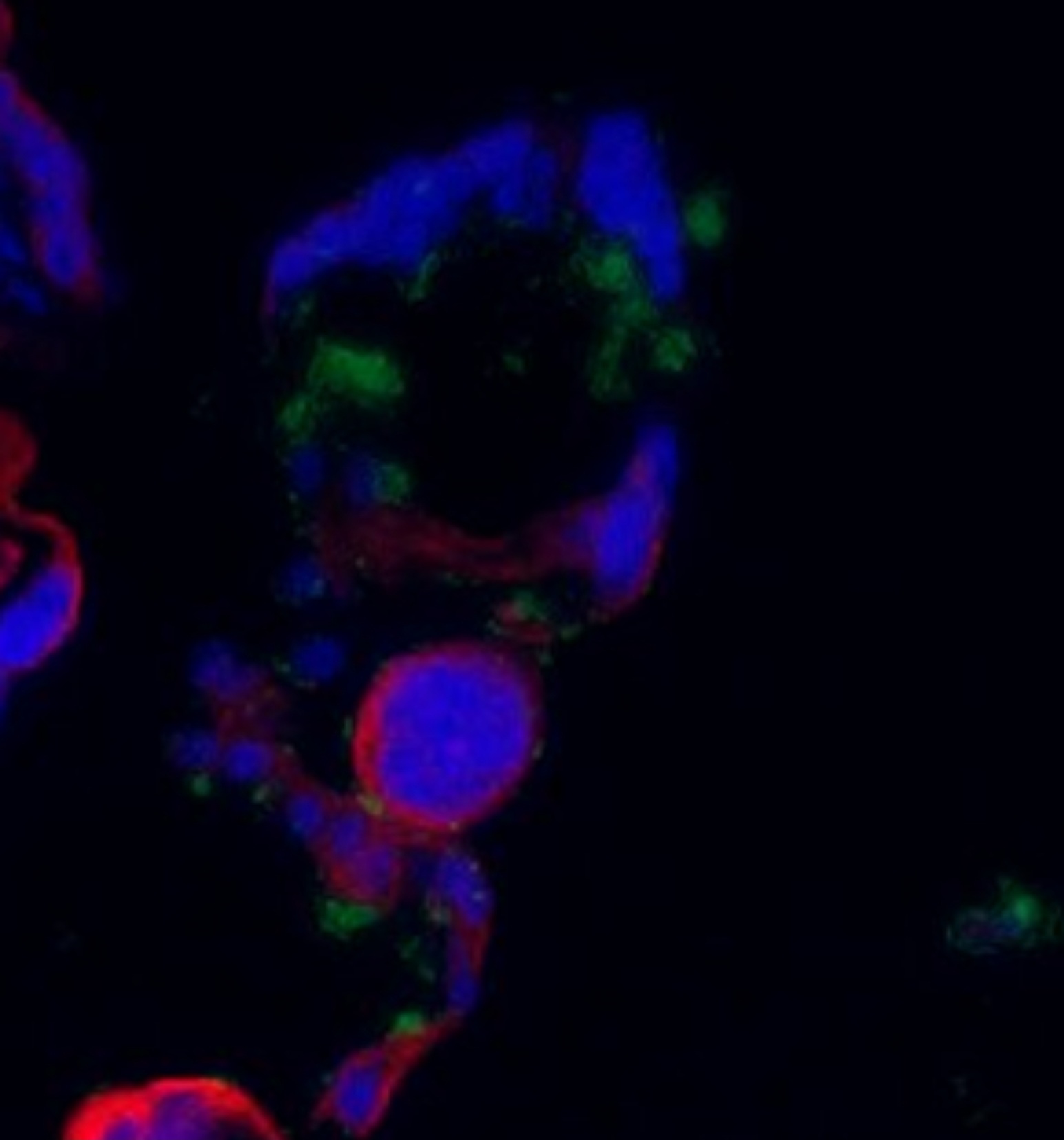

Supplement: Supplementary Figure S1 — Light micrographs of non-infected placental explants stained with Kinyoun at baseline (4 h). [file Data_Sheet_1.zip › Supplementary figures/Immunofluorescence S13-S57, S71-S73/Figure S57.jpg]

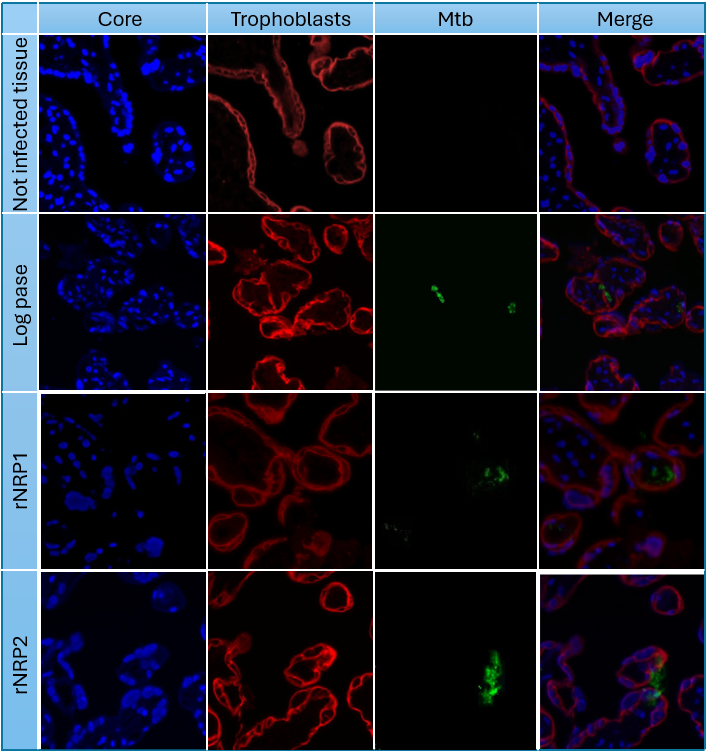

Supplement: Supplementary Figure S1 — Light micrographs of non-infected placental explants stained with Kinyoun at baseline (4 h). [file Data_Sheet_1.zip › Supplementary figures/Immunofluorescence S13-S57, S71-S73/Figure S71.tif]

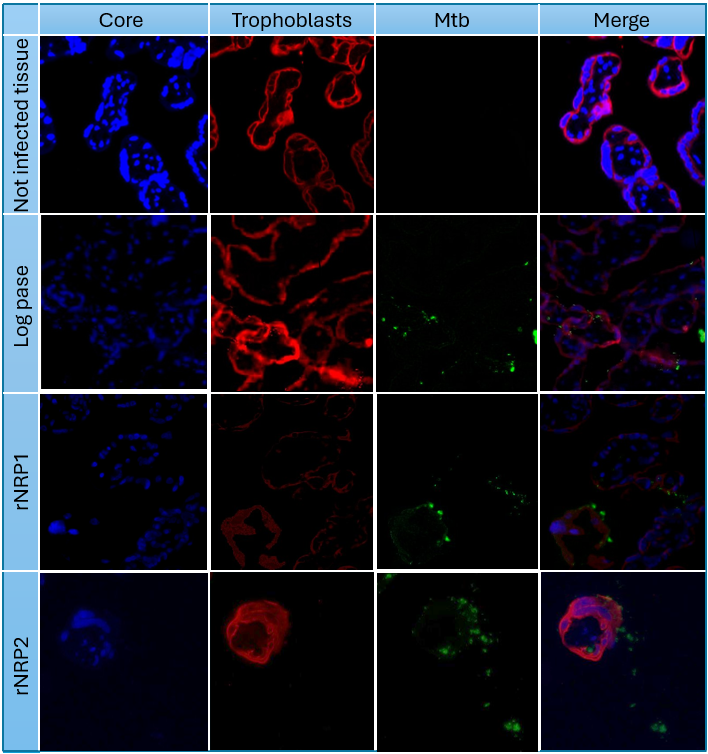

Supplement: Supplementary Figure S1 — Light micrographs of non-infected placental explants stained with Kinyoun at baseline (4 h). [file Data_Sheet_1.zip › Supplementary figures/Immunofluorescence S13-S57, S71-S73/Figure S72.tif]

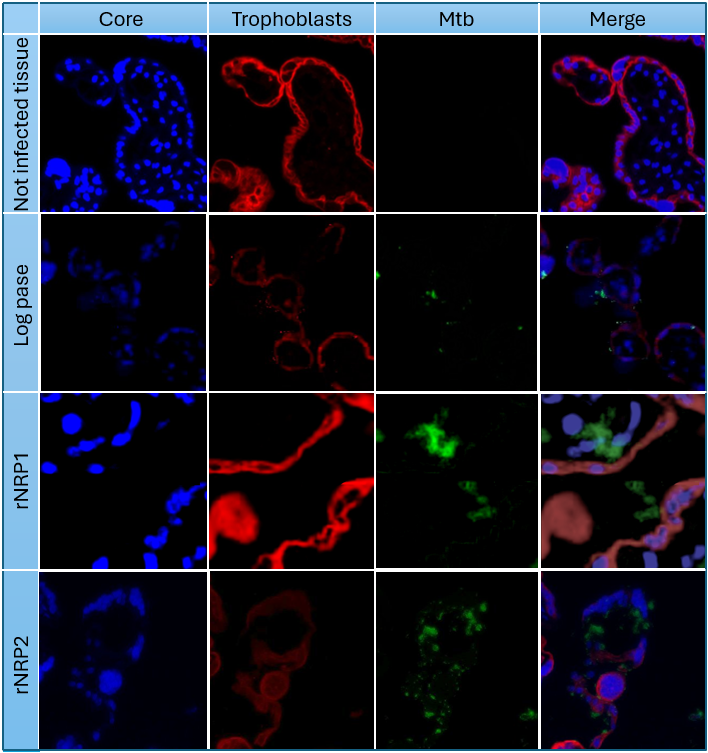

Supplement: Supplementary Figure S1 — Light micrographs of non-infected placental explants stained with Kinyoun at baseline (4 h). [file Data_Sheet_1.zip › Supplementary figures/Immunofluorescence S13-S57, S71-S73/Figure S73.tif]

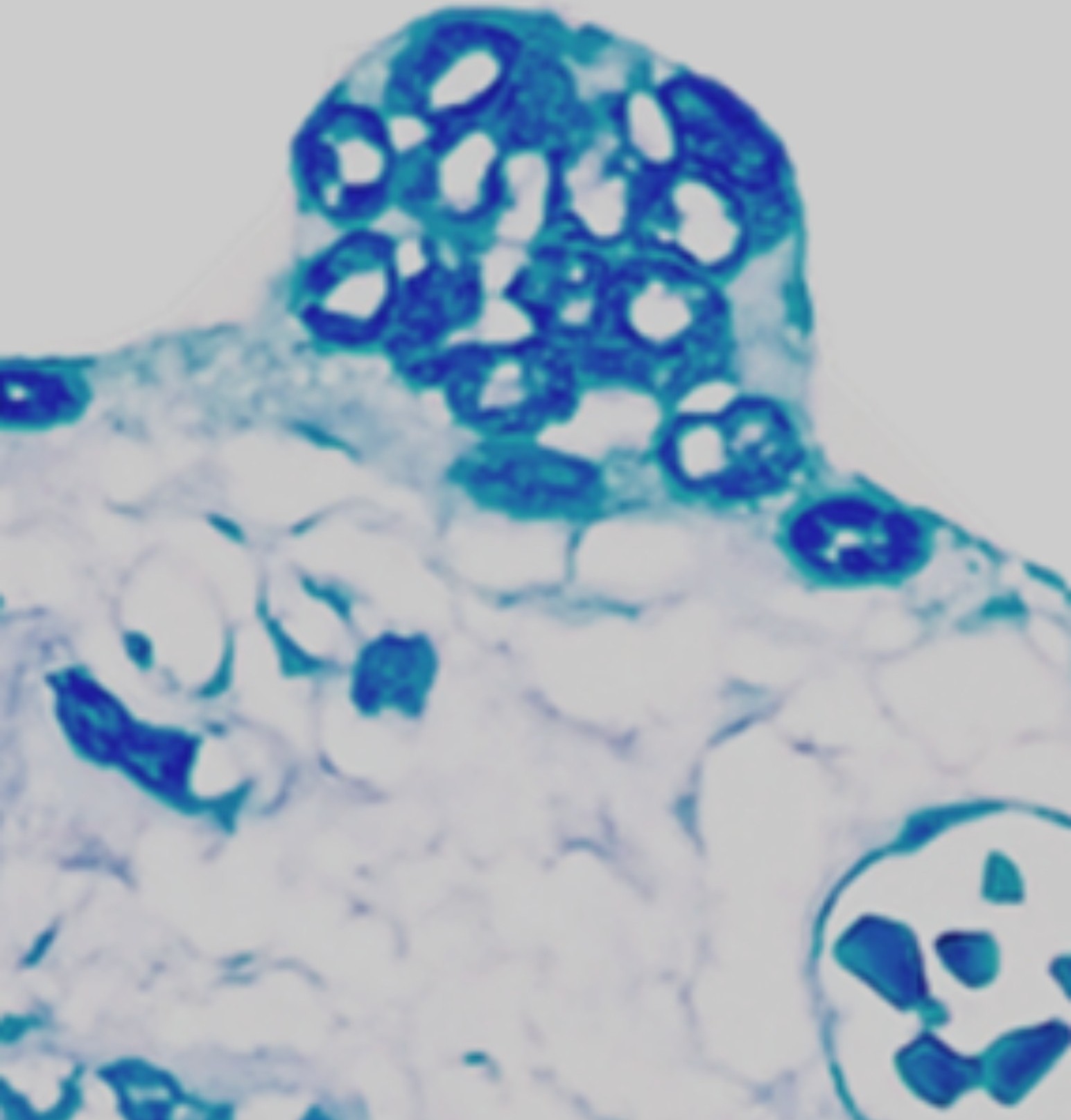

Supplement: Supplementary Figure S1 — Light micrographs of non-infected placental explants stained with Kinyoun at baseline (4 h). [file Data_Sheet_1.zip › Supplementary figures/Kinyoun S1-S12, S70/Figure S1.jpg]

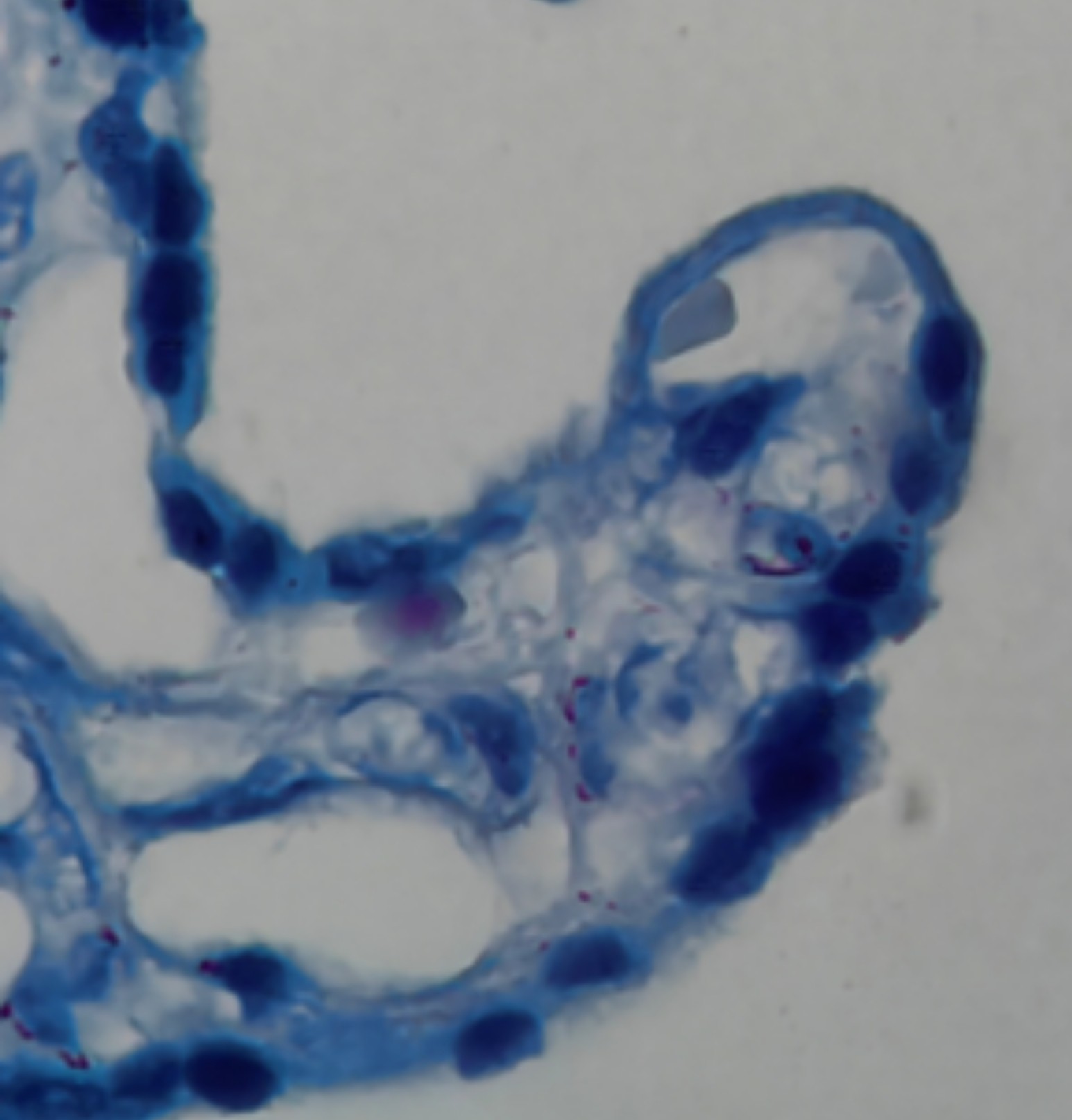

Supplement: Supplementary Figure S1 — Light micrographs of non-infected placental explants stained with Kinyoun at baseline (4 h). [file Data_Sheet_1.zip › Supplementary figures/Kinyoun S1-S12, S70/Figure S10.jpg]

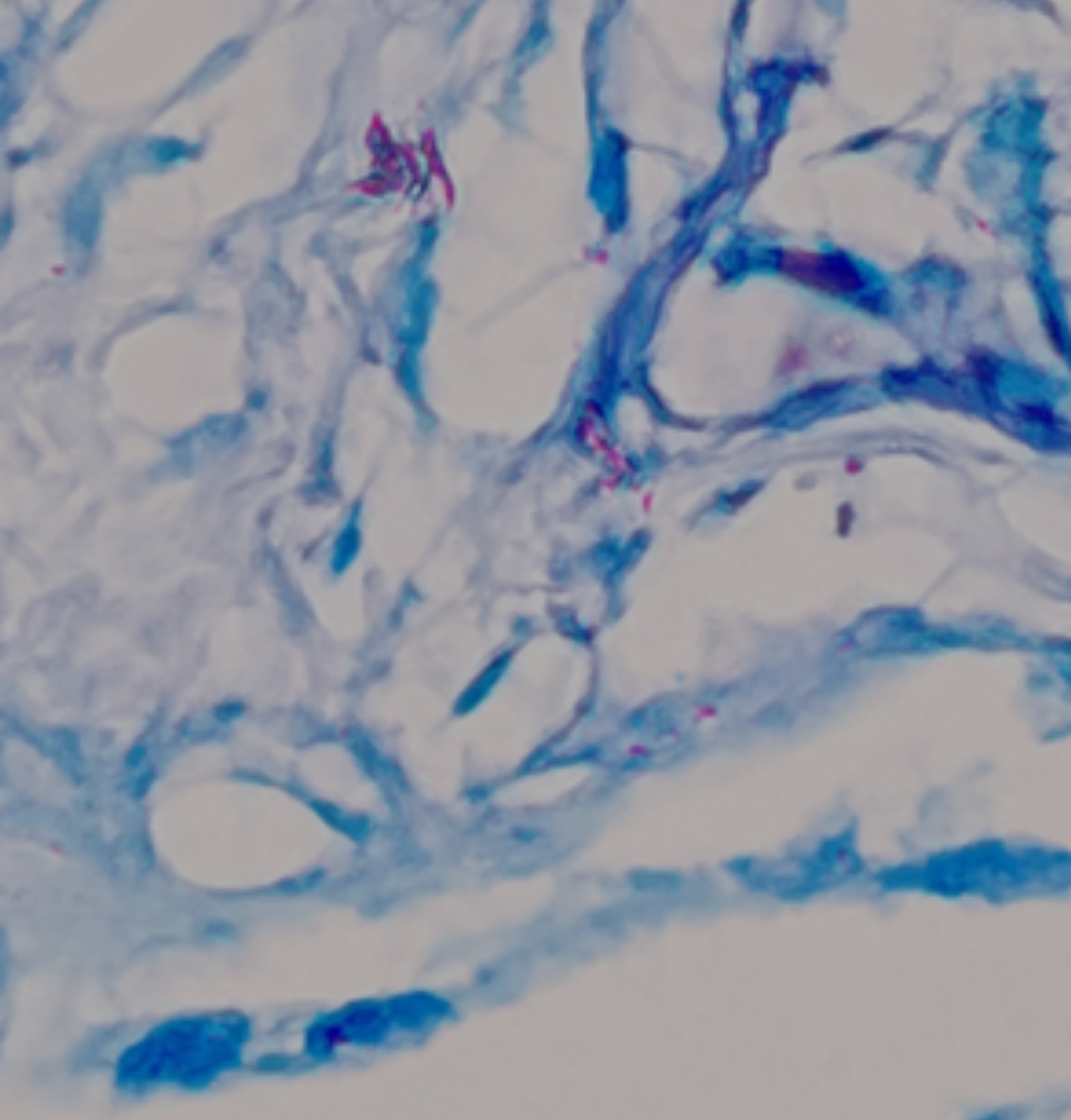

Supplement: Supplementary Figure S1 — Light micrographs of non-infected placental explants stained with Kinyoun at baseline (4 h). [file Data_Sheet_1.zip › Supplementary figures/Kinyoun S1-S12, S70/Figure S11.jpg]

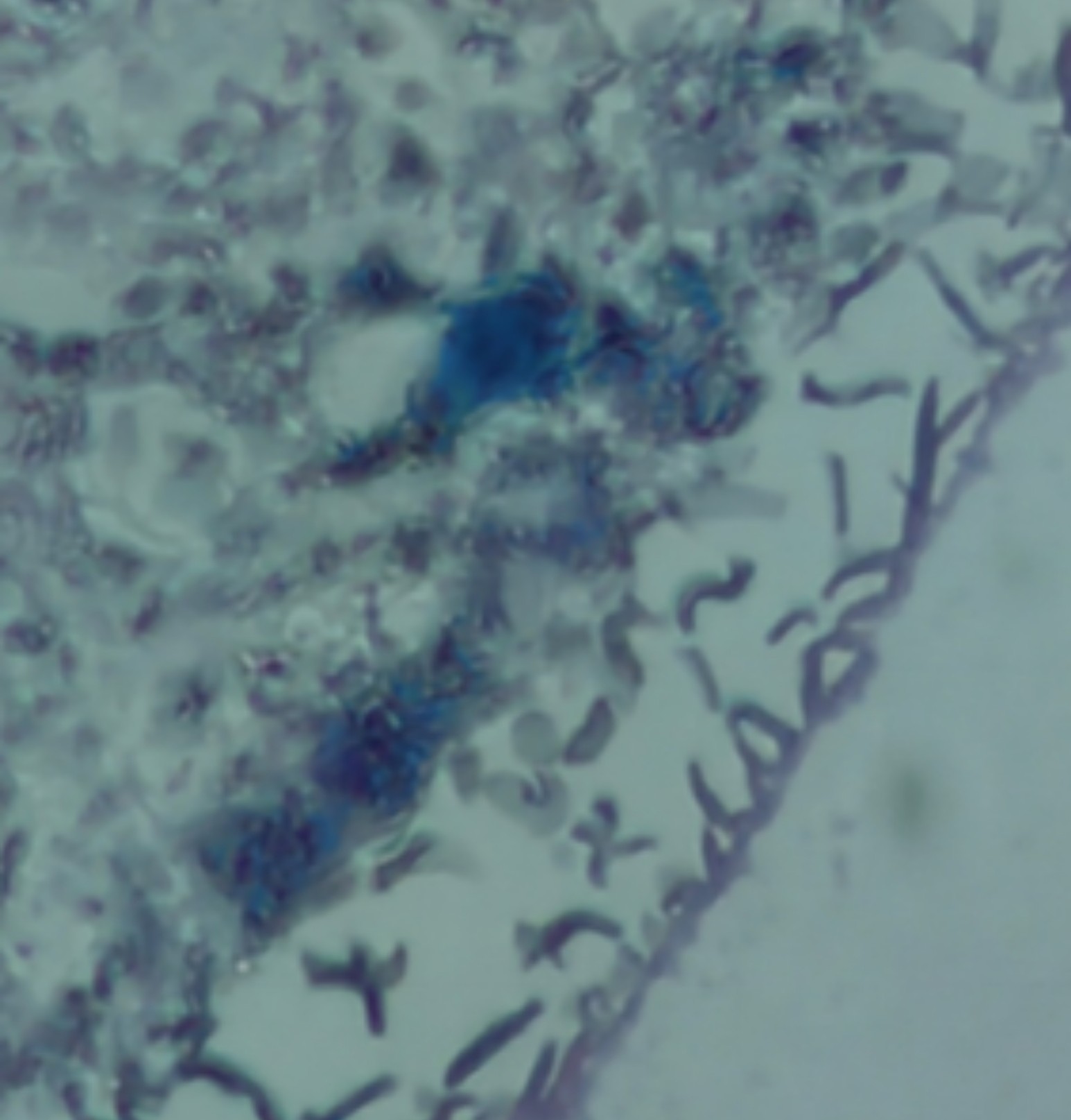

Supplement: Supplementary Figure S1 — Light micrographs of non-infected placental explants stained with Kinyoun at baseline (4 h). [file Data_Sheet_1.zip › Supplementary figures/Kinyoun S1-S12, S70/Figure S12.jpg]

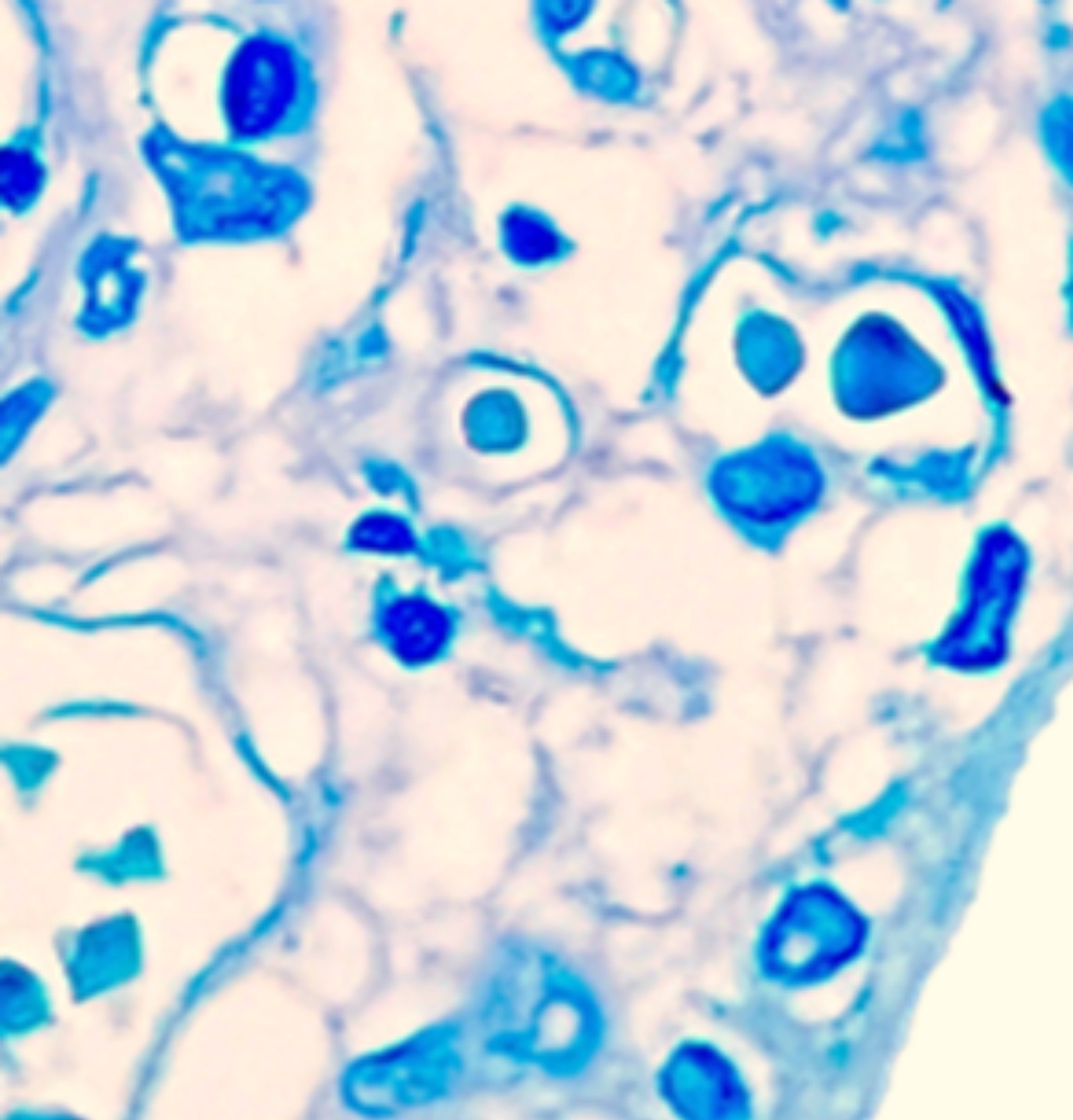

Supplement: Supplementary Figure S1 — Light micrographs of non-infected placental explants stained with Kinyoun at baseline (4 h). [file Data_Sheet_1.zip › Supplementary figures/Kinyoun S1-S12, S70/Figure S2.jpg]

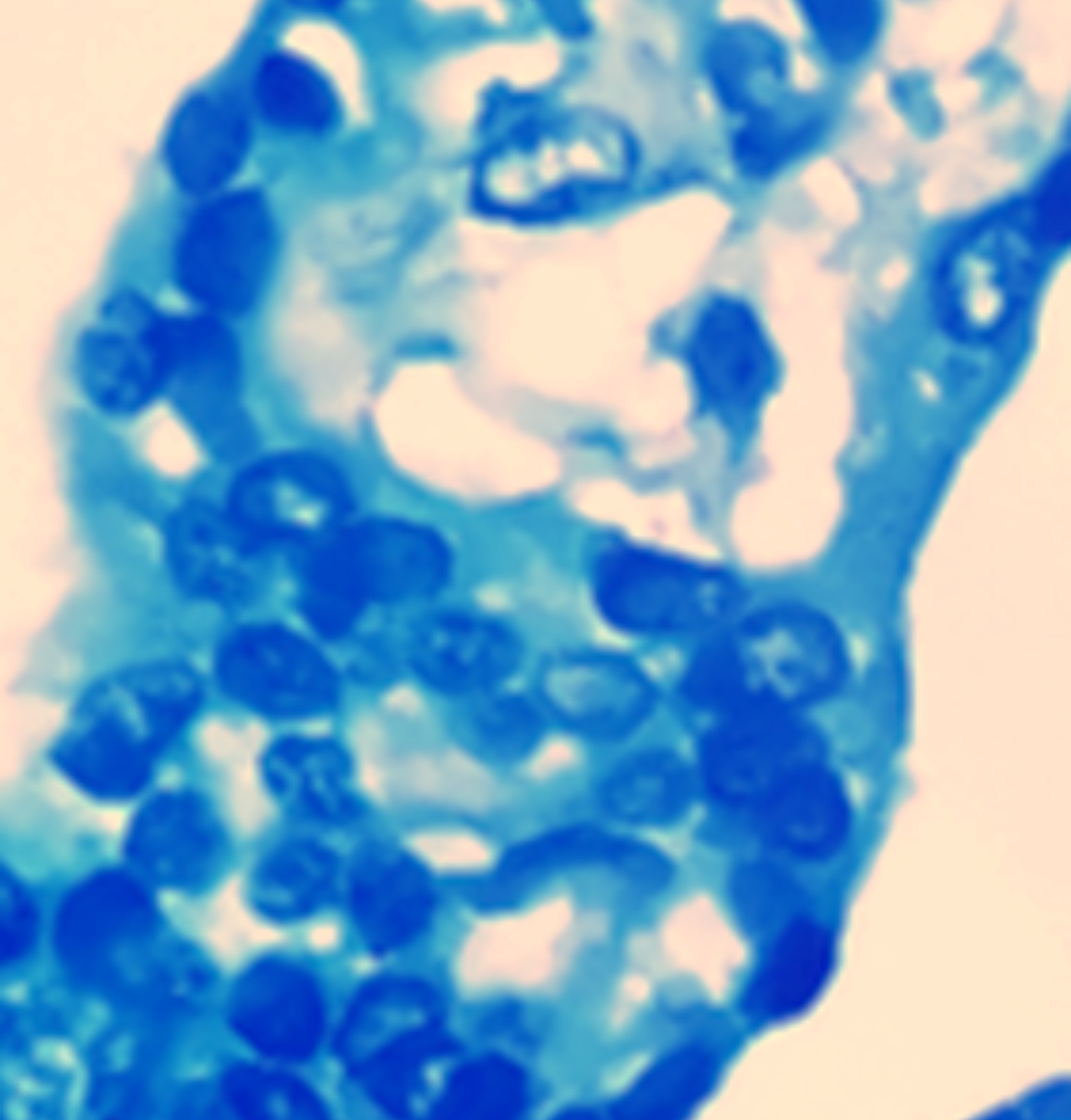

Supplement: Supplementary Figure S1 — Light micrographs of non-infected placental explants stained with Kinyoun at baseline (4 h). [file Data_Sheet_1.zip › Supplementary figures/Kinyoun S1-S12, S70/Figure S3.jpg]

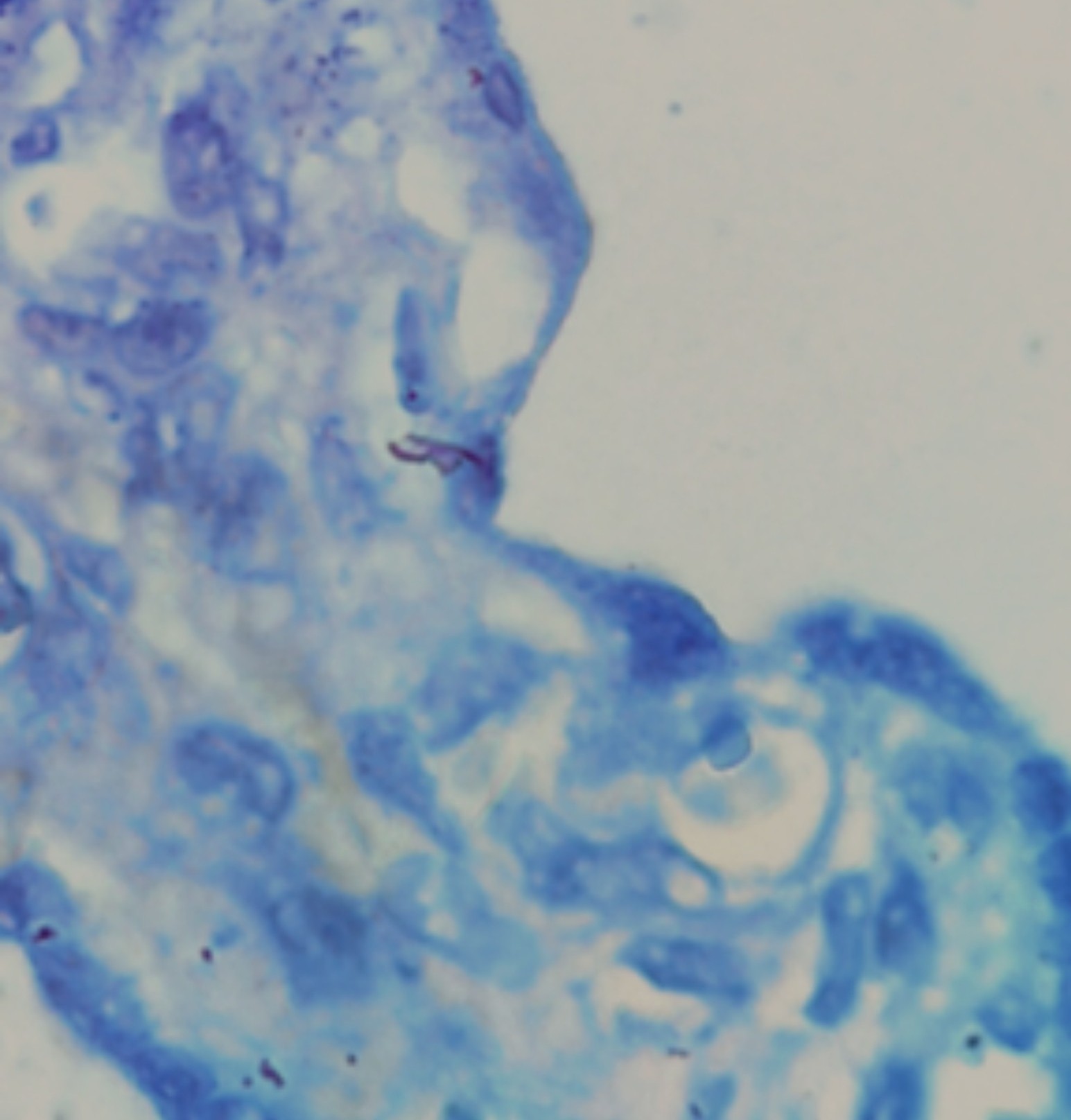

Supplement: Supplementary Figure S1 — Light micrographs of non-infected placental explants stained with Kinyoun at baseline (4 h). [file Data_Sheet_1.zip › Supplementary figures/Kinyoun S1-S12, S70/Figure S4.jpg]

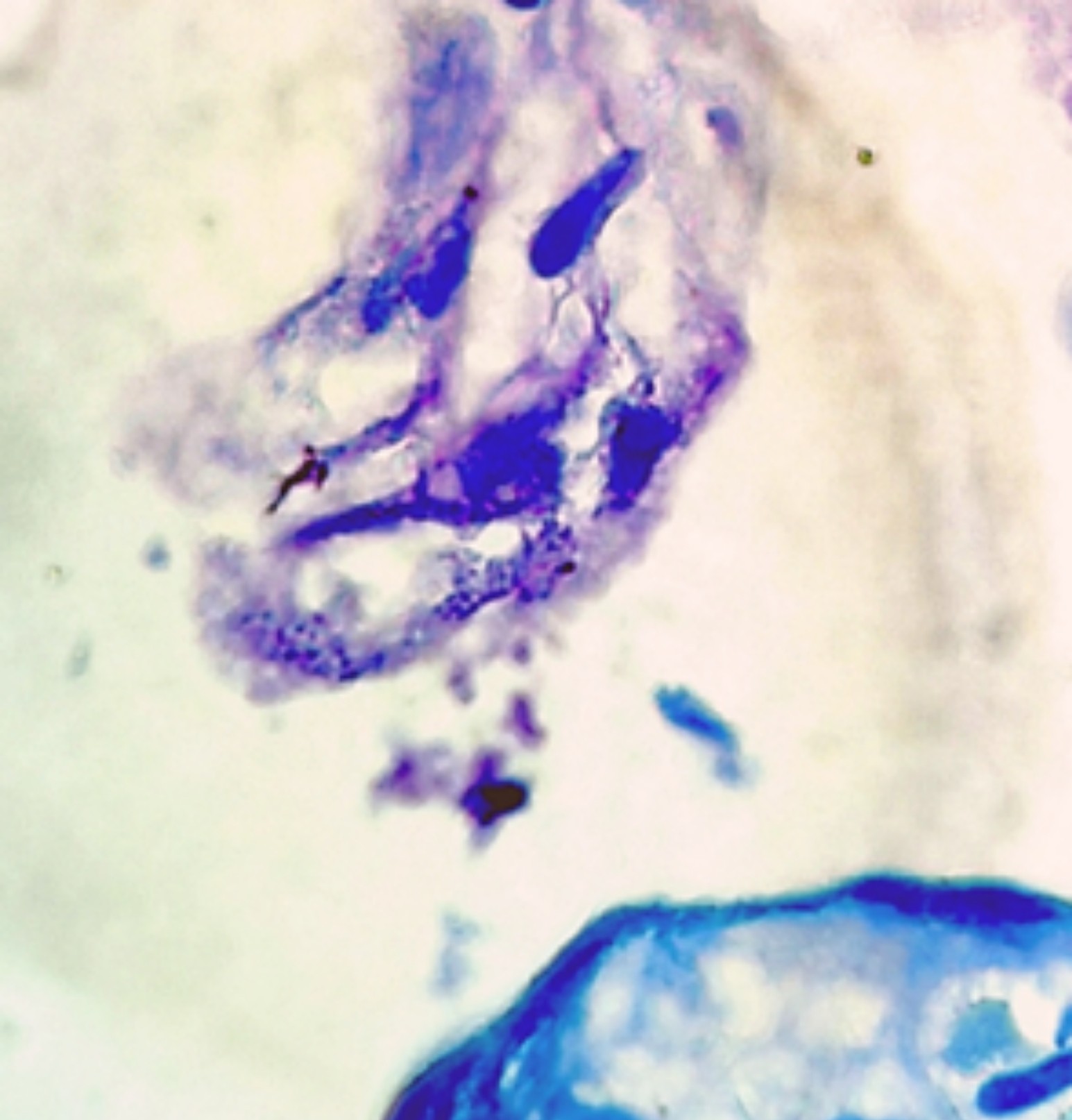

Supplement: Supplementary Figure S1 — Light micrographs of non-infected placental explants stained with Kinyoun at baseline (4 h). [file Data_Sheet_1.zip › Supplementary figures/Kinyoun S1-S12, S70/Figure S5.jpg]

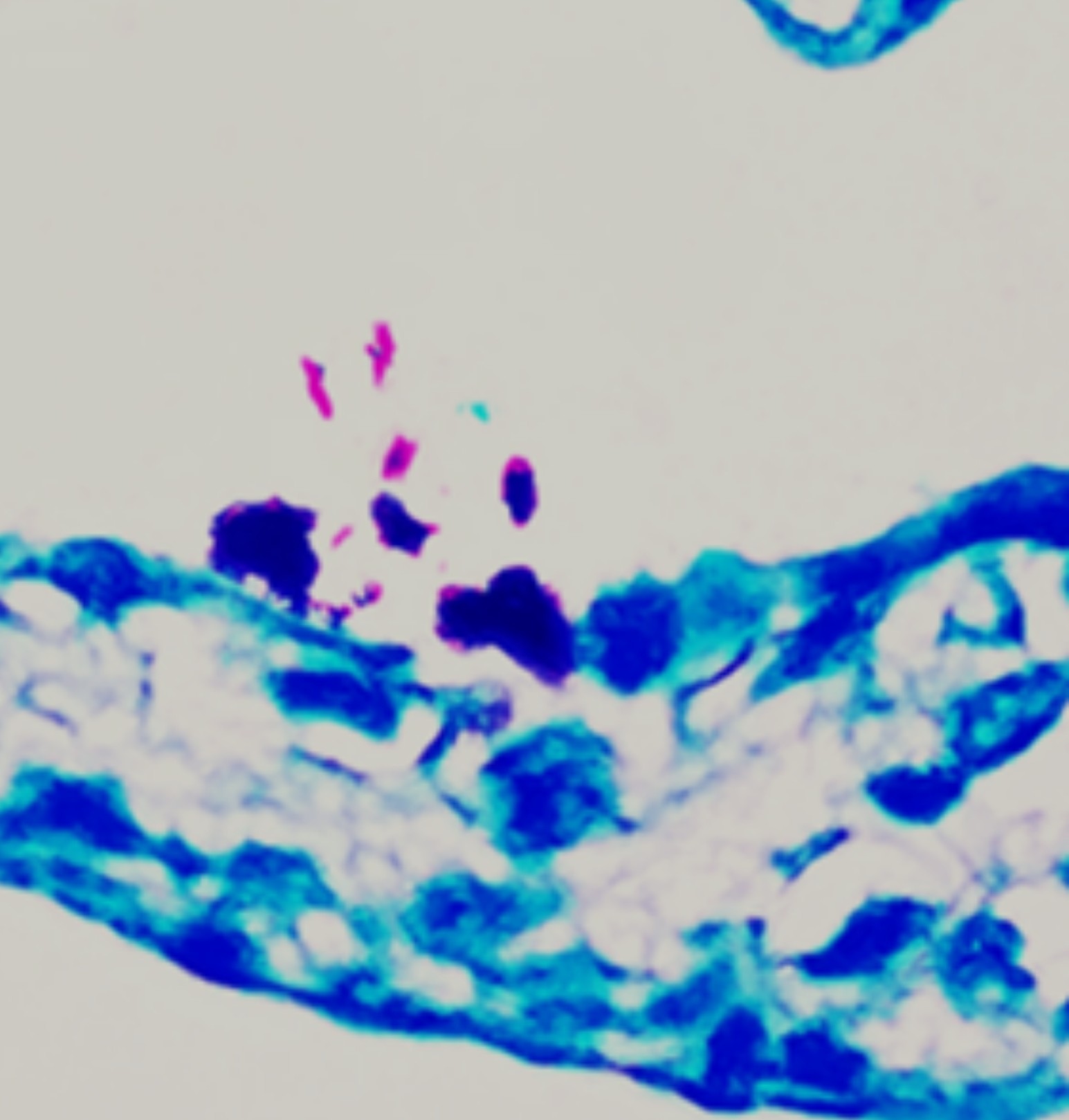

Supplement: Supplementary Figure S1 — Light micrographs of non-infected placental explants stained with Kinyoun at baseline (4 h). [file Data_Sheet_1.zip › Supplementary figures/Kinyoun S1-S12, S70/Figure S6.jpg]

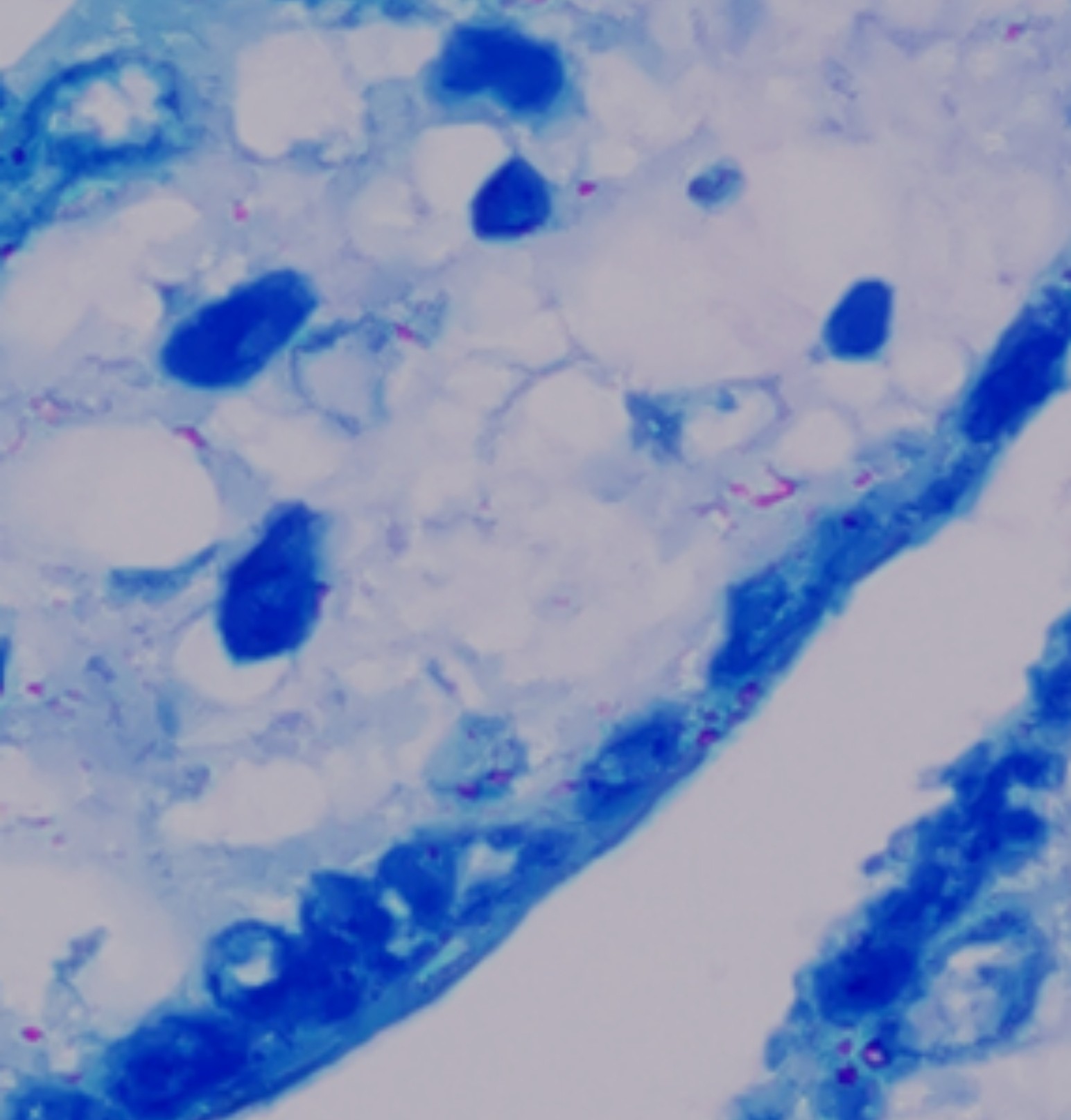

Supplement: Supplementary Figure S1 — Light micrographs of non-infected placental explants stained with Kinyoun at baseline (4 h). [file Data_Sheet_1.zip › Supplementary figures/Kinyoun S1-S12, S70/Figure S7.jpg]

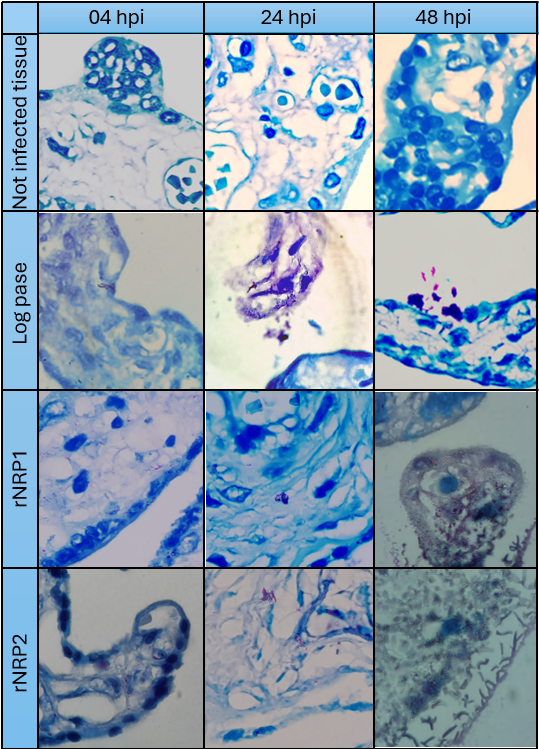

Supplement: Supplementary Figure S1 — Light micrographs of non-infected placental explants stained with Kinyoun at baseline (4 h). [file Data_Sheet_1.zip › Supplementary figures/Kinyoun S1-S12, S70/Figure S70.tif]

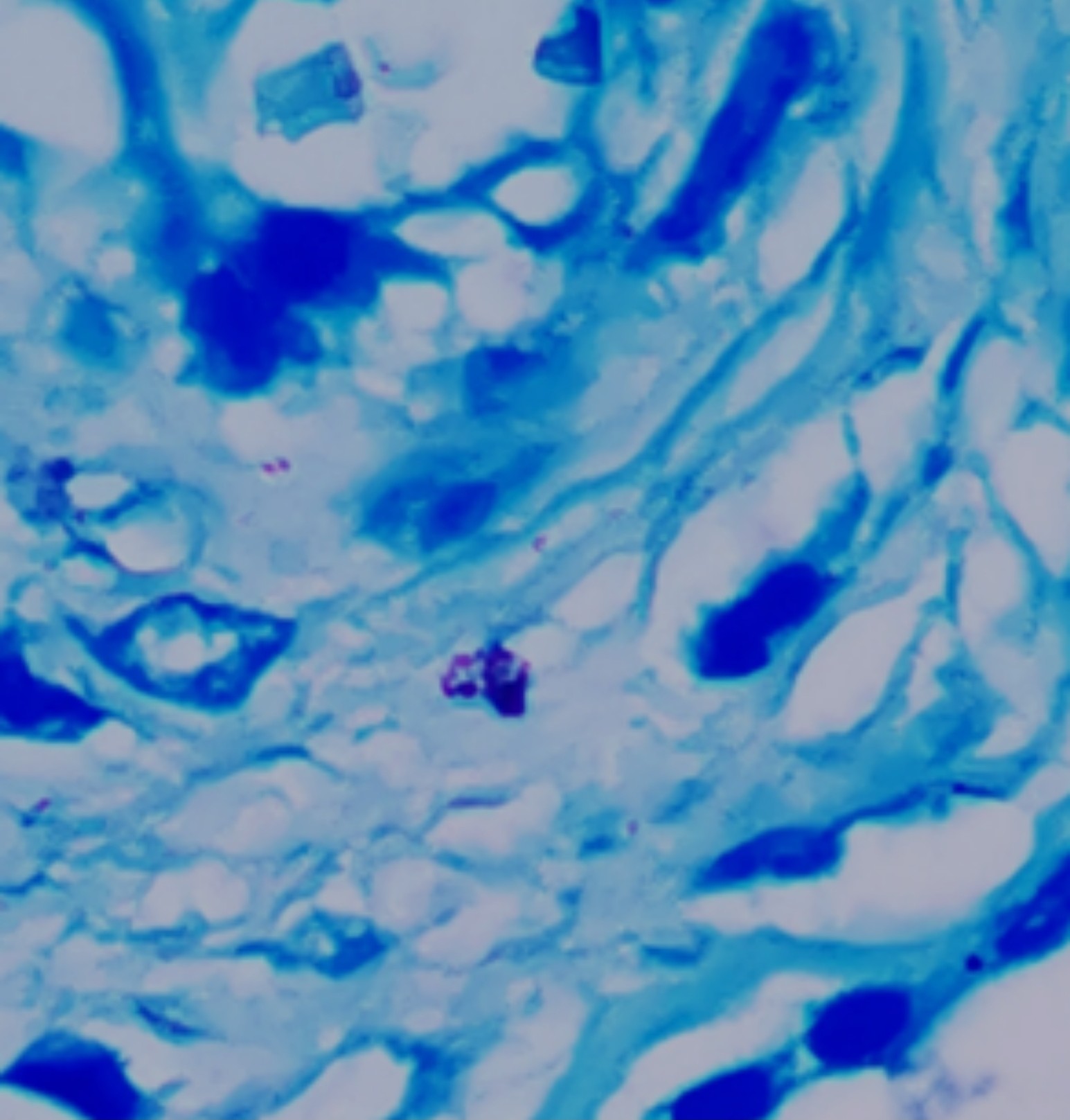

Supplement: Supplementary Figure S1 — Light micrographs of non-infected placental explants stained with Kinyoun at baseline (4 h). [file Data_Sheet_1.zip › Supplementary figures/Kinyoun S1-S12, S70/Figure S8.jpg]

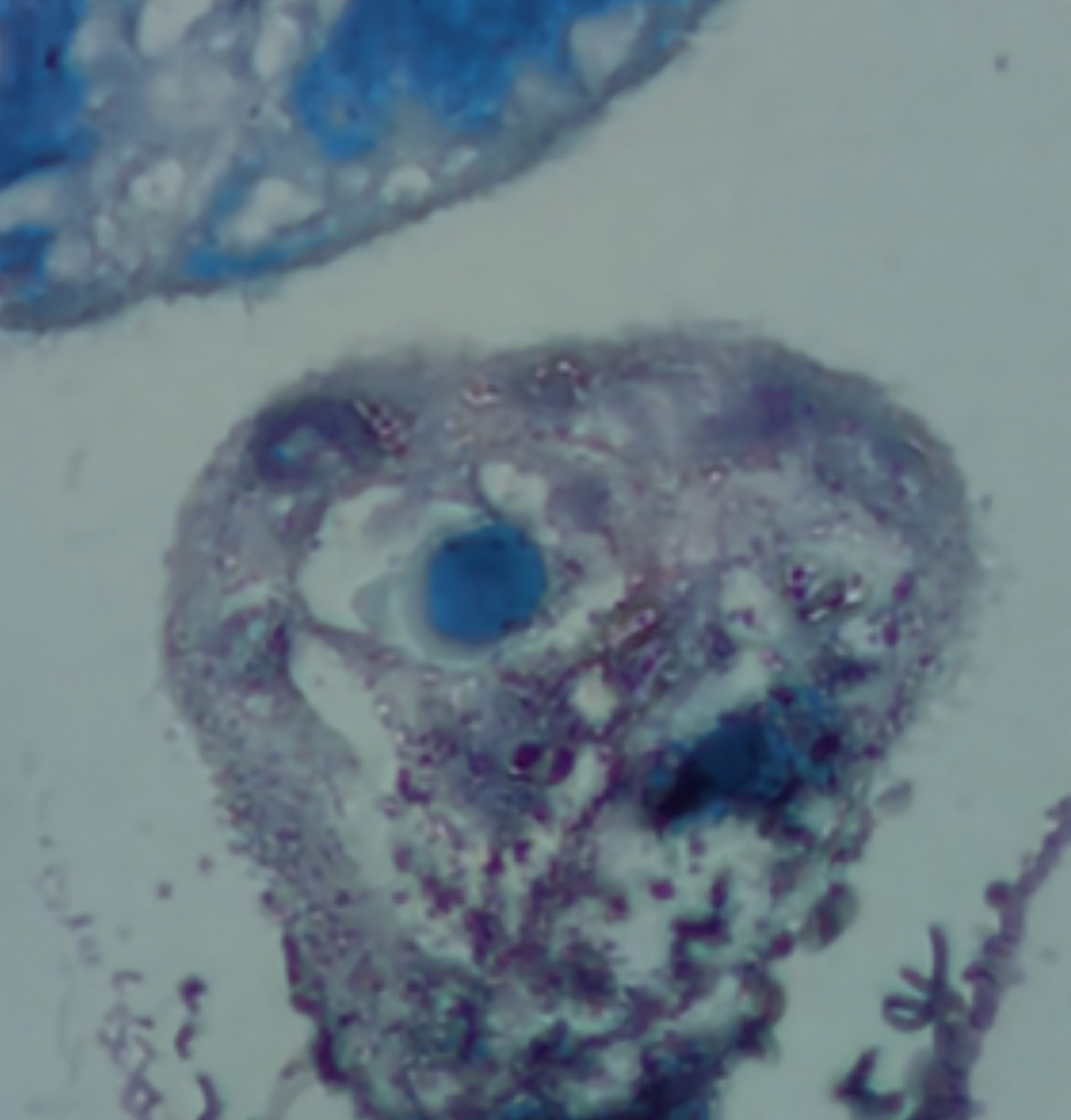

Supplement: Supplementary Figure S1 — Light micrographs of non-infected placental explants stained with Kinyoun at baseline (4 h). [file Data_Sheet_1.zip › Supplementary figures/Kinyoun S1-S12, S70/Figure S9.jpg]
